# Lung dendritic-cell metabolism underlies susceptibility to viral infection in diabetes

**DOI:** 10.1038/s41586-023-06803-0

**Published:** 2023-12-13

**Authors:** Samuel Philip Nobs, Aleksandra A. Kolodziejczyk, Lital Adler, Nir Horesh, Christine Botscharnikow, Ella Herzog, Gayatree Mohapatra, Sophia Hejndorf, Ryan-James Hodgetts, Igor Spivak, Lena Schorr, Leviel Fluhr, Denise Kviatcovsky, Anish Zacharia, Suzanne Njuki, Dinorah Barasch, Noa Stettner, Mally Dori-Bachash, Alon Harmelin, Alexander Brandis, Tevie Mehlman, Ayelet Erez, Yiming He, Sara Ferrini, Jens Puschhof, Hagit Shapiro, Manfred Kopf, Arieh Moussaieff, Suhaib K. Abdeen, Eran Elinav

**Affiliations:** 1 Systems Immunology Department, Weizmann Institute of Science, Rehovot, Israel; 2 Department of Molecular Cell Biology, Weizmann Institute of Science, Rehovot, Israel; 3 Department of General Surgery and Transplantations, Sheba Medical Center, Ramat Gan, Israel; 4 Faculty of Medicine, Tel Aviv University, Tel Aviv, Israel; 5 Division of Microbiome & Cancer, DKFZ, Heidelberg, Germany; 6 Faculty of Biosciences, Heidelberg University, Heidelberg, Germany; 7 The Institute for Drug Research, Hebrew University of Jerusalem, Jerusalem, Israel; 8 Department of Veterinary Resources, Weizmann Institute of Science, Rehovot, Israel; 9 Department of Biological Services, Weizmann Institute of Science, Rehovot, Israel; 10 Institute of Molecular Health Sciences, ETH Zurich, Zurich, Switzerland; 11 Present Address: International Institute of Molecular and Cellular Biology, Warsaw, Poland

**Keywords:** Antimicrobial responses, Metabolic disorders

## Abstract

People with diabetes feature a life-risking susceptibility to respiratory viral infection, including influenza and SARS-CoV-2 (ref. ^1^), whose mechanism remains unknown. In acquired and genetic mouse models of diabetes, induced with an acute pulmonary viral infection, we demonstrate that hyperglycaemia leads to impaired costimulatory molecule expression, antigen transport and T cell priming in distinct lung dendritic cell (DC) subsets, driving a defective antiviral adaptive immune response, delayed viral clearance and enhanced mortality. Mechanistically, hyperglycaemia induces an altered metabolic DC circuitry characterized by increased glucose-to-acetyl-CoA shunting and downstream histone acetylation, leading to global chromatin alterations. These, in turn, drive impaired expression of key DC effectors including central antigen presentation-related genes. Either glucose-lowering treatment or pharmacological modulation of histone acetylation rescues DC function and antiviral immunity. Collectively, we highlight a hyperglycaemia-driven metabolic-immune axis orchestrating DC dysfunction during pulmonary viral infection and identify metabolic checkpoints that may be therapeutically exploited in mitigating exacerbated disease in infected diabetics.

## Abstract (web summary)

Hyperglycaemia leads to impaired costimulatory molecule expression, antigen transport and T cell priming in distinct lung dendritic cell subsets, driving a defective antiviral adaptive immune response, delayed viral clearance and enhanced mortality.

## Main

Diabetes mellitus constitutes a major public health burden^2^, leading to a variety of complications secondary to a failure to maintain glucose control^3^. One key unexplained phenomenon in both type 1 and 2 diabetes is a markedly increased susceptibility to respiratory infection^1^, leading to significantly enhanced morbidity and mortality following infection with viruses such as influenza and other lung pathogens^1^. This phenomenon has recently gained even more pressing importance, with the realization that diabetes constitutes a common comorbidity among patients with severe SARS-CoV-2 (ref. ^4^) and promotes markedly worsened pulmonary disease, higher mortality^5,6^ and risk of serious post-vaccination breakthrough infection^7^. Studies in experimental animal models evaluating the links between respiratory infection and diabetes have been sparse^8^, and do not offer a mechanism explaining this pronounced risk^9^. We sought to explore drivers of diabetes-induced susceptibility to pulmonary viral infection.

## Diabetes exacerbates viral lung disease

**Fig. 1:**
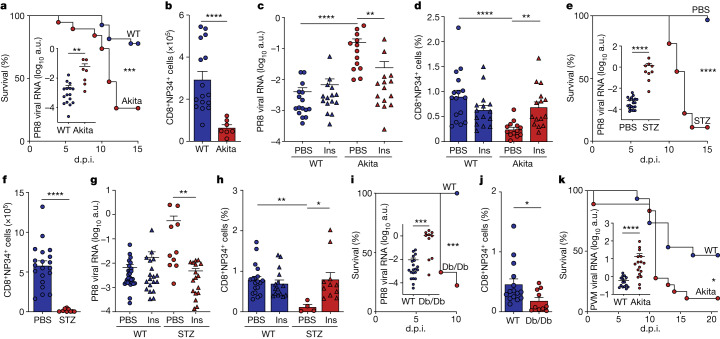
Diabetes exacerbates lung viral infection. **a**,**b**, WT (*n* = 27) and Akita (*n* = 19) mice infected with 200 plaque-forming units (pfu) PR8, log-rank Mantel–Cox test. **a**, Survival. Inset, lung PR8 RNA 10 d.p.i., WT (*n* = 16) and Akita (*n* = 7) mice infected with 50 pfu PR8, two-sided Mann–Whitney *U*-test. **b**, Lung NP34-tetramer^+^CD8^+^ T cells, two-sided Mann–Whitney *U*-test. **c**,**d**, Mice infected with 50 pfu PR8, treated with insulin (Ins)/phosphate-buffered saline (PBS): WT+PBS (*n* = 16), WT+Ins (*n* = 15), Akita+PBS (*n* = 13), Akita+Ins (*n* = 15), Kruskal–Wallis test with Dunn’s correction, two pooled experiments. **c**, Lung PR8 RNA. **d**, Lung NP34-tetramer^+^CD8^+^ T cells. **e**,**f**, Mice infected with 200 pfu PR8, administered STZ (*n* = 18 for **e**, *n* = 9 for **f**) or PBS (*n* = 30 for **e**, *n* = 20 for **f**). **e**, Survival, log-rank Mantel–Cox test. Inset, lung PR8 RNA 10 d.p.i., mice infected with 50 pfu PR8, administered STZ (*n* = 9) or PBS (*n* = 20), two-sided Mann–Whitney *U*-test. **f**, Lung NP34-tetramer^+^CD8^+^ T cells, two-sided Mann–Whitney *U*-test. **g**,**h**, Mice infected with 50 pfu PR8, administered STZ or PBS, treated with Ins/PBS: PBS+PBS (*n* = 27 for **g**, *n* = 19 for **h**), PBS+Ins (*n* = 19 for **g**, *n* = 15 for **h**), STZ+PBS (*n* = 10 for **g**, *n* = 4 for **h**), STZ+Ins (*n* = 17 for **g**, *n* = 11 for **h**), Kruskal–Wallis test with Dunn’s correction. **g**, PR8 RNA, three pooled experiments. **h**, Lung NP34-tetramer^+^CD8^+^ T cells, two pooled experiments. **i**,**j**, Db/Db (*n* = 9 for **i**, *n* = 10 for **j**) and WT (*n* = 10 for **i**, *n* = 15 for **j**) mice infected with 200 pfu PR8. **i**, Survival, log-rank Mantel–Cox test. Inset, lung PR8 RNA 10 d.p.i., WT (*n* = 20) and Db/Db (*n* = 10) mice infected with 50 pfu PR8, three pooled experiments, two-sided Mann–Whitney *U*-test. **j**, NP34-tetramer^+^CD8^+^ T cells, two-sided Mann–Whitney *U*-test. **k**, Survival, WT (*n* = 15) and Akita (*n* = 18) mice infected with 200 pfu PVM, log-rank Mantel–Cox test. Inset, lung PVM RNA 10 d.p.i., WT (*n* = 15) and Akita (*n* = 17) mice infected with 50 pfu PVM, two-sided Mann–Whitney *U*-test. All *P* values are indicated in Supplementary Table 1. All data mean+s.e.m. a.u., Arbitrary units.

**Extended Data Fig. 1:**
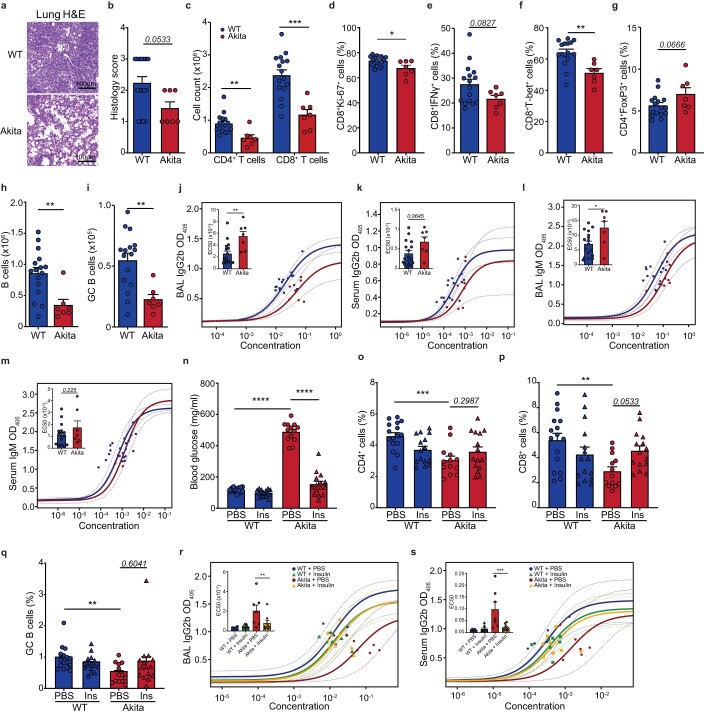
Diabetes-associated susceptibility to respiratory viral infection is associated with broadly impaired lung adaptive immunity. **a-m**, WT (*n* = 16) and Akita mice (*n* = 7) infected with 50pfu PR8, analyzed at 10 d.p.i. **a-b**, Lung histology, two-sided Mann Whitney *U*-test. **c**, Lung CD4^+^ T cells, two-sided Mann Whitney *U*-test, lung CD8^+^ T cells, two-sided unpaired *t*-test. **d**, Lung Ki-67^+^CD8^+^ T cells, two-sided unpaired *t*-test. **e**, Lung IFNγ^+^CD8^+^ T cells, two-sided unpaired *t*-test. **f**, Lung T-bet^+^CD8^+^ T cells, two-sided Mann Whitney *U*-test. **g**, Lung FoxP3^+^CD4^+^ T cells, two-sided unpaired *t*-test. **h**, Lung B cells, two-sided Mann Whitney *U*-test. **i**, GC B cells, two-sided unpaired *t*-test. **j-k**, virus specific IgG2b, **j**, BAL, **k**, serum, two-sided Mann-Whitney U-test for EC50. **l-m**, virus-specific IgM, **l**, BAL **m**, serum, two-sided unpaired *t*-test for EC50. **n-s**, Mice infected with 50 pfu PR8, treated with insulin/PBS: WT+PBS (*n* = 16), WT+Ins (*n* = 15), Akita+PBS (*n* = 13), Akita+Ins (*n* = 15), pooled data from 2 experiments. **n**, Blood glucose. **o**, Lung CD4^+^ T cells, one-way ANOVA and Holm Sidak correction. **p**, Lung CD8^+^ T cells, Kruskal-Wallis and Dunn’s correction. **q**, Lung GC B cells, Kruskal-Wallis and Dunn’s correction. **r-s**, Virus-specific IgG2b, **r**, BAL, **s**, serum, Akita+PBS (*n* = 6), Akita+Ins (*n* = 9), one-way ANOVA with Tukey correction on EC50. All data mean+s.e.m. GC, Germinal centre.

Exact statistical values of all experiments are provided in Supplementary Table 1. We began our investigation by assessing disease severity and immune responses in models of hyperglycaemic mice acutely infected with the H1N1 influenza A/Puerto Rico/8/1934 (PR8) influenza A virus (IAV). Akita mice, mutated in the *Ins2* gene, gradually developed systemic hyperglycaemia (Supplementary Information 2a,b) at around 6 weeks of age. Free fluid volume (Supplementary Information 2c) and most other serum electrolytes (Supplementary Information 2d) remained unaltered. Following high-dose IAV infection—and, similar to diabetic humans^1^—Akita mice evidenced markedly enhanced mortality compared with wild-type (WT) littermate controls (Fig. 1a). Increased disease burden in hyperglycaemic mice manifested as significantly elevated viral titres at 10 days post infection (d.p.i.) (Fig. 1a). Infiltration of lymphocytes in IAV-infected WT animals was markedly reduced in infected hyperglycaemic mice (Extended Data Fig. 1a,b), in parallel with impaired viral clearance (Fig. 1a). Collectively, this suggested that a potential defect in antiviral immunity may be linked to a hyperglycaemia-associated susceptibility to influenza infection.

Pulmonary interferon (IFN)-β (*Ifnb1*) gene expression (Supplementary Information 2e) and bronchoalveolar lavage (BAL) levels of several IFN-induced proteins (IP-10 and MCP-2; Supplementary Information 2f–g) were significantly elevated in hyperglycaemic compared with normoglycaemic IAV-infected mice, probably reflecting an intact type I IFN response secondary to increased viral titres and tissue damage. By contrast, the adaptive immune response in IAV-infected hyperglycaemic Akita mice was notable for a significant reduction in the number of total lung CD8^+^ and CD4^+^ T cells (Extended Data Fig. 1c) and virus-specific CD8^+^ T cells, using a major histocompatibility complex (MHC) tetramer directed against the immunodominant epitope NP34 (Fig. 1b and Supplementary Information 3a). Furthermore, the frequency of proliferating Ki-67^+^CD8^+^ T cells (Extended Data Fig. 1d and Supplementary Information 3b), IFNγ-producing CD8^+^ T cells (Extended Data Fig. 1e and Supplementary Information 3b,c) and T-bet^+^CD8^+^ T cells (Extended Data Fig. 1f) was reduced in hyperglycaemic animals compared with infected non-diabetic controls. A slight increase was observed in the frequency of lung FoxP3^+^CD4^+^ T regulatory cells (T_reg_ cells; Extended Data Fig. 1g). In addition, reduced numbers of total and CD95^+^ pulmonary germinal centre B cells (Extended Data Fig. 1h,i), coupled with significantly lower antiviral antibody titres in BAL (IgG2b and IgM; Extended Data Fig. 1j–m), were noted in infected hyperglycaemic mice compared with normoglycaemic controls. Importantly, glucose normalization in IAV-infected Akita mice using continuous insulin supplementation reduced blood glucose levels (Extended Data Fig. 1n), lowered viral titres (Fig. 1c) and rescued antiviral CD4^+^ and CD8^+^ T cells and antibody titres (Fig. 1d and Extended Data Fig. 1o–s). Taken together, these findings suggest that an increased susceptibility to respiratory infection during hyperglycaemia is coupled with a broadly impaired pulmonary antiviral adaptive immunity.

**Extended Data Fig. 2:**
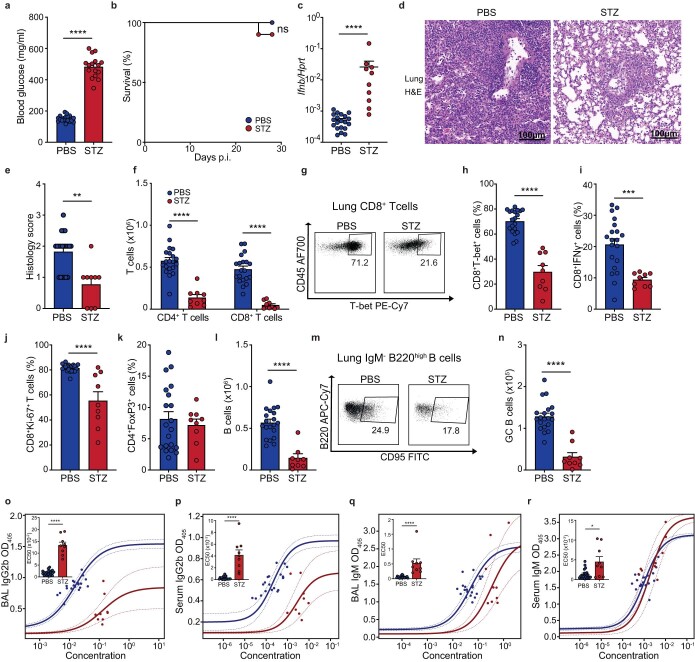
Diabetes associated susceptibility to respiratory viral infection is associated with broadly impaired adaptive immunity in streptozotocin-induced diabetes. **a**, Blood glucose, PBS (*n* = 20) or STZ (*n* = 16) administered mice, analyzed at 10 d.p.i., two-sided unpaired *t*-test. **b**, Survival, PBS (*n* = 10) or STZ (*n* = 10) administered mice, log-rank Mantel-Cox test. **c-r**, Mice infected with 50pfu PR8, administered PBS (*n* = 20) or STZ (*n* = 10 in **e**, *n* = 9 in all other panels), analyzed at 10 d.p.i. **c**, Lung *Ifnβ1*, two-sided Mann-Whitney *U*-test. **d-e**, Lung histology, **d**, representative sections, **e**, inflammatory score, two-sided Mann-Whitney *U*-test. **f**, Lung T cells, two-sided unpaired t-test. **g-h**, Flow cytometry,lung T-bet^+^CD8^+^ T cells, **g**, representative blots **h**, quantification, two-sided unpaired *t*-test. **i**, Lung IFNγ^+^CD8^+^ T cells, two-sided unpaired t-test. **j**, Lung Ki-67^+^CD8^+^ T cells, two-sided unpaired *t*-test. **k**, Lung FoxP3^+^CD4^+^ T cells, two-sided Mann-Whitney *U*-test. **l**, Lung B cells, two-sided Mann-Whitney *U*-test. **m-n**, Flow cytometry, GC B cells, **m**, representative blots, **n**, quantification, two-sided Mann-Whitney *U*-test. **o-p**, Virus specific IgG2b,**o**, BAL, **p**, serum, two-sided unpaired-test for EC50. **q**-**r**,Virus specific IgM, **q**, BAL, **r**, serum, two-sided Mann-Whitney test for EC50. All data mean+s.e.m.

**Extended Data Fig. 3:**
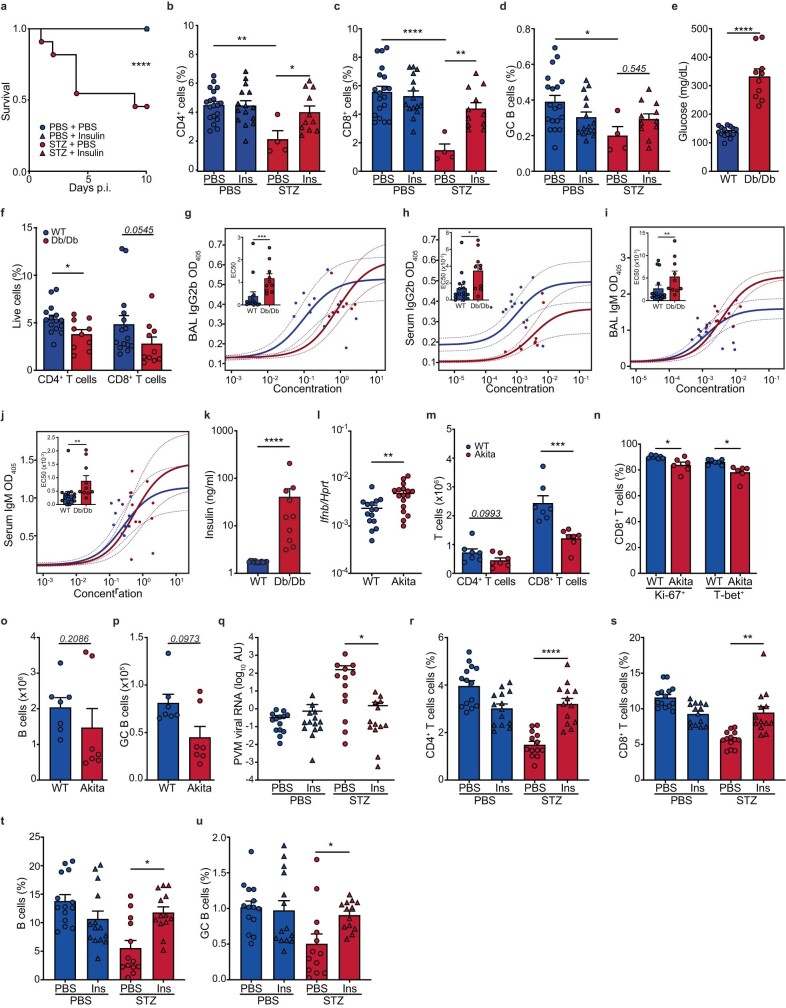
Normalization of glucose levels in diabetic animals with insulin restores adaptive antiviral immunity and attenuates susceptibility to respiratory viral infection. **a-d**, Mice infected with 50pfu PR8, administered STZ or PBS, treated with insulin/PBS: PBS+PBS (*n* = 19), PBS+Ins (*n* = 15), STZ+PBS (*n* = 4) STZ+Ins (*n* = 11), pooled data from 2 experiments. **a**, Survival curve (*n* = 11 mice/group), Log-rank Mantel Cox test. **b**, Lung CD4^+^ T cells, one-way ANOVA and Holm-Sidak correction. **c**, Lung CD8^+^ T cells, one-way ANOVA and Holm-Sidak correction. **d**, Lung GC B cells, Kruskall Wallis test and Dunn’s correction. **e**, Blood glucose, WT (*n* = 15) and Db/Db (*n* = 10) mice, two-sided unpaired *t*-test. **f-j**, WT (*n* = 14 for **g**, *n* = 15 for the other panels) and Db/Db (*n* = 10) mice infected with 50pfu PR8, pooled data from 3 experiments. **f**, Lung T cells, two-sided unpaired t-test for CD4^+^T cells and two-sided Mann-Whitney test for CD8^+^ T cells, respectively. **g-h**, Virus specific IgG2b, **g**, BAL, **h**, serum, two-sided Mann-Whitney test for EC50. **i-j,** IgM, **i**, BAL, **j**, serum, two-sided Mann-Whitney test for EC50. **k**, Blood insulin, WT (*n* = 15) and Db/Db (*n* = 10) mice, two-sided Mann-Whitney *U*-test. **l**, Lung *Ifnβ1* expression, WT (*n* = 15) and Akita (*n* = 17) mice infected with 50pfu PVM, two-sided Mann-Whitney *U*-test. **m-p**, WT (*n* = 7) and Akita mice (*n* = 6 in **n** and *n* = 7 in the other panels) infected with 50pfu PVM. **m**, Lung T cells, two-sided unpaired *t*-test. **n**, Lung Ki-67^+^CD8^+^ T cells and T-bet^+^CD8^+^ T cells, two-sided Mann Whitney *U*-test. **o**, Lung B cells, two-sided Mann Whitney *U*-test. **p**, Lung GC B cells, two-sided Mann Whitney *U*-test. **q-u**, Mice infected with 50pfu PVM, administered STZ or PBS, treated with insulin/PBS: PBS+PBS (*n* = 14), PBS+Ins (*n* = 14), STZ+PBS (*n* = 13), STZ+Ins (*n* = 13), pooled data from 2 experiments. **q**, Lung viral RNA, Kruskall Wallis test and Dunn’s correction. **r**, Lung CD4^+^ T cells, one-way ANOVA and Holm-Sidak correction. **s**, Lung CD8^+^ T cells, Kruskall Wallis test and Dunn’s correction. **t**, Lung B cells, Kruskall Wallis test and Dunn’s correction. **u**, Lung GC B cells, Kruskall Wallis test and Dunn’s correction. All data mean+s.e.m.

To generalize these findings, we used a model of hyperglycaemia induced by the administration of streptozotocin (STZ), which leads to rapid destruction of pancreatic insulin-producing β cells, driving acute hyperglycaemia (Extended Data Fig. 2a). Similar to the Akita model, infection of STZ-treated mice with IAV was associated with enhanced mortality compared with infected normoglycaemic animals (Fig. 1e) whereas no mortality enhancement was noted in non-infected STZ-treated animals (Extended Data Fig. 2b). Enhanced disease severity in STZ-treated mice was associated with higher type I IFN responses (Extended Data Fig. 2c), elevated viral titres (Fig. 1e), reduced pulmonary immune infiltration (Extended Data Fig. 2d,e) and severely impaired T and B cell antiviral responses, with no effect noted on numbers of lung T_reg_ cells (Fig. 1f and Extended Data Fig. 2f–r). Insulin replenishment to diabetic STZ-treated animals rescued viral clearance and survival (Fig. 1g and Extended Data Fig. 3a) and improved antiviral adaptive immunity (Fig. 1h and Extended Data Fig. 3b–d). In a third, type 2 diabetes model, in which hyperglycaemia spontaneously develops in leptin receptor-deficient (Db/Db) mice (Extended Data Fig. 3e), IAV infection induced lower survival (Fig. 1i), elevated viral titres (Fig. 1i) and impaired adaptive immunity in diabetic mice (Fig. 1j and Extended Data Fig. 3f–j) compared with infected non-diabetic littermate controls. Importantly, and in contrast to the above type 1 diabetes models, hyperglycaemia in Db/Db mice was accompanied by hyperinsulinaemia (Extended Data Fig. 3k), probably ruling out direct insulin impacts on IAV susceptibility. To further extend our findings beyond the context of influenza, we infected hyperglycaemic Akita mice with the respiratory pathogen mouse pneumonia virus (PVM) and noted increased mortality (Fig. 1k), elevated viral titres (Fig. 1k) and increased lung *Ifnb1* expression (Extended Data Fig. 3l), coupled with lower numbers of pulmonary T and B cells (Extended Data Fig. 3m–p). Similarly, PVM infection of STZ-treated mice led to reduced viral clearance (Extended Data Fig. 3q) and impaired adaptive immune responses, which were reversed using insulin supplementation (Extended Data Fig. 3r–u). Overall, our findings suggest that pulmonary viral infection in mice featuring types 1 and 2 diabetes-induced hyperglycaemia leads to a marked impairment of lung antiviral adaptive immunity, potentially driving delayed viral clearance and elevated mortality, which is reversible following insulin-mediated glucose lowering.

## Hyperglycaemia alters lung DC composition

**Fig. 2:**
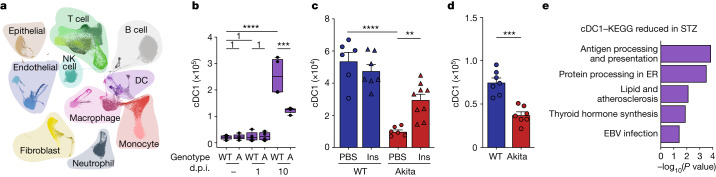
Diabetes alters lung DC in homeostasis and during respiratory viral infection. **a**,**b**, scRNA-seq of lungs during steady-state, 1 d.p.i. and 10 d.p.i. with 50 pfu PR8 (*n* = 4 per group). **a**, Uniform manifold approximation and projection (UMAP) of all cells. **b**, Lung cDC1, two-sided Wilcoxon test. Boxplots show 25th–75th percentiles, the 50th percentile denoted by a thicker line; whiskers show 1.5× interquartile range, or maximum or minimum if smaller than 1.5× interquartile range. Numeral (1) above the horizontal lines denotes the *P* value. A, Akita. **c**, Lung cDC1, WT and Akita mice infected with 50 pfu PR8 and treated with Ins/PBS: WT+PBS (*n* = 6), WT+Ins (*n* = 7), Akita+PBS (*n* = 6), Akita+Ins (*n* = 9), one-way analysis of variance (ANOVA) and Holm–Sidak correction. **d**, Lung cDC1, WT and Akita mice (*n* = 7 per group) infected with 50 pfu PVM, two-sided unpaired *t*-test. **e**, scRNA-seq of lung DC from PBS- (*n* = 4) and STZ-administered (*n* = 4) mice. Enriched Kyoto Encyclopedia of Genes and Genomes (KEGG) pathways in PBS- versus STZ-administered cDC1, *P* values corrected for multiple hypothesis testing with the g:SCS algorithm. All *P* values are indicated in Supplementary Table 1. ER, endoplasmic reticulum; EBV, Epstein–Barr virus.

To investigate the mechanisms driving pulmonary adaptive immune dysfunction and susceptibility to infection during hyperglycaemia, we next performed single-cell RNA sequencing (scRNA-seq) of lung cells from hyperglycaemic Akita mice and normoglycaemic WT littermate controls at both steady-state and two time points during acute IAV infection (1 and 10 d.p.i.). Collectively, we sequenced 154,545 single cells from 24 samples and identified 52 cell types/states across all conditions (Fig. 2a and Supplementary Information 4). During IAV infection we detected influenza transcripts in type 2 alveolar epithelial cells, ciliated epithelial cells and macrophages at both 1 and 10 d.p.i. but not in DC or lymphocytes (Supplementary Information 5a,b). As expected, we noted significant shifts in multiple pulmonary cell subset abundances and transcriptomic profiles during lung infection. Specifically, new subsets of macrophages, DC, natural killer (NK) cells, T cells, B cells, neutrophils and fibroblasts, among others, appeared during infection compared with the non-infected state (Fig. 2b and Supplementary Information 6). Acute IAV infection induced major global transcriptional response shifts in monocytes, alveolar and interstitial macrophages, DC, CD4^+^ T cells, CD8^+^ T cells, T Helper 17 (Th17) cells, T_reg_ cells, NK cells, basophils, type  2 alveolar epithelial cells, fibroblasts and endothelial cell subsets (Supplementary Information 7), and higher expression of chemokines including *Ccl2*, *Ccl7* and *Ccl8* (Supplementary Information 7b).

Differential expression profiling between normoglycaemic activated cell types and their corresponding steady-state cells identified hundreds of differentially expressed genes in each comparison (Supplementary Information 7a). Changes in cellular subsets and expression of secreted soluble factors, such as cytokines, were often similar across different cell types (Supplementary Information 7 and 8). Selection of genes featuring differential expression in at least five of ten comparisons showed many Gene Ontology terms related to interferon and immune response in shared upregulated genes, and also terms related to respiration and translation in shared downregulated genes (Supplementary Information 9). Respiration was prominently identified because mitochondrially encoded electron transport chain genes were lower in activated cells (Supplementary Information 10a). This was associated with a generally increased number of transcripts in all cell-activated types, suggesting that it is a relative rather than an absolute decrease in expression (Supplementary Information 10b). Moreover, promoters of commonly upregulated genes exhibited markedly over-represented interferon response factors binding sites, suggesting that interferons constitute key regulators of activation in this setting (Supplementary Information 10c).

**Extended Data Fig. 4:**
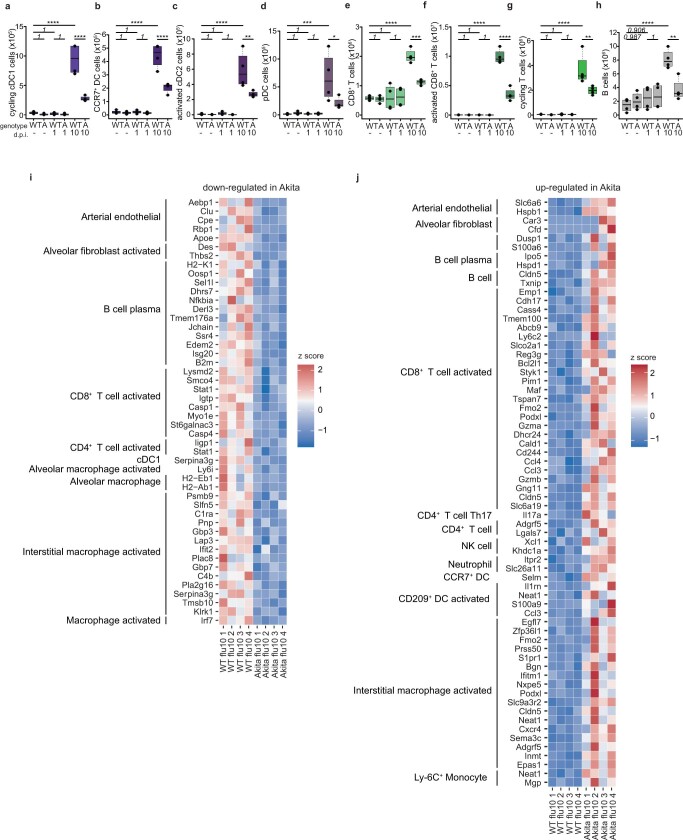
Diabetes is associated with transcriptomic changes in respiratory viral infection. **a-h**, scRNA-seq of lungs of WT and Akita mice (*n* = 4/group) collected during steady-state,1 d.p.i. and 10 d.p.i. with 50pfu PR8. Quantification of key cell subsets, two-sided Wilcoxon test. Boxplots show 25^th^ to 75^th^ percentiles, the 50^th^ percentile denoted by a thicker line; whiskers show 1.5× interquartile range max. or min. if smaller than 1.5× interquartile range. **i-j**, Heatmap with z-score of gene expression of differentially expressed genes between cells from WT and Akita, 10 d.p.i (*n* = 4/group). Differentially expressed genes identified by DESeq2 on pseudobulk counts in each population and adjusted p-value < 0.05. DESeq2 is based on Negative Binomial GLM fitting and Wald statistics with Benjamini and Hochberg procedure. **i**, downregulated, and **j**, upregulated genes in Akita mice. AT1, type 1 alveolar epithelial cell; AT2, type 2 alveolar epithelial cell.

We next explored cellular and genomic alterations driven by hyperglycaemia. During steady-state, no major differences in cell numbers were observed between normoglycaemic and hyperglycaemic mice (Fig. 2b and Supplementary Information 6). At 10 d.p.i., hyperglycaemic mice featured significantly fewer lung CD4^+^ and CD8^+^ T cells, including activated and cycling populations (Supplementary Information 6). In addition, hyperglycaemic mice harboured reduced numbers of lung follicular B cells (Supplementary Information 6), activated alveolar and interstitial macrophages and NK cells, compared with infected WT littermate controls (Supplementary Information 6). No major differences were noted in most non-haematopoietic cell types, except for a reduction in cycling fibroblasts (Supplementary Information 6). Importantly, infected hyperglycaemic animals evidenced significant reductions in the number of type 1 lung conventional DC (cDC1), including cDC1 cycling cells, CCR7^+^ migratory DC and activated cDC2, as well as plasmacytoid DC (pDC), compared with infected normoglycaemic littermate controls (Fig. 2b and Extended Data Fig. 4a–h). We did not observe a difference in CD64^+^ DC, which probably represent a mixture of monocyte-derived DC and recently described inflammatory cDC2 (ref. ^10^) (Supplementary Information 6). Differential expression with DESeq2 on pseudobulk counts of infected hyperglycaemic versus normoglycaemic mice showed multiple differentially expressed genes at 10 d.p.i. (Extended Data Fig. 4i,j). Genes higher in hyperglycaemic Akita were mostly related to chemotaxis (*Ccl3*, *Ccl4*, *Cxcr4*) and granzymes (*Gzma*, *Gzmb*), whereas those higher in normoglycaemic mice were regulated by interferons (*Stat1*, *Ifit2*, *Irf7*, *Gbp*) and involved in antigen presentation (*Psmb9*, *B2m*, *H2-Ab1*, *H2-K1*).

**Extended Data Fig. 5:**
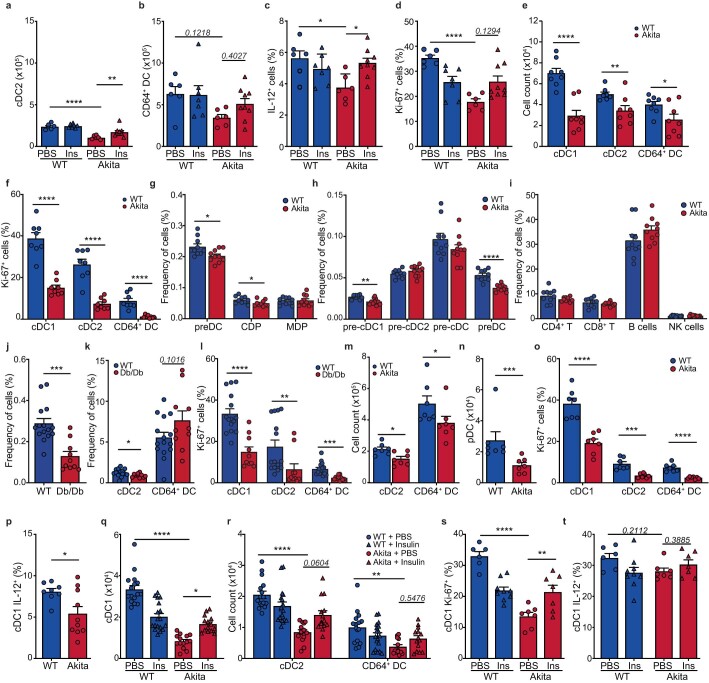
Hyperglycaemia dysregulates the lung DC compartment in infection and homeostasis. **a-d**, Mice infected with 50pfu PR8, treated with Ins/PBS: WT+PBS (*n* = 6), WT+Ins (*n* = 7), Akita+PBS (*n* = 6), Akita+Ins (*n* = 9). **a**-**c**, one-way ANOVA and Holm-Sidak correction, **d**, Kruskal Wallis test with Dunn’s correction. **a**, Lung cDC2. **b**, Lung CD64^+^ DC. **c**, Lung IL-12^+^cDC1. **d**, Lung Ki-67^+^cDC1. **e-f**, Naïve WT (*n* = 8) and Akita (*n* = 8) mice, two-sided unpaired *t*-test. **e**, Lung cDC1, cDC2 and CD64^+^ DC. **f**, Lung Ki-67^+^ DC. **g-i**, Naïve WT (*n* = 10) and Akita (*n* = 10) mice, two-sided unpaired *t*-test. **g**, BM DC precursors. **h**, BM Pre-DC subsets. **i**, Blood lymphocytes. **j-l**, WT (*n* = 15) and Db/Db (*n* = 10) mice infected with 50pfu PR8, pooled data from 3 experiments. **j**, Lung cDC1, two-sided Mann Whitney *U*-test. **k**, Lung cDC2 and CD64^+^ DC, two-sided unpaired *t*-test. **l**, Lung Ki-67^+^ DC, two-sided unpaired *t*-test for cDC1 and CD64^+^ DC, two-sided Mann Whitney *U*-test for cDC2. **m-p**, WT and Akita mice infected with 50pfu PVM (*n* = 7 per group in **m-o**, WT (*n* = 8) and Akita (*n* = 10) mice in **p**). **m**, Lung cDC2, two-sided unpaired *t*-test and CD64^+^ DC, Mann Whitney *U*-test. **n**, Lung pDC, two-sided Mann Whitney *U*-test. **o**, Lung Ki-67^+^ DC, two-sided unpaired *t*-test. **p**, Lung IL-12^+^cDC1, two-sided unpaired *t*-test. **q-r**, WT and Akita mice treated with insulin/PBS: WT+PBS (*n* = 16), WT+Ins (*n* = 18), Akita+PBS (*n* = 14) Akita+Ins (*n* = 14), pooled data from 2 experiments. **q**, Lung cDC1, Kruskal Wallis test with Dunn’s correction.**r**, Lung cDC2 and CD64^+^ DC, Kruskal Wallis test with Dunn’s correction. **s-t**, WT and Akita mice treated with insulin/PBS: WT+PBS (*n* = 6), WT+Ins (*n* = 9), Akita+PBS (*n* = 7) Akita+Ins (*n* = 7), one-way ANOVA and Holm-Sidak. **s**, Lung Ki-67^+^cDC1. **t**, Lung IL-12^+^cDC1. All data mean+s.e.m. CDP, Common dendritic cell progenitor; MDP, Macrophage dendritic cell progenitor.

**Extended Data Fig. 6:**
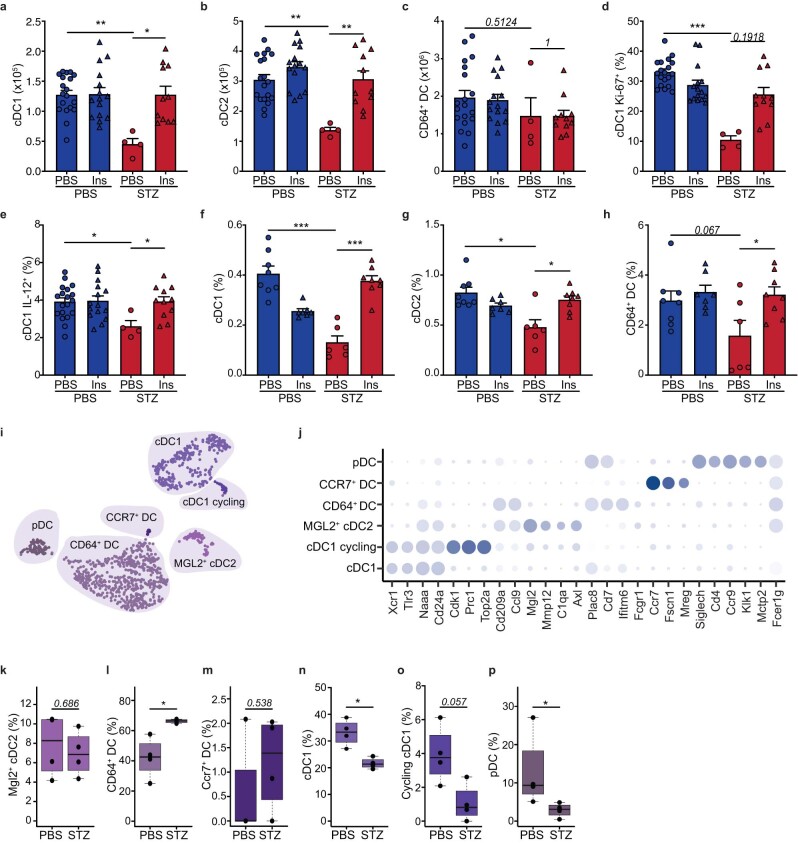
Insulin treatment improves outcomes of influenza infection. **a-e**, Mice infected with 50pfu PR8, administered STZ or PBS, treated with insulin/PBS: PBS+PBS (*n* = 19), PBS+Ins (*n* = 15), STZ+PBS (*n* = 4), STZ+Ins (*n* = 11), pooled data from 2 experiments. **a**, Lung cDC1, Kruskal Wallis test with Dunn’s correction. **b**, Lung cDC2, one-way ANOVA and Holm-Sidak. **c**, Lung CD64^+^ DC, Kruskal Wallis test with Dunn’s correction. **d**, Lung Ki-67^+^cDC1, Kruskal Wallis test with Dunn’s correction. **e**, Lung IL-12^+^cDC1, one-way ANOVA and Holm-Sidak. **f-h**, Mice infected with 50pfu PVM, administered STZ or PBS, treated with insulin/PBS: PBS+PBS (*n* = 8), PBS+Ins (*n* = 7), STZ+PBS (*n* = 6), STZ+Ins (*n* = 8). **f**, Lung cDC1, Kruskall Wallis test and Dunn’s correction. **g**, Lung cDC2, Kruskall Wallis test and Dunn’s correction. **h**, Lung CD64^+^ DC, one-way ANOVA and Holm-Sidak correction. **i-j**, scRNA-seq of lung DC from PBS or STZ administered WT mice (*n* = 4/group). **i**, UMAP showing all DC populations. **j**, Balloon plots with key population markers. Size of dots represents percentage of cells in which the gene was detected, color corresponds to mean scaled gene expression of all cells in a cluster. **k-p**, Quantification of all lung DC subsets not shown in Fig. 2, two-sided Wilcoxon test. Boxplots show 25^th^ to 75^th^ percentiles, the 50^th^ percentile denoted by a thicker line; whiskers show 1.5× interquartile range, or max. or min. if smaller than 1.5× interquartile range. All data mean+s.e.m.

Protein-level validation by flow cytometry-based analysis (Supplementary Information 11) of IAV-infected Akita mice at 10 d.p.i. corroborated that lung cDC1 (Fig. 2c) and, to a lesser extent, classical cDC2 and CD64^+^ DC, were reduced in lungs of infected hyperglycaemic mice compared with littermate controls (Extended Data Fig. 5a,b). Furthermore, we observed reduced expression of IL-12 and Ki-67^+^ in cDC1 from hyperglycaemic mice during IAV infection (Extended Data Fig. 5c,d). Interestingly, also at steady-state, lung DC subset numbers were significantly lower in hyperglycaemic Akita mice (Extended Data Fig. 5e and Supplementary Information 11) and were associated with a lower expression of Ki-67 (Extended Data Fig. 5f), suggesting that already in naïve mice lung DC may be developmentally impaired. Indeed, bone marrow analysis of naïve Akita mice showed a moderate reduction in pre-DC and common DC progenitors (Extended Data Fig. 5g), most notably pre-DC1 (Extended Data Fig. 5h). We did not observe an impact of hyperglycaemia on circulating lymphocytes (Extended Data Fig. 5i). Similar to type 1 diabetes models, Db/Db mice also exhibited a reduction in conventional lung DC but not CD64^+^ DC (Extended Data Fig. 5j,k) and lower frequency of Ki-67^+^ cells (Extended Data Fig. 5l), suggesting that it is not the absence of insulin, but rather hyperglycaemia, that probably drives lung DC aberrations. In corroboration of these influenza-related findings, lung DC in hyperglycaemic mice infected with PVM evidenced a depletion in lung cDC1, cDC2 and pDC (Fig. 2d and Extended Data Fig. 5m,n), coupled with fewer Ki-67^+^ DC and lower IL-12 expression in lung cDC1 (Extended Data Fig. 5o,p), compared with PVM-infected, normoglycaemic mice. Importantly, impairment in lung DC in virally infected Akita mice was rescued by insulin-driven lowering of hyperglycaemia (Fig. 2c and Extended Data Fig. 5a,b). Interestingly, even in non-infected, hyperglycaemic Akita mice, glucose normalization led to replenished numbers of pulmonary cDC1 and cDC2, and Ki-67^+^ cDC1 (Extended Data Fig. 5q–s), but not of steady-state expression of IL-12 (Extended Data Fig. 5t). In agreement, insulin-mediated correction of hyperglycaemia in IAV-infected, STZ-treated mice reversed the altered total lung DC composition (Extended Data Fig. 6a–c), cDC1 Ki-67^+^ cells (Extended Data Fig. 6d) and IL-12 expression (Extended Data Fig. 6e). Similarly, insulin supplementation reversed lung DC alterations in STZ-treated, PVM-infected mice (Extended Data Fig. 6f–h).

**Extended Data Fig. 7:**
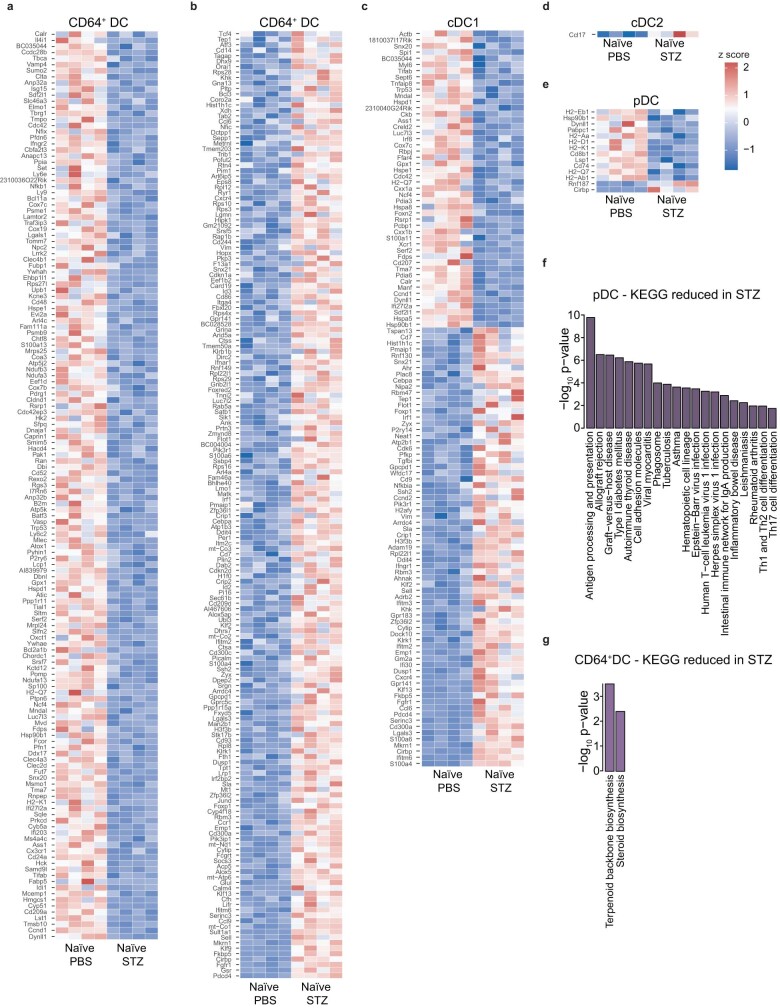
Hyperglycaemia dysregulates the lung DC compartment in steady-state in the streptozotocin-induced diabetes model. **a-g**, scRNA-seq of lung DC from PBS- or STZ-administered WT mice (*n* = 4/group). **a-e**, Heatmaps of differentially expressed genes with Z-score of tpm pseudobulk counts in **a**-**b**, CD64^+^ DC, **c**, cDC1, **d**, cDC2, and **e**, pDC. Differentially expressed genes identified by DESeq2 on pseudobulk counts in each population and adjusted p-value < 0.05. **f**, KEGG pathways reduced in pDC from STZ: bar plot showing KEGG pathway enrichment analysis with all differentially expressed genes higher in PBS controls than in STZ-administered mice, with gProfiler2 R package using standard settings; P values corrected for multiple hypothesis testing with g:SCS algorithm. **g**, KEGG pathways reduced in CD64^+^ DC, bar plot showing KEGG pathway enrichment analysis with all differentially expressed genes higher in PBS controls than in STZ-administered mice, with gProfiler2 R package using standard settings. P values corrected for multiple hypothesis testing using the g:SCS algorithm.

In corroberation of these findings, scRNA-seq of enriched lung DC from naïve STZ-treated mice and controls identified the same six DC populations that we observed in our previous non-enriched dataset, including pDC, cycling and non-cycling cDC1, cDC2, CD64^+^ DC DC and migratory CCR7^+^ DC (Extended Data Fig. 6i,j). Alike our previous findings, cDC1 were the most significantly impaired pulmonary immune cell population in hyperglycaemic mice, even at steady-state (Extended Data Fig. 6k–p). Differential expression analysis identified 320 differentially expressed genes in CD64^+^ DC, 115 in cDC1, one in cDC2 and 14 in pDC (Extended Data Fig. 7a–e). Across populations, DC from hyperglycaemic mice exhibited lower levels of genes related to antigen presentation (Fig. 2e and Extended Data Fig. 7f) and to terpenoid and steroid biosynthesis in CD64^+^ DC (Extended Data Fig. 7g). Overall, hyperglycaemia induced by different models was associated with major alterations of lung DC in steady-state and during viral infection, manifesting as compositional and gene expression aberrations in multiple lung DC subsets, most notably cDC1. DC impairment was rescued by insulin-based normalization of hyperglycaemia.

## Hyperglycaemia alters lung DC function

**Fig. 3:**
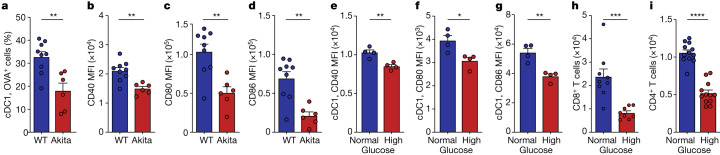
High glucose impairs lung DC function. **a**–**d**, WT (*n* = 9) and Akita (*n* = 6) mice infected with 50 pfu PR8 and intratracheally administered 100 μg of OVA-AF647, two-sided unpaired *t*-test. **a**, Lung dLN OVA^+^ cDC1. **b**, CD40 mean fluorescence intensity (MFI). **c**, CD80 MFI. **d**, CD86 MFI. **e**–**g**, WT lung cDC1 incubated with high (50 mM, *n* = 4) or normal (10 mM, *n* = 4) glucose.** e**, CD40 MFI, two-sided unpaired *t*-test. **f**, CD80 MFI, two-sided Mann–Whitney *U*-test. **g**, CD86 MFI, two-sided unpaired *U*-test. **h**, WT lung cDC1 incubated with high (50 mM, *n* = 8) or normal (10 mM, *n* = 9) glucose for 20 h, then co-incubated for 4 days with OT-I-CD8^+^ T cells in normal (10 mM) glucose. CD8^+^ T cells, two-sided unpaired *t*-test. **i**, WT lung cDC1 incubated with high (50 mM, *n* = 12) or normal (10mM, *n* = 12) glucose for 20 h, then co-incubated for 4 days with OT-II-CD4^+^ T cells in normal (10 mM) glucose. CD4^+^ T cells, two-sided unpaired *t*-test. All *P* values are indicated in Supplementary Table 1. All data mean+s.e.m.

**Extended Data Fig. 8:**
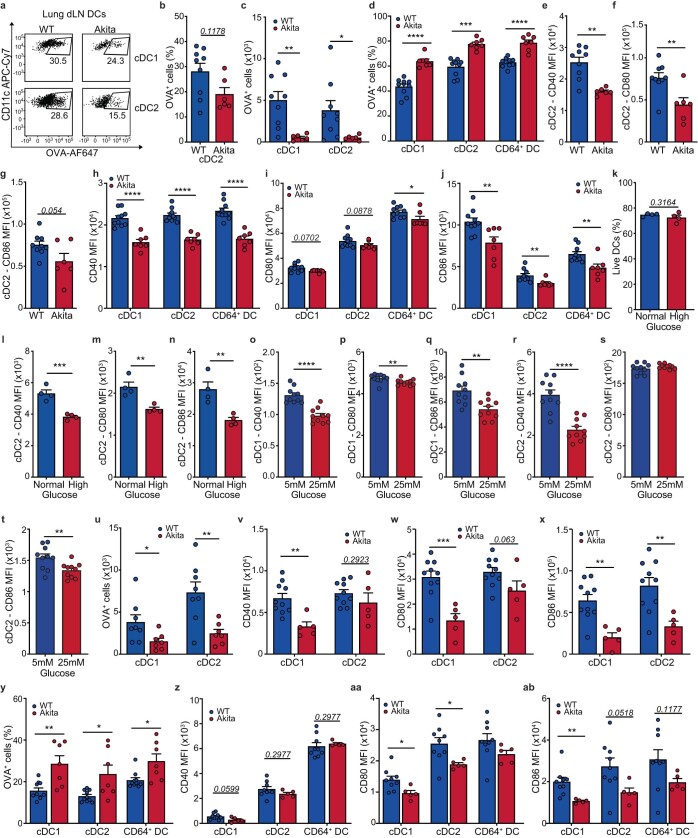
High glucose impairs lung DC immune functions. **a-d**, WT (*n* = 9) and Akita mice (*n* = 6) infected with 50 pfu PR8 and intratracheally administered 100μg OVA-AF647. **a**, Representative flow cytometry, lung dLN OVA^+^DC. **b**, Percentage of lung dLN OVA^+^cDC2, two-sided unpaired *t*-test. **c**, Number of lung dLN OVA^+^DC, two-sided unpaired *t*-test. **d**, Frequency of lung OVA^+^DC, WT (*n* = 10) and Akita (*n* = 7) mice, cDC1 and CD64^+^ DC, two-sided unpaired *t*-test, cDC2, Mann Whitney *U*-test. **e-g**, Lung dLN cDC2 MFI, WT (*n* = 9) and Akita (*n* = 6) mice, two-sided unpaired t-test. **e**, CD40. **f**, CD80. **g**, CD86. **h-j**, Lung DC MFI, WT (*n* = 10) and Akita (*n* = 7) mice. **h**, CD40, two-sided Mann Whitney *U*-test. **i**, CD80, two-sided unpaired *t*-test. **j**, CD86, two-sided unpaired *t*-test. **k**, WT Lung DC incubated with high (50mM, *n* = 4) or normal (10mM, *n* = 4) glucose, cell viability, two-sided unpaired *t*-test. **l-n**, WT Lung cDC2 incubated with high (50mM, n = *4*) or normal (10mM, *n* = 4) glucose, MFI, two-sided unpaired *t*-test. **l**, CD40. **m**, CD80. **n**, CD86. **o-t**, WT Lung DC incubated with 25 mM (*n* = 10) or 5 mM (*n* = 10) glucose, two-sided Mann Whitney U-test for **p**, two-sided unpaired *t*-test for all other panels. **o**, cDC1 CD40. **p**, cDC1 CD80. **q**, cDC1 CD86. **r**, cDC2 CD40. **s**, cDC2 CD80. **t**, cDC2 CD86. **u-ab**, WT and Akita mice intratracheally administered 100μg HDM + 100 μg OVA-AF647.**u**, Lung dLN OVA^+^DC, WT (*n* = 8) and Akita (*n* = 7) mice, two-sided unpaired *t*-test. **v-x**, Lung dLN DC MFI, WT (*n* = 10) and Akita (*n* = 5) mice, two-sided unpaired t-test. **v**, CD40. **w**, CD80. **x**, CD86. **y**, Lung OVA^+^DC frequency, WT (*n* = 10) and Akita (*n* = 7) mice, two-sided Mann Whitney *U*-test. **z-ab**, Lung DC MFI, WT (*n* = 9) and Akita (*n* = 5) mice. **z**, CD40, two-sided unpaired *t*-test. **aa**, CD80, two-sided Mann Whitney U-test. **ab**, CD86, two-sided Mann Whitney *U*-test. All data mean+s.e.m.

We next studied the functional consequences of hyperglycaemia on lung DC function. During an encounter with a pathogen, lung DC engulf and transport antigens to the lung-draining lymph nodes (dLN), where they prime naïve T cells. Indeed, simultaneous injection of the fluorescent antigen ovalbumin (OVA)-AF647 into IAV-infected Akita hyperglycaemic mice and normoglycaemic littermate controls showed that the frequency and number of DC carrying OVA in lung dLN were reduced in hyperglycaemic mice (Fig. 3a and Extended Data Fig. 8a–c). By contrast, OVA uptake was enhanced in lung DC that had not migrated to lung dLN (Extended Data Fig. 8d). Furthermore, expression levels of the key lung DC costimulatory molecules CD40, CD80 and CD86, which are required for effective antigen presentation to T cells, were lower in total dLN DC in infected hyperglycaemic animals (Fig. 3b–d and Extended Data Fig. 8e–g) and, to a lesser extent, also in non-migratory lung DC (Extended Data Fig. 8h–j) compared with infected normoglycaemic controls. To further demonstrate that these key lung DC functions are directly impacted by hyperglycaemia, we isolated lung DC from naïve WT animals and incubated them with either high (50 mM) or normal (10 mM) levels of glucose (Supplementary Information 12). Although high glucose had no impact on cell viability (Extended Data Fig. 8k), it directly reduced expression of costimulatory molecules in cDC1 and cDC2 compared with normal glucose controls (Fig. 3e–g and Extended Data Fig. 8l–n). Exposure to intermediate concentrations of excessive glucose (25 mM) yielded similar effects (Extended Data Fig. 8o–t).

**Extended Data Fig. 9:**
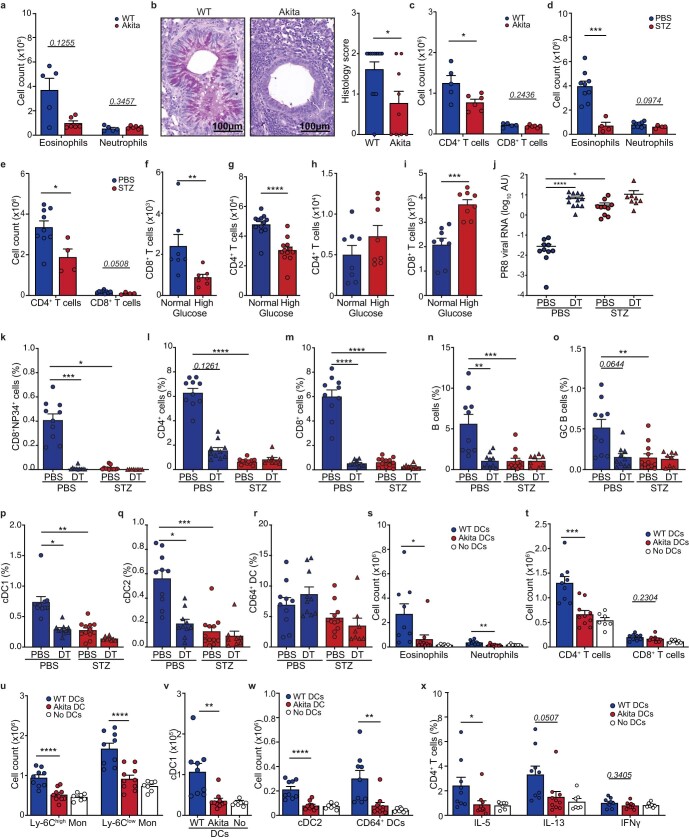
Hyperglycaemia protects from allergic lung inflammation. **a-c**, WT and Akita mice intratracheally administered 10μg HDM at day 0, 7-11, analyzed at day 14. **a**, Lung eosinophils and neutrophils, WT (*n* = 5) and Akita (*n* = 6) mice, two-sided Mann Whitney *U*-test. **b**, Lung PAS staining and mucus quantification,WT (*n* = 13) and Akita (*n* = 9) mice, two-sided Mann Whitney *U*-test. **c**, Lung CD4^+^ T cells and CD8^+^ T cells, WT (*n* = 5) and Akita (*n* = 6) mice, two-sided unpaired *t*-test. **d-e**, STZ (*n* = 4)- or PBS (*n* = 9)-pretreated mice intratracheally received 10μg HDM at day 0, 7-11, analyzed on day 14. **d**, Lung eosinophils and neutrophils. **e**, Lung CD4^+^ T cells and CD8^+^ T cells. **f**, WT lung cDC2 incubated in vitro with high (50mM, *n* = 7) or normal (10mM, *n* = 7) glucose for 20 h, then co-cultured for 4 days with OT-I-CD8^+^ T cells in normal (10mM) glucose. CD8^+^T cells, two-sided Mann-Whitney *U*-test. **g**, WT lung cDC2 incubated in vitro with high (50mM, *n* = 12) or normal (10mM, *n* = 12) glucose for 20 h, then co-cultured for 4 days with OT-II-CD4^+^ T cells in normal (10mM) glucose. CD4^+^ T cells, two-sided unpaired *t*-test. **h-i**, T cell activation by anti-CD3 and anti-CD28 in the presence of high (50mM, *n* = 8) or normal (10mM, *n* = 8) glucose, analyzed at day 4. **h**, CD8^+^ T cells, two-sided unpaired *t*-test. **i**, CD4^+^ T cells, two-sided Mann-Whitney *U*-test. **j-r**, WT mice sublethally irradiated and transplanted with a Zbtb46-DTR bone marrow, followed, after 8 weeks, by administration of STZ or PBS. 2 weeks later, mice infected with 50pfu PR8 and treated, every other day, with PBS or DT: PBS+PBS (*n* = 10), PBS+DT (*n* = 10), STZ+PBS (*n* = 12) and STZ+DT (*n* = 8). **j**, Lung PR8 viral titer, Kruskall Wallis test and Dunn’s correction. **k**, Lung virus-specific NP34 Tetramer^+^CD8^+^ T cells, Kruskall Wallis test and Dunn’s correction. **l**, Lung CD4^+^ T cells, Kruskall Wallis test and Dunn’s correction. **m**, Lung CD8^+^ T cells, one-way ANOVA and Holm-Sidak correction. **n**, Lung B cells, Kruskall Wallis test and Dunn’s correction. **o**, Lung GC B cells, Kruskall Wallis test and Dunn’s correction. **p**, Lung cDC1, Kruskall Wallis test and Dunn’s correction. **q**, Lung cDC2, Kruskall Wallis test and Dunn’s correction. **r**, Lung CD64^+^ DC, Kruskall Wallis test and Dunn’s correction. **s-x**, WT and Akita mice intratracheally administered 100μg HDM, followed, 24 h later, by DC sorting and transfer to WT recipients (receiving WT DC (n = 9), Akita DC (n = 10), or no DC (n = 7)). Recipient mice intratracheally challenged by 10μg HDM daily from day 7-11, analyzed at day 14. **s**, Lung eosinophils and neutrophils, Kruskall Wallis test and Dunn’s correction. **t**, Lung T cells, one-way ANOVA and Holm-Sidak correction. **u**, Lung Ly-6C^high^ and Ly-6C^low^monocytes, one-way ANOVA and Holm-Sidak correction. **v**, Lung cDC1, Kruskal Wallis test with Dunn’s correction. **w**, Lung cDC2, one-way ANOVA and Holm-Sidak correction and CD64^+^ DC, Kruskal Wallis test with Dunn’s correction. **x**, CD4^+^ T cell IL-5 and IL-13 expression, Kruskall Wallis test and Dunn’s correction, and CD4^+^ T cell IFNγ expression, one-way ANOVA and Holm-Sidak correction. All data mean+s.e.m. PAS, Periodic acid-schiff; DT, diphtheria toxin.

To generalize these hyperglycaemia-induced functional lung DC impairments in antigen transport and costimulation beyond the pulmonary viral infection context we used the house dust mite (HDM) model, a common model of allergic inflammation mimicking asthma, which critically involves cDC activation and adaptive immune instruction^11^. Similar to IAV infection, administration of HDM extract together with OVA-AF647 to hyperglycaemic mice led to unaffected antigen uptake and reduced antigen transport, coupled with lowered expression of several costimulatory molecules in both migratory and lung-resident DC as compared with non-hyperglycaemic littermate controls (Extended Data Fig. 8u-ab). Furthermore, HDM challenge of hyperglycaemic mice was associated with protection from key asthma hallmarks, including eosinophilia and excessive mucus production, in both the Akita and STZ hyperglycaemia models compared with non-hyperglycaemic controls (Extended Data Fig. 9a–e).

To evaluate the functional consequences of high glucose on DC-mediated T cell priming, WT lung DC from normoglycaemic animals were incubated in vitro with high (50 mM) or normal (10 mM) glucose and OVA for 20 h, followed by co-incubation with OT-I CD8^+^ T cells in normal (10 mM) glucose medium (Supplementary Information 12b). Following 4 days of coculture, DC previously incubated in high glucose exhibited an impaired capacity to induce T cell expansion compared with those in normal glucose (Fig. 3h and Extended Data Fig. 9f), suggesting that high glucose had directly suppressed their capacity to activate T cells. Similarly, incubation of DC in high glucose impaired their capacity to activate OT-II CD4^+^ T cells (Fig. 3i and Extended Data Fig. 9g), whereas high glucose had no detrimental effect on direct anti-CD3- and anti-CD28-mediated T cell activation (Extended Data Fig. 9h,i). To corroborate these findings, we generated Zbtb46-DTR Bone Marrow (BM) chimeric mice in which a specific depletion of DC is achieved following diphtheria toxin treatment. In this model, DC-depleted, IAV-infected mice exhibited impaired antiviral immunity (Extended Data Fig. 9j–r) similar in magnitude and not further exacerbated by the co-induction of STZ-mediated hyperglycaemia.

**Extended Data Fig. 10:**
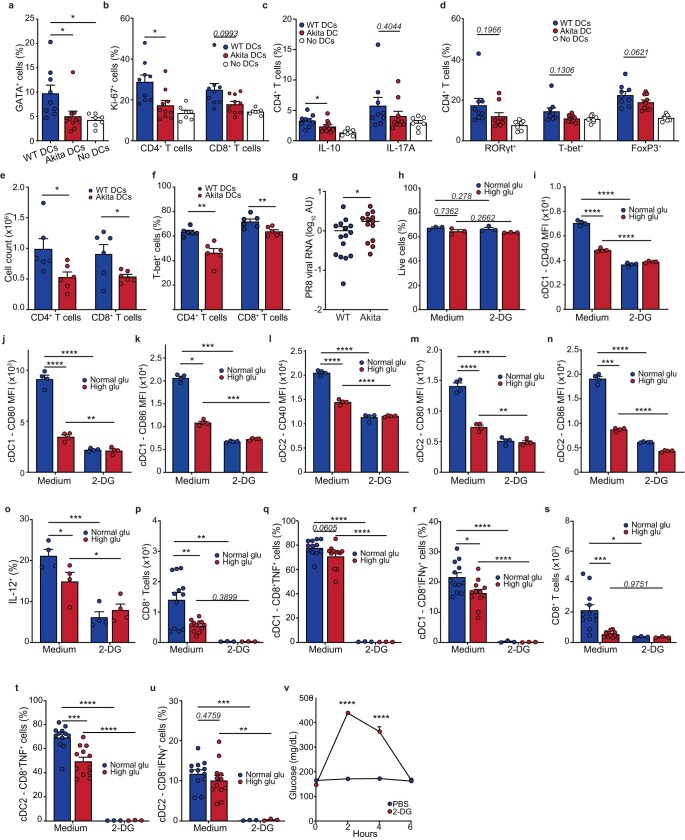
Lung DC from diabetic animals induce an attenuated T cell response. **a-h**, WT and Akita mice intratracheally administered 100μg HDM, followed, 24h later, by DC sorting and transfer to WT recipients (receiving WT DC (*n* = 9), Akita DC (*n* = 10), or No DC (*n* = 7)). Recipient mice intratracheally challenged by 10μg HDM daily from day 7-11, analyzed at day 14. **a**, GATA3^+^CD4^+^ T cells, Kruskall Wallis test and Dunn’s correction. **b**, Ki-67^+^ T cells, Kruskall Wallis test and Dunn’s correction. **c**, Frequency of lung IL-10^+^CD4^+^ T cells, one-way ANOVA and Holm-Sidak correction, and IL-17A^+^CD4^+^ T cells, Kruskal Wallis test with Dunn’s correction. **d**, Frequency of lung RORγt^+^CD4^+^ T cells, T-bet^+^CD4^+^ T cells, Kruskal Wallis test with Dunn’s correction, and FoxP3^+^CD4^+^ T cells, one-way ANOVA and Holm-Sidak correction. **e-g**, WT and Akita mice intratracheally administered UV-inactivated PR8, followed 20 h later, by DC sorting and trasnfer to WT recipients (receiving WT DC (*n* = 6), WT+Akita DC (*n* = 6)). 10 days later, recipients infected with 500pfu PR8. **e**, Lung CD4^+^ T cells and CD8^+^ T cells, two-sided unpaired t-test. **f**, Frequency of T-bet^+^ cells, unpaired t-test. **g**, PR8 viral RNA, Mann Whitney test, WT+WT DC (*n* = 15), WT+Akita DC (*n* = 13), pooled from 2 experiments. **h-o**, WT lung DC incubated with high (50mM)/normal (10mM) glucose and 2-DG/vehicle for 20h, two-way ANOVA and Holm-Sidak correction. **h**, lung DC viability (*n* = 3/group). **i-k**, cDC1 (*n* = 4/group). **i**, CD40 MFI. **j**, CD80 MFI. **k**, CD86 MFI. **l-n**, cDC2 (*n* = 4/group). **l**, CD40 MFI. **m**, CD80 MFI. **n**, CD86 MFI. **o**, IL-12^+^ cells (*n* = 4/group). **p-r**, WT lung cDC1 incubated for 20 h with high (50mM, n = 12)/normal (10mM, *n* = 12) glucose, and high (50mM) glucose+2-DG (*n* = 3)/normal (10mM) glucose+2-DG (*n* = 3), then co-cultured with OT-I-CD8^+^ T cells for 4 days in normal (10mM) glucose (in the absence of 2-DG), two-way ANOVA and Holm-Sidak correction. **p**, CD8^+^ T cells. **q**, TNF^+^ cells. **r**, IFNγ^+^ cells. **s-u**, WT Lung cDC2 incubated for 20 h with high (50mM, *n* = 12)/ normal (10mM, *n* = 12) glucose, and high (50mM) glucose+2-DG (*n* = 3)/normal (10mM) glucose+2-DG (*n* = 3), then co-cultured with OT-I-CD8^+^ T cells for 4 days in normal (10mM) glucose (in the absence of 2-DG), two-way ANOVA and Holm-Sidak correction. **s**, CD8^+^ T cells. **t**, TNF^+^ cells. **u**, IFNγ^+^ cells. **v**, Mice intraperitoneally administered 250 mg/kg 2-DG (*n* = 5) or PBS (*n* = 5). Blood glucose measured at indicated time-points, two-sided unpaired *t*-test for each time-point. All data mean+s.e.m.

To evaluate whether hyperglycaemia directly impairs the ability of lung DC to induce adaptive immunity in vivo, we intratracheally immunized hyperglycaemic Akita mice and normoglycaemic littermate controls with the innocuous antigen HDM, followed by isolation of lung DC 1 day after treatment and their transfer to naïve WT normoglycaemic recipients. Subsequent HDM challenge in recipients showed that lung DC transferred from hyperglycaemic animals poorly induced lung inflammation, leading to lower infiltration of granulocytes, T cells and monocytes, as compared with lung DC transferred from normoglycaemic donors (Extended Data Fig. 9s–w and Supplementary Information 13). Consequently, recipients of lung DC from hyperglycaemic animals developed marked alterations in their pulmonary adaptive immune response, manifesting as a lower frequency of Ki-67^+^CD8^+^ T cells, CD4^+^ T cells, and Th2 cytokine-producing cells including GATA3^+^CD4^+^ T cells, as compared with recipients of DC from normoglycaemic donors (Extended Data Figs. 9x and 10a–d). To validate these findings in the context of a respiratory viral infection, while avoiding concomitant transfer of live virions with isolated donor DC, we administered WT and diabetic Akita mice with ultraviolet-inactivated PR8 influenza virus, followed, 24 h later, by transfer of lung DC to naïve WT normoglycaemic recipients. Indeed, subsequent challenge of recipient mice with a high viral dose showed impaired capacity of DC from diabetic animals to induce a CD4^+^ and CD8^+^ T cell response (Extended Data Fig. 10e,f), which was associated with an elevated viral titre compared with animals receiving DC from normoglycaemic donors (Extended Data Fig. 10g). Collectively, these results suggest that hyperglycaemia drives functional defects in lung DC, resulting in major downstream effects on adaptive lung immune activation and function in both infectious and non-infectious inflammatory settings.

## Hyperglycaemia alters lung DC metabolism

**Extended Data Fig. 11:**
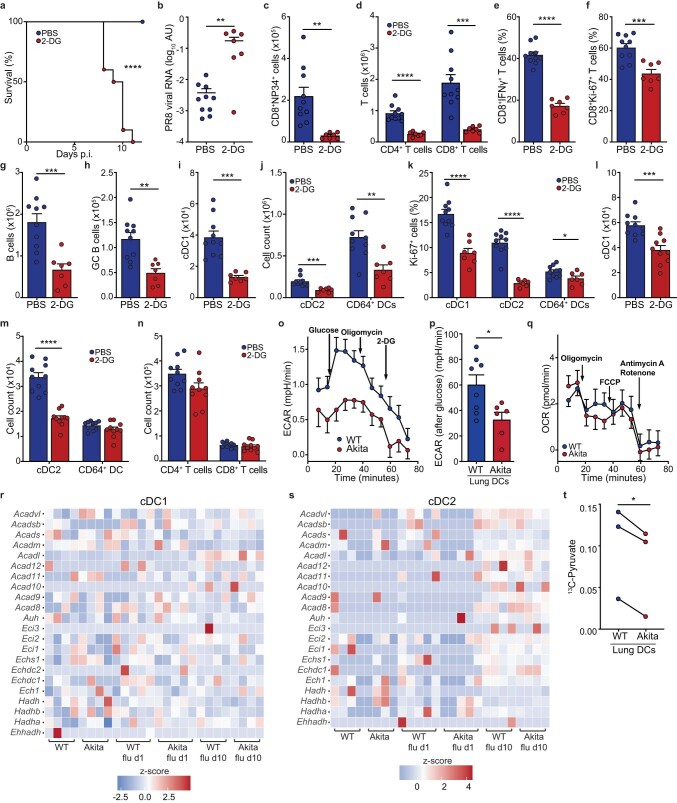
Diabetes does not impact lung DC lipid metabolism or glucose uptake. **a**, Mice infected with 200pfu PR8 and intraperitoneally administered 250 mg/kg 2-DG (*n* = 10) or PBS (*n* = 10) daily. Survival, log-rank Mantel-Cox test. **b-k**, Mice infected with 50pfu PR8 and intraperitoneally administered 250 mg/kg 2-DG (*n* = 7) or PBS (*n* = 10) daily. **b**, Lung PR8 viral RNA, two-sided Mann-Whitney *U*-test. **c**, Lung virus-specific NP34 Tetramer^+^CD8^+^ T cells, two-sided unpaired *t*-test. **d**, Lung CD8^+^ T cells and CD4^+^ T cells, two-sided unpaired t-test. **e**, Frequency of IFNγ^+^CD8^+^ T cells, two-sided unpaired *t*-test. **f**, Lung Ki-67^+^CD8^+^ T cells, two-sided unpaired *t*-test. **g**, B cells,two-sided unpaired t-test. **h**, GC B cells, two-sided unpaired *t*-test. **i**, Lung cDC1, two-sided unpaired t-test. **j**, Lung cDC2, two-sided Mann-Whitney U-test and CD64^+^ DC, two-sided unpaired t-test. **k**, Lung Ki-67^+^cDC1, Ki-67^+^cDC2 and Ki-67^+^CD64^+^ DC, two-sided unpaired *t*-test. **l-n**, Mice intraperitoneally administered 250 mg/KG 2-DG (*n* = 10) or PBS (*n* = 10) daily for 10 days, two-sided unpaired t-test. **l**, Lung cDC1. **m**, Lung cDC2 and Lung CD64^+^ DC. **n**, Lung T cells. **o-p**, Seahorse ECAR analysis of lung DC, WT (*n* = 8) and Akita (*n* = 6) mice, two-sided unpaired t-test. **q**, Seahorse OCR of lung DC, WT (*n* = 12) and Akita (*n* = 8) mice, AUC, two-sided Mann-Whitney *U*-test. **r-s**, scRNA-seq of lung DC of genes involved in beta-oxidation. Heatmap shows z-scores of mean scaled and log normalized gene expression of cells in different DC. **t**, Lung DC incubated with 11 mM ^13^C-glucose. Intracellular pyruvate measured after 6 h (isotopologue fractional enrichment), 3 pooled experiments, two-sided paired t-test. All data mean+s.e.m. AUC, area under the curve; OCR, oxygen consumption rate.

We next sought to elucidate the underlying mechanisms orchestrating hyperglycaemia-induced lung DC functional impairment. Given the emerging links between glucose metabolism and immune function in immune cells such as macrophages^9^ or T cells^12^, we hypothesized that high glucose levels could lead to alterations in lung DC glycolysis and downstream metabolic circuits which, in turn, could affect their immune function. Several important hints suggested such a link. First, murine WT lung DC expressed the enzyme machinery necessary for glycolysis (Supplementary Information 14a). Second, our single-cell transcriptomic analysis demonstrated marked changes in expression of genes involved in glucose metabolism in lung DC of hyperglycaemic animals, including increased expression of phosphofructokinase (*Pfkp*), fructokinase (*Khk*), pyruvate dehydrogenase (*Pdk1*) and pyruvate transporter (*Mpc2*), and decreased expression of hexokinase (*Hk2*), in different DC subpopulations (Supplementary Information 14b–f). Third, inhibition of glycolysis in normoglycaemic or hyperglycaemic DC by in vitro incubation with the glycolysis inhibitor 2-deoxyglucose (2-DG) at non-toxic concentrations (Extended Data Fig. 10h) induced impaired expression of costimulatory molecules (Extended Data Fig. 10i–n), IL-12 (Extended Data Fig. 10o), T cell induction and cytokine production (Extended Data Fig. 10p–u). In vivo, daily 2-DG treatment of IAV-infected mice led to transient hyperglycaemia after each injection (Extended Data Fig. 10v), impaired survival (Extended Data Fig. 11a), delayed viral clearance (Extended Data Fig. 11b), impaired adaptive immunity (Extended Data Fig. 11c–h) and reduced lung cDC1 (Extended Data Fig. 11i–k). 2-DG-treated naïve mice demonstrated a similar phenomenon in DC (Extended Data Fig. 11l,m) but not in T cells (Extended Data Fig. 11n).

**Fig. 4:**
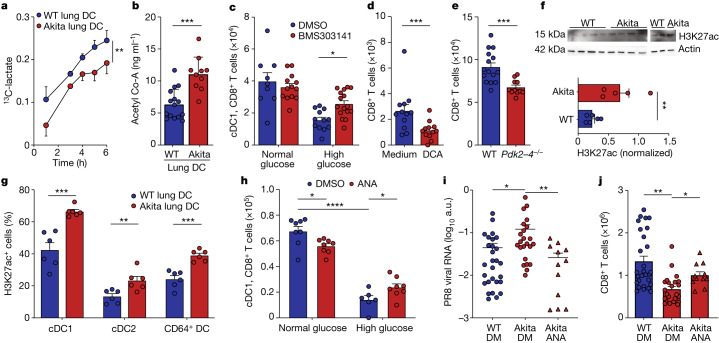
Hyperglycaemia dysregulates lung DC metabolism and acetylation. **a**, WT (*n* = 4) and Akita (*n* = 4) lung DC incubated with 11 mM ^13^C-glucose. Supernatant ^13^C-lactate, area under the curve, two-sided unpaired *t*-test. **b**, WT (*n* = 15) and Akita (*n* = 10) lung DC acetyl-CoA, two-sided unpaired *t*-test. **c**, WT lung cDC1 incubated with high (50 mM, *n* = 12)/normal (10 mM, *n* = 9) glucose or with BMS303141 in high (50 mM, *n* = 16)/normal (10 mM, *n* = 13) glucose, then co-cultured for 4 days with OT-I-CD8^+^ T cells in normal (10 mM) glucose (in the absence of inhibitor). CD8^+^ T cells, two-way ANOVA with Holm–Sidak correction. **d**, WT lung cDC1 incubated for 20 h with 10 mM dichloroacetate (DCA) (*n* = 12) or medium (*n* = 12), then co-cultured for 4 days with OT-I-CD8^+^ T cells in normal (10 mM) glucose (in the absence of inhibitor). CD8^+^ T cells, two-sided Mann–Whitney *U*-test. **e**, OT-I-CD8^+^ T cells incubated for 4 days with lung WT cDC1 (*n* = 15) or *Pdk2–4*^*−/−*^ cDC1 (*n* = 11). CD8^+^ T cells, two-sided unpaired *t*-test. **f**, H3K27ac immunoblot of lung DC from WT (*n* = 6) and Akita (*n* = 5) mice, two-sided unpaired *t*-test. **g**, Fluorescent activated cell sorting of lung WT (*n* = 6) and Akita (*n* = 6) H3K27ac^+^ DC, two-sided unpaired *t*-test. **h**, WT lung cDC1 incubated for 20 h with high (50 mM, *n* = 6)/normal (10 mM, *n* = 8) glucose, or with 10 mM ANA in high (50 mM, *n* = 8)/normal (10 mM, *n* = 8) glucose, then co-cultured for 4 days with OT-I-CD8^+^ T cells and normal (10 mM) glucose (in the absence of inhibitor). CD8^+^ T cells, two-way ANOVA with Holm–Sidak correction. **i**,**j**, WT and Akita mice intraperitoneally administered 5 mg kg^−1^ ANA or vehicle (dimethyl sulfoxide, DM) for 5 days, followed by intratracheal administration of 50 μg of OVA + 50 μg poly I:C. 24 h later, lung DC were sorted and transferred to naïve WT mice: WT+DM DC (*n* = 28), Akita+DM DC (*n* = 23), Akita+ANA DC (*n* = 12 for **i**, *n* = 11 for **j**). 10 days later, recipient mice were administered 500 pfu PR8-OVA (SIINFEKL), analyzed at day 7, Kruskal–Wallis test with Dunn’s correction. **i**, Lung OVA-PR8 viral RNA. **j**, Lung CD8^**+**^ T cells. All *P* values are indicated in Supplementary Table 1. All data mean+s.e.m.

**Extended Data Fig. 12:**
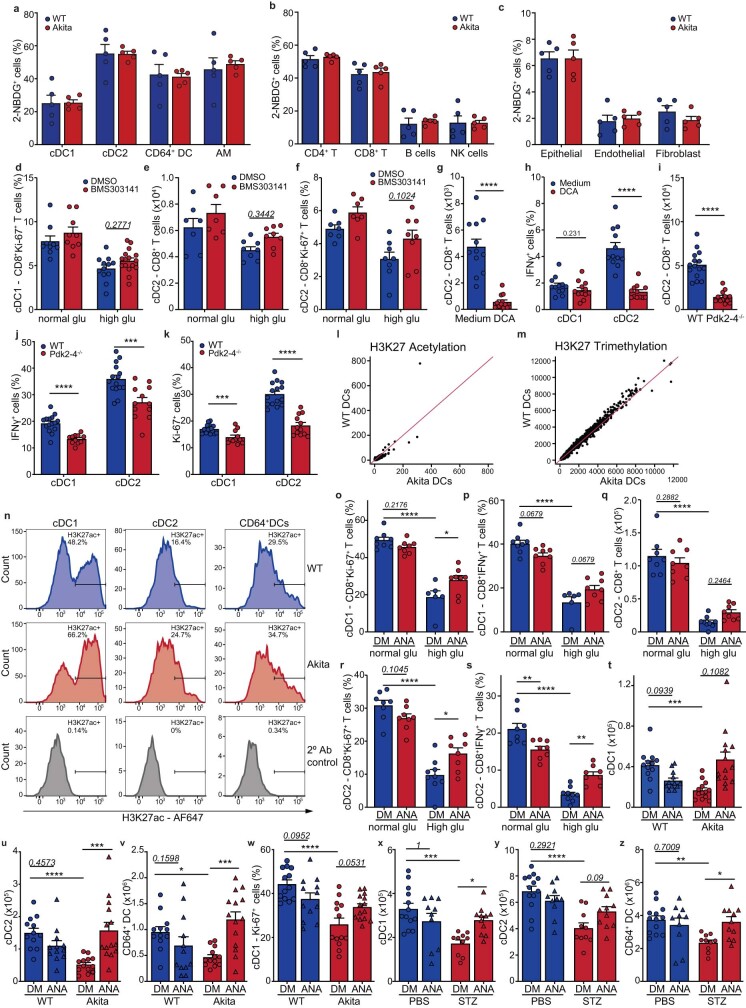
Diabetes-induced hyperacetylation impairs lung DC. **a-c**, WT (*n* = 5) and Akita (*n* = 5) mice intravenously administered 5 mM 2-NBDG. Frequency of 2-NBDG^+^ cells within immune cell compartments, two-sided unpaired *t*-test. **d**, WT lung cDC1 incubated with high (50mM) or normal (10mM) glucose and BMS303141 for 20h, then co-cultured for 4 days with OT-I-CD8^+^ T cells in normal (10mM) glucose (in the absence of BMS303141): High glucose+DMSO (*n *= 12), normal glucose+DMSO (*n* = 9), high glucose+BMS303141 (n = 16), normal glucose+BMS303141 (*n* = 9). Frequency of Ki-67^+^CD8^+^ T cells, two-way ANOVA and Holm-Sidak correction. **e-f**, WT lung cDC2 incubated with high (50mM) or normal (10mM) glucose and BMS303141 for 20h, then co-cultured for 4 days with OT-I CD8^+^ T cells in normal (10mM) glucose (in the absence of BMS303141): High glucose+DMSO (*n* = 8), normal glucose+DMSO (*n* = 7), high glucose+BMS303141 (*n* = 8), normal glucose+BMS303141 (*n* = 8), two-way ANOVA and Holm-Sidak correction. **e**, CD8^+^ T cells. **f**, Ki-67^+^CD8^+^ T cells. **g-h**, WT lung DC incubated with 10 mM DCA (*n* = 12) or vehicle (*n* = 12) for 20h, then co-cultured for 4 days with OT-I-CD8^+^ T cells in normal (10mM) glucose. **g**, CD8^+^ T cells, co-cultured with cDC2, two-sided Mann-Whitney *U*-test. **h**, IFNγ^+^CD8^+^ T cells, co-cultured with cDC1, two-sided unpaired t-test, IFNγ^+^CD8^+^ T cells, co-cultured with cDC2, two-sided Mann-Whitney *U*-test. **i-k**, OT-I-CD8^+^ T cells, co-cultured for 4 days with lung WT cDC1 (*n* = 15), WT cDC2 (*n* = 15), *Pdk2-4*^*−/−*^ cDC1 (*n* = 11) or *Pdk2-4*^*−/−*^ cDC2 (*n* = 12), two-sided unpaired *t*-test. **i**, CD8^+^ T cells, co-cultured with cDC2. **j**, IFNγ^+^CD8^+^T cells, and **k**, Ki-67^+^CD8^+^ T cells, cocultured with cDC1 and cDC2. **l-m**, CUT&Tag chromatin profiling of lung DC from naïve WT and Akita mice. Scatter plots with mean normalized reads in peaks in WT and Akita, red lines x = y. **l**, H3K27ac. **m**, H3K27me3. **n**, Flow cytometry of lung DC, representative histograms of H3K27ac. **o-s**, WT Lung DC incubated with high (50mM) or normal (10mM) glucose and 10mM ANA for 20h, then co-cultured for 4 days with OT-I CD8^+^ T cells in normal (10mM) glucose (in the absence of inhibitor), two-way ANOVA and Holm-Sidak correction. **o-p**, cDC1 incubated with high (50mM) glucose+DM (*n* = 6), normal (10mM) glucose+DM (*n* = 8), high (50mM) glucose+ANA (*n* = 8), normal (10mM) glucose+ANA (*n* = 8). **o**, Ki-67^+^CD8^+^ T cells. **p**, IFNγ^+^CD8^+^ T cells. **q-s**, cDC2 incubated with high (50mM) glucose+DM (*n* = 9), normal (10mM) glucose+DM (*n* = 8), high (50mM) glucose+ANA (*n* = 8), normal (10mM) glucose+ANA (*n* = 8). **q**, CD8^+^ T cells. **r**, Ki-67^+^CD8^+^ T cells. **s**, IFNγ^+^CD8^+^ T cells. **t-w**, WT and Akita mice infected with 50pfu PR8 and administered 5 mg/kg ANA or DMSO daily from 3 days before infection, analyzed at 10 d.p.i.: WT+DMSO (*n* = 12), WT+ANA (*n* = 12), Akita+DMSO (*n* = 13), Akita+ANA (*n* = 14), pooled data from 2 experiments. **t**, cDC1, Kruskal Wallis with Dunn’s correction. **u**, cDC2, Kruskal Wallis test with Dunn’s correction. **v**, CD64^+^ DC, one-way ANOVA with Holm-Sidak. **w**, Ki-67^+^cDC1, one-way ANOVA with Holm-Sidak. **x-z**, Mice intraperitoneally administered STZ or PBS, then infected with 50pfu PR8 and administered 5 mg/kg ANA/vehicle (DM) daily from 3 days before infection, analyzed at 10 d.p.i.: PBS+DM (*n* = 13), PBS+ANA (*n* = 10), STZ+DM (*n* = 10), STZ+ANA (*n* = 11), pooled data from 2 experiments. **x**, cDC1, Kruskal Wallis with Dunn’s correction. **y**, cDC2, one-way ANOVA with Holm-Sidak. **z**, CD64^+^ DC, one-way ANOVA with Holm-Sidak. All data mean+s.e.m. AM, Alveolar macrophage.

For functional evaluation of the potential impacts of hyperglycaemia on glycolysis, we measured the extracellular acidification rate (ECAR; Seahorse) of lung DC obtained from hyperglycaemic Akita mice and normoglycaemic WT controls. Changes in extracellular pH were used as a proxy for glycolysis-mediated lactate production. Importantly, a reduced ECAR was noted in lung DC from hyperglycaemic mice, suggesting potential impairment of lactate production compared with controls despite higher availability of a glucose substrate during hyperglycaemia (Extended Data Fig. 11o,p). No significant impact was observed on oxygen consumption rate, suggesting that mitochondrial respiration remained unaffected (Extended Data Fig. 11q). In addition, no diabetes-induced changes were noted in expression of enzymes required for lipid beta-oxidation (Extended Data Fig. 11r,s). To corroborate the unexpected reduction in ECAR, lung DC isolated from normoglycaemic and hyperglycaemic mice were incubated ex vivo with ^13^C-glucose. Tracing of isotope-labelled glucose utilization to ^13^C-labelled lactate in the supernatant showed lower levels of lactate produced by cells from hyperglycaemic animals compared with controls (Fig. 4a), which was associated with lower intracellular levels of the glycolytic metabolite pyruvate following 6 h of incubation (Extended Data Fig. 11t). Neither lung DC, nor other tested cell types from hyperglycaemic animals exhibited a deficiency in uptake of the glucose analogue 2-NBDG, suggesting that, at the in vivo setting, there is no difference in glucose uptake by immune cells under hyperglycaemic conditions (Extended Data Fig. 12a–c and Supplementary Information 15).

Importantly, elevated acetyl-CoA levels were measured in lung DC from hyperglycaemic compared with control mice (Fig. 4b), suggesting a potential rerouting of glucose metabolism away from lactate production towards acetyl-CoA during hyperglycaemia. To further investigate whether acetyl-CoA accumulation in hyperglycaemia-exposed lung DC might be linked directly to their capacity to activate T cells, we inhibited acetyl-CoA production using treatment with BMS303141, an inhibitor of ATP citrate lyase, the key enzyme converting citrate to acetyl-CoA in the tricarboxylic acid cycle^13^. Indeed, ATP citrate lyase inhibition during hyperglycaemia partially restored the capacity of lung cDC1 and cDC2 to induce T cell expansion and expression of the proliferation marker Ki-67 (Fig. 4c and Extended Data Fig. 12d–f). To further corroborate a potential detrimental role of acetyl-CoA accumulation in lung DC function we inhibited pyruvate dehydrogenase kinases (PDKs), critical negative regulators of the generation of acetyl-CoA, thereby leading to accumulation of acetyl-CoA^14^. Indeed, preincubation of lung DC with a non-toxic concentration (10 mM) of the global PDK-inhibitor dichloroacetate, followed by the addition of OT-I CD8^+^ T cells after removal of the inhibitor, reduced the capacity of cDC1 and cDC2 to induce T cell expansion (Fig. 4d and Extended Data Fig. 12g) and IFNγ production (Extended Data Fig. 12h). To corroborate these findings, we sorted lung DC from *Pdk2/3/4*-deficient mice and repeated the above cocultures with OT-I CD8^+^ T cells. Indeed, *Pdk2/3/4*-deficient DC induced altered CD8^+^ T cell expansion (Fig. 4e and Extended Data Fig. 12i), IFNγ production and expression of Ki-67 (Extended Data Fig. 12j,k) compared with WT controls. Collectively, our results suggest that hyperglycaemia probably alters the metabolic state of lung DC through excessive generation of acetyl-CoA, thereby leading to impairment of lung DC function. Inhibition of key steps in acetyl-CoA production reversed these aberrations.

## Altered chromatin landscape in lung DC

Increased acetyl-CoA levels in lung DC of diabetic animals may lead to epigenetic modifications, by potentially modulating histone acetylation which, in turn, plays a major role in chromatin remodelling and global gene expression^13^. Indeed, lung DC in diabetic and normoglycaemic animals broadly expressed the enzyme machinery that regulates epigenetic modifications (Supplementary Information 16a). To explore this possibility, we utilized the CUT&Tag epigenetic profiling of lysine 27 histone 3 modifications in DC from naïve WT and hyperglycaemic Akita mice. We selected trimethylation and acetylation at the same amino acid, reasoning that these modifications cannot co-occur, thereby enabling quantitative determination of global hyperglycaemia-induced genomic effects on lung DC. We noted a global shift from methylation to acetylation in DC obtained from hyperglycaemic animals as compared with those obtained from normoglycaemic controls (Extended Data Fig. 12l,m and Supplementary Information 16b). Immunoblotting supported elevated H3K27 acetylation (H3K27ac) in DC from hyperglycaemic mice (Fig. 4f and Supplementary Information 1). Moreover, we identified six differentially abundant peaks higher in acetylation and 60 in the methylation data, between hyperglycaemic and normoglycaemic conditions, including peaks approximating key immune genes such as *Cx3cl1*, *Fcrl5*, *Acvr1*, *Rora* and *Il17a*, genes coding for metabolic enzymes such as *Pfkfb4*, *Eci2*, *Cox6c2* and *Dgat2* and also *Hdac4* (Supplementary Information 16b). In agreement, flow cytometric analysis showed elevated H3K27ac in DC subsets from diabetic animals (Fig. 4g and Extended Data Fig. 12n). These findings indicated that hyperglycaemia probably induces global impacts on the chromatin state of lung DC.

## Epigenetic DC modulation in diabetes

Finally, we aimed to elucidate whether manipulation of the identified metabolic–epigenetic–immune DC axis could attenuate hyperglycaemia-induced lung DC dysfunction, immune impairment and exacerbated pulmonary viral disease. We began by isolation of primary lung DC from naïve WT normoglycaemic mice and incubating them with normal (10 mM) or high (50 mM) glucose, while adding anacardic acid (ANA), an inhibitor of histone-acetyl transferase that blocks excessive acetylation. Abrogation of acetylation in DC exposed to high glucose levels improved their capacity to prime CD8^+^ T cells, increased the frequency of proliferating Ki-67^+^CD8^+^ T cells and promoted their capacity to produce IFNγ (Fig. 4h and Extended Data Fig. 12o–s).

To determine the in vivo impact of hyperacetylation inhibition on altered lung DC function during respiratory infection, we treated WT and Akita mice with ANA before and during influenza virus infection. Analysis at 10 d.p.i. showed that histone-acetyl transferase inhibition in hyperglycaemic mice led to a rescue of lung cDC1, cDC2 and CD64^+^ DC (Extended Data Fig. 12t–v), coupled with a mild increase in Ki-67^+^ cDC1 (Extended Data Fig. 12w). Similarly, ANA treatment led to lung DC expansion in the STZ-induced diabetes model (Extended Data Fig. 12x–z). To assess the in vivo consequences of the reversal of lung DC hyperacetylation on CD8^+^ T cell immunity, we treated WT and diabetic Akita mice with ANA and then immunized them with OVA and polyinosinic acid:polycytidylic acid (poly I:C), mimicking a viral infection. Lung DC were then sorted 24 h post injection and transferred to naïve WT recipients, which were challenged, 10 days later, with the PR8-OVA(SIINFEKL) influenza virus. Analysis 7 days following infection showed improved viral clearance (Fig. 4i), coupled with enhanced induction of CD8^+^ T cell immunity (Fig. 4j), in mice that had received lung DC from ANA-treated hyperglycaemic mice compared with recipients of lung DC from vehicle-treated hyperglycaemic controls. Taken together, our results suggest that reversal of hyperglycaemia-induced lung DC hyperacetylation and associated epigenetic alterations may enable rescue of their ability to prime T cells, thereby constituting a potential means of ameliorating diabetes-associated immune defects.

## Discussion

Our results provide several conceptual advances. We describe the cellular and transcriptomic changes occurring during respiratory viral infection at the single-cell level (performed in 154,545 cells) and establish glucose as a critical metabolic regulator of lung DC function in steady-state and during viral infection. This result is in line with emerging evidence suggesting that metabolism impacts the function of other immune cells, such as T cells and macrophages^15,16^. We hypothesize that glucose regulation may constitute a host resistance mechanism against pathogens^17^, in which the host maintains a critical balance between the need to limit pathogen access to essential energy sources, and also fuelling protective immune responses^17^. Altered regulation of glucose control in diabetes disrupts this delicate host–microbe balance, thereby leading to enhanced susceptibility to viral infection. Similar principles may contribute to increased diabetes-related risks in viral, bacterial and fungal infections occurring in the respiratory tract and other mucosal surfaces. These merit future research. Likewise, the role of glucose in modulation of DC bone marrow development and tissue maintenance^18^ warrants future investigation.

Metabolic DC reprogramming, mainly studied in cancer and embryonic development, constitutes a critical mechanism of DC immune regulation^19^ and is mediated by a variety of signals such as TLR^20^, type I IFN^21^ and downstream acetyl-CoA-associated histone hyperacetylation^13^. Although previous studies highlighted hyperacetylation as a driver of gene activation in specific genomic loci^22^, hyperglycaemia-induced hyperacetylation may lead to the repression of immune response-related genes, possibly through chromatin destabilization and dominant aberrations in gene expression^23^. Altered H3K27 trimethylation may have lesser impacts on DC in other tissues^24^. Of note, in some contexts, H3K27 hyperacetylation may be coupled with suppressed methylation^25^, whereas in others the two processes (regulated by different mechanisms) could occur independently of each other, meriting further studies in the diabetes context. Other glucose-related processes, such as protein glycosylation or internal cell glycogen storage^26^, may also be affected by hyperglycaemia, thereby constituting interesting topics for future research.

From a translational point of view, the reconstituted antiviral immunity induced by insulin-driven correction of hyperglycaemia highlights the importance of a meticulous and proactive tight glucose control strategy in diabetics, including during acute infection. Beyond glucose control, checkpoint inhibition of aberrant DC acetylation may prevent or treat hyperglycaemia-induced immune dysfunction and its clinical ramifications. Indeed, inhibition of histone acetylation-regulating enzymes is actively explored as a treatment for cancer and other diseases involving aberrant acetylation^27^. Local administration of acetylation-targeting interventions (through inhalation) may provide an opportunity for effective rescue of lung DC immune function during pulmonary infection in diabetes, while minimizing systemic off-target effects. Putative adverse effects of such immune-reconstituting treatment, including cytokine release and chronic inflammation, merit further exploration. Likewise, validation of our findings in human diabetics inflicted with an acute pulmonary infection, such as influenza or SARS-CoV2 (ref. ^28^), merits future studies.

## Methods

### Mice

WT C57BL/6 male mice (Harlan), Akita mice^29^ (Jackson strain no. 003548) and their WT littermate controls, *Db/Db* (Jackson strain no. 000697), OT-I (C57BL/6-Tg (TcraTcrb)1100Mjb/J), OT-II (B6.Cg-Tg(TcraTcrb)425Cbn/J), Zbtb46-DTR and *Pdk2/3/4*^*−/−*^ mice^30^ were bred and maintained at the Weizmann Institute of Science animal facility under standard day/night cycles at room temperature and normal humidity and had access to ad libitum food and water. WT littermates served as controls. Animals were 8–14 weeks of age and were randomly assigned to groups. All experiments were performed in accordance with institutional guidelines and were approved by the Weizmann Institute of Science Institutional Animal Care and Usage Committee (approval nos. 05400622-2, 02800321-1, 04000520-2 and 14760619-3).

### STZ-induced diabetes

Mice received 100 mg kg^−1^ STZ (Sigma) in PBS or PBS as vehicle control via an intraperitoneal injection on days 0 and 1 and were then allowed to recover for 2 weeks before further experimental steps^31^. Hyperglycaemia in STZ-injected mice was confirmed before every experiment.

### Viral infection

We used influenza virus strain PR8 (A/Puerto Rico/34, H1N1) or OVA (SIINFEKL)-PR8 (ref. ^32^). For PVM, we used VR-1819 from the American Type Culture Collection. Mice were anaesthetized using isoflurane and then intratracheally infected with 50 µl of virus in PBS at the indicated doses. Animals were monitored daily and euthanized if they fulfilled severity criteria set out by the institutional guidelines.

### Detection of virus-specific antibodies

Blood was collected retro-orbitally using heparin-coated glass capillaries, transferred to heparin-coated tubes and kept on ice until centrifugation for 15 min at 10,000*g* and 4 °C. Serum was transferred to fresh tubes and stored at −80 °C until use. BAL was collected by insertion of a catheter into the trachea and flushing the lungs with 1 ml of PBS. Samples were stored on ice until centrifugation for 5 min at 500*g* to remove the cellular fraction. BAL fluid was stored at −80 °C until further use. For detection of antiviral antibodies, serum and BAL fluid were measured for virus-specific IgM and IgG2b antibody levels; 96-well plates (Maxisorp, Nunc) were coated with ultraviolet-inactivated influenza virus (PR8) in PBS overnight at 4 °C. Plates were washed and incubated with PBS-1% bovine serum albumin (BSA) for 2 h at room temperature for blocking. BAL fluids from individual mice were serially diluted in PBS with 0.1% BSA, starting with a 1:3 dilution for BAL fluids and 1:50 dilution for sera, followed by incubation at room temperature for 2 h. Plates were washed five times and incubated with alkaline phosphate-labelled goat anti-mouse antibodies to IgM or IgG2b (Southern Biotech Technologies, Inc.) at 1:1,000 dilution in PBS with 0.1% BSA at room temperature for 2 h. Thereafter, plates were washed five times and substrate p-nitrophenyl phosphate (Sigma-Aldrich) was added. Optical densities were measured on an enzyme-linked immunosorbent assay reader (Bucher Biotec) at 405 nm. For analysis, antibody titre data were analysed by fitting function *y* = *a* × *x*/(*b* + *x*) + *c* to each sample using nonlinear least-squares regression model, where *x* is 1/antibody dilution, *a* is the saturation signal in ELISA, *b* is the concentration of the antibody required to achieve 50% of saturation signal in ELISA and *c* is the background signal. Half-maximal effective concentrations (inflection points), which are indicative of the concentration of the antibody required to achieve 50% of saturation signal in ELISA, were then used to perform statistical testing.

### Flow cytometry

Mice were euthanized by intraperitoneal injection of 200 mg ml^−1^ sodium pentobarbital. Lungs were perfused with cold PBS and put on ice after removal. LdNs were collected and then digested with 2 mg ml^−1^ type IV collagenase (Worthington) and 1 mg ml^−1^ DNase I (Sigma) at 37 °C for 20 min in Iscove’s modified Dulbecco’s medium (IMDM), and subsequently passed through a 70 µm cell strainer using 10 ml of PBS. Lungs were minced and digested with 1 mg ml^−1^ Hyaluronidase (Sigma), 25 μg ml^−1^ Collagenase XI (Sigma), 50 μg ml^−1^ Liberase TM (Roche) and 1 mg ml^−1^ DNase I (Sigma) in IMDM at 37 °C for 30 min, and subsequently passed through a 70 µm cell strainer using 20 ml of PBS. Cells were then centrifuged for 10 min at 500*g* and resuspended with 10 ml of BS. Cells were centrifuged for 7 min at 500*g* before resuspension in 1 ml of PBS. Samples were stained using Zombie viability dye (BioLegend) according to the manufacturer’s instructions. Fc receptors were blocked using 1 μg ml^−1^ anti*-*CD16/32 (BioLegend) 1:200. Cells were stained with the following anti-mouse antibodies at the indicated dilutions: CD11c APC-Cy7 (N418) 1:200, CD11b BV605 (M1/70) 1:1,000, Siglec*-*F PE (E50^*-*^2440, BD Biosciences) 1:1,000, CD45 AF700 (30*-*F11) 1:1,000, CD45 BV711 (30-F11) 1:1,000, CD45.2 (104) BV711 1:200, CD4 PerCP-Cy5.5 (GK1.5) 1:200, CD4 (GK1.5) FITC 1:200, CD8 (53*-*6.7) BV605 1:200, CD8 (53-6.7) PE 1:1,000, IL-4 PE (11B11) 1:200, IL-5 BV421 (TRFK5) 1:200, IFNγ PE-CF594 (XMG1.2) 1:500, Ly-6G PerCP-Cy5.5 (1A8-Ly-6g) 1:1,000, MHC class II BV421 (M5/114.15.2) 1:1,000, CD64 APC (X54^*-*^5/7.1) 1:1,000, CD19 BV605 (6D5) 1:200, CD19 (6D5) APC (1:200), CD90.2 BV605 (30-H12) 1:1,000, TCR-β APC-Cy7 (H57-597) 1:200, TCRγδ PerCP-Cy5.5 (GL3) 1:200, NK1.1 APC (PK136, eBioscience) 1:200, FcεRIα PE_CF594 (MAR-1, Thermo Fisher Scientific) 1:1,000, XCR1 BV510 (ZET) 1:500, GATA3 BV421 (16E10A23) 1:100, RORγt PE (AFKJS-9, Thermo Fisher Scientific) 1:1,000, IL-17A PerCP-Cy5.5 (TC1118H10.1) 1:200, IL-13 AF488 (eBioscience, eBio13A) 1:200, T-bet PE_Cy7 (4B10) 1:800, FoxP3 PE (MF-14) 1:200, CD40 (3/23) PerCP 1:100, CD80 PE-Cy7 (16-10A1) 1:200, CD86 PE-CF594 1:1,000 (GL-1), F4/80 (BM8) PE-Cy7 1:200, Ly-6C PE-Cy7 (HK1.4) 1:1,000, all purchased from BioLegend unless otherwise stated. BV421-conjugated peptide-MHC class I tetramers (H-2Db/NP34), with the NP34 peptide (NP366-374, ASNENMETM) from the nucleoprotein of influenza virus A/PR/8/34, were obtained from the NIH tetramer core facility and used at 1:400. For sorting experiments, dead cells were excluded using the live/dead marker DAPI (BioLegend, 20 ng ml^−1^). For intracellular staining of transcription factors or histone modifications, cells were fixed and stained using the intracellular fixation and permeabilization kit (eBioscience) according to the manufacturer’s instructions. For staining of H3K27ac, cells were stained with primary rabbit anti-H3K27ac antibody (abcam, 1:400) followed by secondary goat anti-rabbit AF647 (Invitrogen, 1:1,000). For cytokine analysis, T cells were restimulated with a cell activation cocktail (BioLegend) containing Brefeldin A for 4 h. For cytokine analysis of myeloid cells, cells were incubated for 4 h with Brefeldin A only. Flow cytometry analysis was performed on a LSR Fortessa (BD) or ARIA III (BD) using DIVA software (BD) and analysed with FlowJo (Tree Star). Cells were identified in the following way: CD4^+^ T cells as CD45^+^TCRb^+^CD4^+^, CD8^+^ T cells as CD45^+^TCRb^+^CD8^+^, B cells as CD45^+^CD19^+^TCRb^−^, eosinophils as CD45^+^CD11c^−^CD11b^+^Siglec-F^+^SSC-A^high^, neutrophils as CD45^+^CD11c^−^CD11b^+^Ly-6G^+^, lung cDC1 as Siglec-F^−^MHCII^+^CD11c^+^XCR1^+^, lung cDC2 as Siglec-F^−^MHCII^+^CD11c^+^XCR1^−^CD11b^+^CD64^−^, lung CD64^+^ DC as Siglec-F^−^MHCII^+^CD11c^+^XCR1^−^CD11b^+^CD64^+^, dLN cDC2 as CD45^+^autofluorescent^−^CD11c^+^MHCII^high^XCR1^+^, lung dLN cDC1 as CD45^+^autofluorescent^−^CD11c^+^MHCII^high^XCR1^−^CD11b^+^CD64^−^, Ly-6C^high^ monocytes as Siglec-F^−^Ly-6G^−^CD11b^+^Ly-6C^high^ and Ly-6C^low^ monocytes as Siglec-F^−^Ly-6G^−^CD11b^+^Ly-6C^low^.

### Ex vivo DC experiments

Lung DC were isolated from WT mice using the tissue preparation protocol described above. Following the establishment of a single-cell suspension, cells were incubated for 1 h on ice with anti-MHCII beads (Miltenyi) and the enriched fraction was collected using LS MACS columns (Miltenyi). Cells were then stained and cDC1 and cDC2 were sorted. DC were then incubated in RPMI 1640 (without glucose) with 20% fetal calf serum, glutamine, HEPES, penicillin/streptomycin and low (10 mM) or high (50 mM) glucose for 20 h before either analysis or replacement with fresh low-glucose medium and the addition of T cells (see below). Where indicated, either 2-DG (5 mM; Sigma), BMS303141 (10 mM; Sigma) or ANA (20 mM; Sigma) was added.

### Seahorse

Cells were seeded at 1 × 10^5^ cells per well in an Xfe96 analyser (Agilent) and ECAR or oxygen consumption rate was measured over 80 min with the addition of 10 mM glucose, 1 µM oligomycin and 50 mM 2-DG (for ECAR) and 1 µM oligomycin, 1 µM carbonyl cyanide 4-(trifluoromethoxy) phenylhydrazone and 0.5 µM actinomycin and rotenone at the indicated time points.

### Ex vivo metabolite analysis

Cells (1–2 × 10^5^) were incubated in RPMI medium (Biological Industries, no. 01-101-1 A) with 10% dialysed FBS (Gibco), 4 mM glutamine and 11 mM U-13C6d-glucose (Cambridge Isotope Laboratories, no. 1396) for 6 h. At every time point, 22 µl of medium was briefly centrifuged to remove cell debris and snap-frozen in liquid nitrogen. Medium (20 µl) from each time point was extracted with 400 µl of ice-cold 80:20 methanol:water with 2 µg ml^−1^ ribitol as an internal standard (extraction solvent), vortexed and immediately centrifuged at 18,000*g* for 15 min at 4 °C. Supernatants were collected and dried by vacuum (Speed Vac and lyophilizer). Dried samples were incubated with 20 µl of methoxyamine hydrochloride (Alfa Aesar, no. A19188; 20 mg ml^−1^ in pyridine) at 37 °C for 90 min with shaking, followed by incubation with 35 µl of *N*, *O*-bis trimethylsilyl trifluoroacetamide (Sigma, no. 15222) at 37 °C for 30 min. Gas chromatography–mass spectrometry was performed using gas chromatograph no. 7820AN (Agilent Technologies) interfaced with mass spectrometer no. 5975 (Agilent Technologies), with a HP-5ms capillary column 30 m 250 mm 0.25 mm (no. 19091S-433, Agilent Technologies). Helium carrier gas was flowed at a constant rate of 1.0 ml min^−1^. Gas chromatograph column temperature was programmed from 70 to 150 °C via a ramp of 4 °C min^−1^, from 250 to 215 °C via a ramp of 9 °C min^−1^, from 215 to 300 °C via a ramp of 25 °C min^−1^ and maintained at 300 °C for 5 min. Mass spectrometry was performed by electron impact ionization and operated in full-scan mode from 30 to 500 *m*/*z*. Inlet and mass spectrometer transfer line temperatures were 280 °C and ion source temperature was 250 °C. Sample injection (1 µl) was in splitless mode. Raw data signals obtained from gas chromatography–mass spectrometry were analysed using MassHunter software (Agilent Technologies). Isotopologue distribution of lactate was corrected for naturally occurring isotopes using IsoCor software^33^.

### scRNA-seq

Animals were euthanized by intravenous pentobarbital and lungs perfused with cold PBS. Subsequently, 1 ml of 5 mg ml^−1^ dispase solution (Sigma) was injected through a tracheal catheter. The solution was incubated for 5 min before the lungs were excised and placed in IMDM (Gibco) on ice. Lungs were then minced and incubated with an enzyme mix containing 1 mg ml^−1^ DNase I (Sigma), 2 mg ml^−1^ Collagenase IV (Worthington) and 5 mg ml^−1^ dispase (Sigma) for 20 min in a shaker at 37 °C. The digested extract was then transferred to a 70 µm cell strainer and smashed through with 20 ml of PBS to obtain a single-cell suspension. Cells were centrifuged at 4 °C for 10 min at 500*g*. The pellets were resuspended in 1 ml of ACK buffer (Gibco, no. A1049201) and incubated for 1 min at room temperature, followed by the addition of 14 ml of PBS, and cells were centrifuged at 500*g* for 7 min. Cells were incubated for 1 h on ice in 500 µl of dead cell depletion kit beads (Miltenyi), after which 5.5 ml of kit buffer was added to each sample. Samples were filtered and loaded onto LS columns (Miltenyi) and the flowthrough collected. For experiments using cellular fractions enriched for DC, 50 µl each of CD19 and CD90 microbeads was added during the bead incubation step. Flowthrough was centrifuged for 7 min at 500*g* and resuspended in PBS with 0.04% BSA for samples that were to be sent directly for scRNA-seq. For each sample the single-cell suspension was loaded onto Chromium, aiming for 6,000 cells. Libraries were prepared according to the 10X Genomics Chromium Single Cell 3′ Reagent Kits User Guide (v.3 Chemistry). In each experiment, both WT and Akita mice were sequenced to avoid confounding from batch effect. Libraries were sequenced using either an Illumina Nextseq 550 or a Novaseq 6000. On Nextseq, libraries were sequenced according to the guidelines except that 56 base pairs were sequenced on R2 and 89 on Novaseq R2.

For scRNA-seq experiments with cell hashing, cells were incubated for 5 min with 1 µg ml^−1^ anti*-*CD16/32 (BioLegend) to block Fc receptors before the addition of the antibody mix including hashing antibodies. Cells were incubated for 30 min on ice, 10 ml of PBS was added and cells were then centrifuged for 7 min at 500*g*. The supernatant was removed and cells were resuspended in PBS, filtered through a 40 µm mesh and sorted by flow cytometry as described above. Sorted cells were centrifuged for 7 min at 500*g*, resuspended in PBS with 0.04% BSA and counted before being sent for scRNA-seq. For each sample, the single-cell suspension was loaded onto Chromium and libraries prepared as described above. Using hashing antibodies (BioLegend TotalSeq B0303, B0304, B0305 and B0306), we pooled two control and two STZ-treated mice per sample. Sequencing libraries for transcriptomes and hashtags were prepared according to the 10X Genomics Chromium Single Cell 3′ Reagent Kits User Guide with Feature Barcoding Technology (v.3.1 Chemistry). Subsequently, libraries were sequenced on a Novaseq 6000, with 89 base pair R2.

For analysis, bcl files were demultiplexed and converted to fastq files using bcl2fastq v.2.20.0.422 (mkfastq function in the CellRanger pipeline v.6.0.0). In experiments with Akita mice, reads from Novaseq were trimmed to 56 base pairs with trimmomatic v.0.36 that was sequenced on Nextseq to ensure comparability between samples. Subsequently, reads were mapped to the mouse genome (mm10) combined with the influenza A genome (Puerto Rico 1934, H1N1) and gene expression was quantified using the unique molecular identifier count function in CellRanger pipeline v.6.0.0). For Akita experiments, 26 samples (four day 0 Akita, four day 0 WT, five day 1 Akita, five day 1 WT, four day 10 Akita and four day 10 WT) were then aggregated with the aggr function in the CellRanger pipeline. Cell number for specific populations was obtained by multiplying cell type frequency from single-cell data by total lung cell count. For STZ, two single-cell libraries with four hashed samples in each were also aggregated.

### Analysis of whole-lung single-cell transcriptomes

First, cells with fewer than 600 detected transcripts, fewer than 200 detected genes and over 20% mitochondrial reads were removed. Doublets were identified by finding clusters of cells displaying gene expression patterns of two cell types simultaneously, and were removed. Data were then normalized using the LogNormalize method from NormalizeData function, and 2,000 highly variable genes were identified using the ‘vst’ method. We calculated principal component analysis using highly variable genes, and *k*-nearest neighbour for the dataset was computed using the first 30 principal components. Finally, cells were clustered using a shared nearest-neighbour modularity optimization-based clustering algorithm. All functions mentioned above are part of the Seurat v.4.0.1 package in R^34,35^. To achieve better clustering results, we used a stepwise approach and divided the obtained clusters into five datasets based on their markers, containing immune cells, epithelial cells, endothelial cells, stromal cells and cells expressing high levels of cell cycle genes. For each of the subsets, variable genes, principal component analysis, clustering and UMAP were recalculated. Following reclustering—because immune cells featured a significant heterogeneity—we decided to further subdivide into T cells, B cells, mononuclear phagocytes and the remainder immune cell fraction (neutrophils and basophils). Because we were particularly interested in DC, mononuclear phagocytes were subdivided again into monocytes/macrophages and DC. All of these clusters were annotated using the Immgen database^36^ and previously published data^36–39^. For analysis, cells with fewer than 300 detected genes and over 20% mitochondrial reads were removed. Contaminant cells (mostly NK and B cells) were identified by finding clusters of cells expressing gene expression patterns of cell types other than DC and were then removed. Quantified hashtags were used to demultiplex samples—we considered a hashtag as a single if particular barcode abundance was higher than five times that of the second-most abundant. Cells for which hashtags were not identified, or if more than one hashtag was found, were removed. Data were then normalized, scaled and clustered as described above for Akita mice. We used our single-cell transcriptomic data from the whole lung as a reference for cell type annotation. Differential expression analysis between conditions was performed on pseudobulk counts for each cell type in each sample using DESeq2 (ref. ^40^). Functional analysis of differentially expressed genes, ordered according to adjusted *P* values, was performed using g:Profiler2 with default settings and multiple hypothesis testing adjustment using all mouse genes as background control^41^. The log_10_ false discovery rate-adjusted *P* values were plotted as barplots. For heatmaps, Gene Ontology lists were obtained from Ensembl BioMart. KEGG pathway enrichment analysis was performed using all differentially expressed genes higher in PBS controls than in STZ-treated animals, with the gProfiler2 R package using standard settings.

### CUT&Tag chromatin profiling

H3K27 trimethylation (H3K27me3) and H3K27ac were profiled from sorted DC using a modified CUT&RUN protocol^30^. Nuclei were extracted from 200,000 sorted cells. Cells were collected by centrifugation at 500*g* for 5 min at 4 °C and gently resuspended in 50 µl of lysis buffer containing 10 mM Tris pH 7.4, 10 mM NaCl, 3 mM MgCl_2_, 1% BSA, 0.1% Tween-20, 0.1% IGEPAL, 0.01% Digitonin (Promega), 1 mM DTT and 1× complete protease inhibitor with incubation on ice for 3 min. Wash buffer (50  µl) containing 10 mM Tris pH 7.4, 10 mM NaCl, 3 mM MgCl_2_, 1% BSA, 0.1% Tween-20, 1 mM DTT and 1× complete protease inhibitor was added. Nuclei were collected by centrifugation of 500*g* for 5 min at 4 °C and resuspended in 100 µl of room temperature NE Buffer. Concanavalin A beads (5  µl per sample; Epicypher) were activated by washing beads twice with 100 µl 10 µl^−1^ cold Bead Activation Buffer (20 mM HEPES pH 7.9, 10 mM KCl, 1 mM CaCl_2_, 1 mM MnCl_2_). Beads were resuspended in 10 µl per sample cold Bead Activation Buffer. Nuclei were incubated with concanavalin beads at room temperature for 10 min. Beads were placed on the magnet, supernatant was removed and nuclei were resuspended in 50 µl of cold wash buffer (20 mM HEPES pH 7.5, 150 mM NaCl, 0.5 mM spermidine, 1× complete protease inhibitor, 2 mM EDTA). Primary antibody (0.5  µl of H3K27me3 antibody, no. C36B11, Cell Signaling; H3K27ac antibody, no. D5E4, Cell Signaling; Isotype control antibody, no. DA1E, Cell Signaling) was added to each sample and incubated overnight at the nutator at 4 °C. Beads were placed on the magnet, supernatant was removed and nuclei were resuspended in 50 µl of digitonin buffer (20 mM HEPES pH 7.5, 150 mM NaCl, 0.5 mM spermidine, 1×  complete protease inhibitor, 0.01% digitonin, 2 mM EDTA) and 0.5 µg of anti-rabbit secondary antibody, Epicypher was added, mixed and incubated for 30 min at room temperature. While beads were on the magnet, they were washed twice with 200 µl of cold digitonin buffer and then resuspended in 50 µl of high-salt digitonin buffer (20 mM HEPES pH 7.5, 300 mM NaCl, 0.5 mM spermidine, 1× complete protease inhibitor, 0.01% digitonin, 2 mM EDTA); 2.5 µl of pAG-Tn5 (Epicypher) was then added to each sample, followed by vortexing and incubation for 1 h at room temperature. Nuclei were washed twice with high-salt digitonin buffer and 50 µl of cold tagmentation buffer was added (20 mM HEPES pH 7.5, 300 mM NaCl, 10 mM MgCl_2_, 0.5 mM spermidine, 1× complete protease inhibitor) followed by incubation for 1 h at 37 °C. Following tagmentation, nuclei were washed with 50 µl of 10 mM *N*-[tris(hydroxymethyl)methyl]-3-aminopropanesulfonic acid (TAPS) pH 8.5 and 0.2 mM EDTA. To release DNA fragments, 5 µl of 10 mM TAPS pH 8.5 with 0.1% SDS was added to nuclei with incubation for 1 h at 58 °C. Subsequently, 15 µl of 0.67% Triton-X was added to neutralize SDS. Into 20 µl of sample, 25 µl of non-hot-start CUTANA High Fidelity 2× PCR Master Mix and 2 µl each of primers i5 and i7 (Illumina) were added with incubation at 58 °C for 5 min, 72 °C for 5 min, 98 °C for 45 min and then 18 cycles of 15 s at 98 °C and 10 s at 60 °C, followed by a final extension at 72 °C for 1 min. Libraries were cleaned up using 1.3× AMPure beads as per the manufacturer’s recommendations and eluted in 15 µl of TE buffer.

For analysis, bcl files were demultiplexed and converted to fastq files with bcl2fastq v.2.20.0.422. Subsequently, reads were trimmed to remove adaptors using fastp v.0.23.0 with standard parameters. Mapping to the GRCm38 genome was performed using bowtie2 v.2.3.5.1 and the following parameters: --local --very-sensitive-local --no-unal --no-mixed --no-discordant --phred33 -I 10 -X 700, and deduplicated with picard v.2.22.8 (ref. ^42^). Files were converted with SAMtools v.1.9 and BEDtools v.2.26.0 to generate bedgraph files^43^. Peaks were called with SEACR v.1.3 using isotype control data as background, thereby identifying genes closest to peaks^44^. Data were subsampled to have the same coverage across samples, and reads in peaks were counted with BEDtools multicov^43^. To find differentially abundant peaks we used DESeq2 (ref. ^40^).

### DC T cell coculture

cDC1 and cDC2 were sorted from naïve lungs of WT or Akita mice. CD8^+^ T cells were obtained from splenocytes of T cell receptor-transgenic OT-I mice, in which all CD8^+^ T cells recognize the SIINFEKL peptide of OVA. Isolation was performed using CD90.2 MACS-bead (Miltenyi) pre-enrichment and subsequent flow cytometry-based sorting of CD8^+^CD11c^−^ aufluorescent-negative cells. T cells and DC were then cocultured for 4 days in complete IMDM medium (Life Technologies) with 100 mg ml^−1^ OVA protein_._

### Model of allergic airway inflammation

Mice received 10 μg of HDM in 50 µl of PBS (Citeq) on day 0 for sensitization, and were then challenged daily with 10 μg of HDM on days 7–11 with analysis on day 14.

### Transfer of HDM-pulsed lung DC

Mice were intratracheally administered 200 μg of HDM 24 h post infection. Lung DC were sorted and intratracheally transferred to naïve WT recipients. Each mouse received 5 × 10^5^ cells. Animals were then challenged daily with 10 μg of HDM on days 7–11, analysis performed on day 14.

### Transfer of UV-PR8-pulsed lung DC

PR8 influenza virus was inactivated under an ultraviolet lamp for 30 min. Mice were intratracheally administered 2.5 × 10^6^ plaque-forming units (pfu) inactivated PR8 20 h post infection. Lung DC sorted and transferred to naïve recipients with 3 × 10^5^ cells per recipient. After 10 days, animals were infected with 500 pfu PR8 and euthanized 7 days post infection.

### DC-mediated antigen uptake and transport

Animals intratracheally received 200 μg of Ovalbumin-AlexaFluor647 (Invitrogen) and either 100 μg of HDM (Citeq) or 500 pfu PR8. Lungs and dLN were collected at the indicated time points.

### Bone marrow chimeras

WT recipients were sublethally irradiated with 9.5 Gy and transplanted with Zbtb46-DTR bone marrow 1 day later. Animals were used for experiments 8 weeks following bone marrow reconstitution. In relevant groups, animals received 20 mg per kg^−1^ bodyweight diphtheria toxin every other day for 10 days.

### Free fluid measurement

Free fluid was measured using nuclear magnetic resonance with a Bruker minispec LF50/mq7.5 MHz live mouse analyser.

### Histology

Lungs were removed and fixed in 4% formaldehyde. Tissues were processed and stained with haematoxylin and eosin. Lungs were evaluated in a blind manner by a certified pathologist scoring according to the severity of inflammation (inflammatory score).

### Immunoblotting

Cells were lysed in RIPA buffer containing protease and phosphatase inhibitors, incubated for 30 min at 4 °C and centrifuged at 15,000*g* for 10 min at 4 °C. Samples were run on 12% acrylamide gels and transferred to nitrocellulose membranes. Immunoblot analysis was performed using anti‐H3K27ac polyclonal antibody (no. ab4729, 1:1,000; Abcam), beta-actin (no. MA5-15739, 1:1,000, Thermo), goat anti-mouse HRP (no. 115-035-205, 1:5,000, Jackson Laboratories) and goat anti-rabbit HRP (no. 111-035-003, 1:5,000, Jackson Laboratories). Immunoblot imaging and band intensity quantification were performed using the Gel Doc XR+ system (Bio-Rad).

### RNA isolation and quantitative PCR

Lung lobes were collected in Trizol (Bio-Lab), frozen on dry ice and stored at −80 °C until required for RNA isolation. To isolate RNA, samples were thawed, a metal bead was added and cells were lysed in a tissue lyser (Qiagen) at 30 Hz for 2 min. Next, 200 µl of chloroform was added and samples were vortexed and centrifuged at 13,000 rpm for 15 min at 4 °C. The clear layer was transferred to a fresh tube, 500 µl of isopropanol was added and the sample was precipitated at −20 °C overnight. Samples were centrifuged for 8 min at 10,000 rpm at 4 °C and pellets were washed once in ethanol and resuspended in 50 µl of DNase digestion mix (Sigma). Samples were treated according to the manufacturer’s instructions. Subsequently 1 µg of RNA was reverse transcribed using a high-capacity reverse transcription kit (Applied Biosystems). For quantitative PCR of host and viral genes, DNA templates were diluted to obtain 1 µg per reaction. Amplifications were performed with the following primer sets: PR8 forward, 5′-AGATGAGTCTTCTAACCGAGGTCG-3′; PR8 reverse, 5′-TGCAAAAACATCTTCAAGTCTCTG-3′; PVM forward, 5′-AGGACTCTGCCAGATGGTTG-3′; PVM reverse, 5′ CAGGGAAACTCAAAGGGTCA-3′; HPRT forward, 5′-TCAGTCAACGGGGGACATAAA-3′; HPRT reverse, 5′-GGGGCTGTACTGCTTAACCAG-3′; Ifnb1 forward, 5′-TCCGAGCAGAGATCTTCAGGAA-3′; Ifnb1 reverse, 5′-TGCAACCACCACTCATTCTGAG-3′. Fast SYBR Green Master Mix (Thermo Fisher Scientific) was used in duplicates. Amplification conditions were as follows: denaturation at 95 °C for 20 s, followed by 40 cycles of denaturation 95 °C for 1 s; annealing at 60 °C for 20 s followed by the melting curve. Data were analysed using the ∆Ct method.

### Targeted metabolomics

#### Acetyl-CoA

Cells were sorted into 100% methanol to give a final concentration of 60% methanol and frozen in liquid nitrogen. The buffer was evaporated using speedvac and stored at −80 °C until further processing. For liquid chromatography–tandem mass spectrometry (LC–MS/MS), samples were thawed on ice and ultrasonicated at 4 °C and 13,000*g* for 10 min. Supernatants were transferred to new tubes, lyophilized and reconstituted in LC–MS-grade water. Chromatography was performed using a Shimadzu UHPLC System on an Xselect column (HSS T3, 3.5 µm particle size, 100 Å pore size, 100 × 2.1 mm^2^). Injection volume was 20 μl, oven temperature was maintained at 40 °C and autosampler temperature was maintained at 5 °C. Chromatographic separation was achieved using a linear gradient programme at a constant flow rate (350 µl min^−1^) over a total run time of 7 min, from 83 to 5% solvent A (10 mM ammonium acetate in water pH 9.0). Methanol:water (1:1) was used for washing the needle before each injection cycle. All samples were analysed in duplicate. Acetyl-CoA was detected using an AB Sciex Triple Quad 5500 mass spectrometer in negative-ion mode, with electrospray ionization and multiple-reaction monitoring mode of acquisition. The IonDriveTM Turbo V source temperature was set at 450 °C, with ion spray voltage at 5,000 V. The curtain gas was set at 30.0 psi. The nebulizer gas (gas 1) was set to 50 psi, the turbo heater gas (gas 2) was set to 50 psi, the collision gas was set to high and dwell time was 20 ms. Two transitions were monitored: *m*/*z* 303.1 (quantifier) and *m*/*z* 428 (qualifier). Data acquisition was performed using Analyst 1.7.1, and data were analysed with Sciex OS Software.

#### Pyruvate

Cells were lysed and extracted twice with 100 µl of 80% aqueous methanol. Insoluble material was pelleted in a centrifuge at 4 °C. The supernatant was collected and evaporated. The residue was resuspended in 100 µl of water and used for consequent LC–MS/MS analyses through derivatization, as previously described^45^. In brief, 100 µl of standard or sample was mixed with 50 μl of 35 mM 3-nitrophenylhydrazine (Sigma) in 75% methanol, 50 μl of 105 mM *N*-(3-dimethylaminopropyl)-*N*′-ethylcarbodiimide (Sigma) in methanol and 50 μl of 2.5% pyridine in methanol. The reaction mixture was shaken at 4 °C for 30 min and then evaporated. The residue was resuspended in 50 μl of 100% methanol, filtered and used for LC–MS/MS analysis. The LC–MS/MS instrument comprised an Acquity I-class UPLC system (Waters) and Xevo TQ-S triple quadrupole mass spectrometer (Waters), equipped with an electrospray ion source and operated in negative-ion mode. MassLynx and TargetLynx software (v.4.2, Waters) were applied for the acquisition and analysis of data. Chromatographic separation was performed on a 150 × 2.1-mm^2^-internal diameter, 1.8 μm UPLC HSS T3 column (Waters Acquity) with mobile phases A (0.03% aqueous formic acid) and B (0.03% formic acid in acetonitrile) at a flow rate of 0.3 ml min^−1^ and column temperature of 40 °C. The gradient used was as follows: for the first 1.5 min, the column was held at 5% B, then to 35 min a linear increase to 73% B and for the following 30 s to 90% B, then to 35.5–38.0 min back to 5% B and equilibration at 5% B for 7 min. Samples maintained at 4 °C were automatically injected in a volume of 5 μl. For MS, argon was used as the collision gas at a flow of 0.10 ml min^−1^. Capillary voltage was set to 2.23 kV, source temperature to 150 °C, desolvation temperature to 400 °C, cone gas flow to 150 l h^−1^ and desolvation gas flow to 800 l h^−1^. Multiple-reaction monitoring transitions for pyruvate were as follows: 357.2 (^12^C), 358.2 (^13^C_1_), 359.2 (^13^C_2_) and 360.2 (^13^C_3_) *m*/*z* for parent ions and 136.9 *m*/*z* for the fragment ion, with collision energy 15 eV.

### Insulin supplementation

Osmotic minipumps (Alzet, model 2004) were used (infusing the compound at a rate of 0.25 µl h^−1^ for 28 days). The pumps were filled with 200 µl of human insulin (Sigma) diluted in PBS (-Ca^2+^, -Mg^2+^). Vehicle control pumps contained an equivalent volume of PBS (-Ca^2+^, -Mg^2+^). Mice were anaesthetized by intraperitoneal injection of ketamine (100 mg kg^−1^) and xylazine (10 mg kg^−1^). The skin of the neck was shaved and disinfected with 70% ethanol. An incision was made in the skin and osmotic pumps subcutaneously inserted following minimal dissection and placed above the right hind flank. The cut was closed with sterile surgical clips and mice were frequently monitored for any signs of stress, bleeding, pain, discharge or abnormal behaviour.

### ANA treatment

Mice received 5 mg kg^−1^ ANA (Sigma) in 100 µl of corn oil (Sigma) intraperitoneally daily for the indicated durations.

### 2-NBDG treatment

Mice received 100 μl of 5 mM 2-NBDG (Thermo Fisher Scientific) intravenously and euthanized after 30 min.

### 2-DG treatment

Mice received 240 mg kg^−1^ 2-DG (Sigma) in 200 µl of PBS intraperitoneally for the indicated durations.

### Plasma parameters

Blood samples were centrifuged for 15 min at 4 °C and 12,000*g* to separate plasma from cells. Enzyme activity and molecule concentrations were measured using a Roche Cobas 111 serum analyser according to the manufacturer’s instructions.

### Insulin measurement

Plasma insulin was measured using the Ultrasensitive Mouse Insulin ELISA kit (Crystal Chem) according to the manufacturer’s instructions.

### Statistical analysis and reproducibility

Statistical tests were performed using GraphPad Prism 9.2 and R. For pooled analysis of results from different independent repeats, all mice from the same experimental group were pooled and a new statistical comparison was made for the entire pooled experiment, as performed for individual repeats. All measurements were taken from distinct samples. For all datasets, normality was calculated using the Shapiro–Wilk test and parametric or non-parametric tests were used accordingly. For comparisons between two groups with normal distribution, a two-sided unpaired *t*-test was performed; for comparisons between two groups with non-normal distribution, a two-sided Mann–Whitney *U*-test was used; for comparison of more than two groups, ANOVA was used with correction for multiple comparisons using Holm–Sidak for parametric-distributed datasets and the Kruskal–Wallis test with Dunn’s correction for non-parametric-distributed datasets. All exact *P* values are presented in Supplementary Table 1. *P* < 0.05 was considered significant; *P < 0.05, **P < 0.01, ***P < 0.001, ****P < 0.0001. All experiments were repeated between two and seven times.

### Reporting summary

Further information on research design is available in the Nature Portfolio Reporting Summary linked to this article.

## Online content

Any methods, additional references, Nature Portfolio reporting summaries, source data, extended data, supplementary information, acknowledgements, peer review information; details of author contributions and competing interests; and statements of data and code availability are available at 10.1038/s41586-023-06803-0.

### Supplementary information

Supplementary Information Supplementary Figs. 1–16.

Reporting Summary

Supplementary Table 1 *P* values and statistics.

## Data Availability

All raw sequencing data are deposited to Array Express with the following accession numbers: scRNA-seq raw data from WT and Akita mice over the course of infection, E-MTAB-11394; hashed scRNA-seq data of DC from STZ model, E-MTAB-11393; CUT&RUN data, E-MTAB-11390.
