# Supplementary material for: Lung dendritic-cell metabolism underlies susceptibility to viral infection in diabetes
Source: Nature. 2023 Dec 13;624(7992):645–52. doi: 10.1038/s41586-023-06803-0 (PMC10733144; doi:10.1038/s41586-023-06803-0)
Supplement: Supplementary file 1 — Supplementary Figs. 1–16. [file 41586_2023_6803_MOESM1_ESM.pdf]

nature portfolio

https://doi.org/10.1038/s41586-023-06803-0

Supplementary information

Lung dendritic-cell metabolism underlies susceptibility to viral infection in diabetes

In the format provided by the authors and unedited

Supplementary information 1

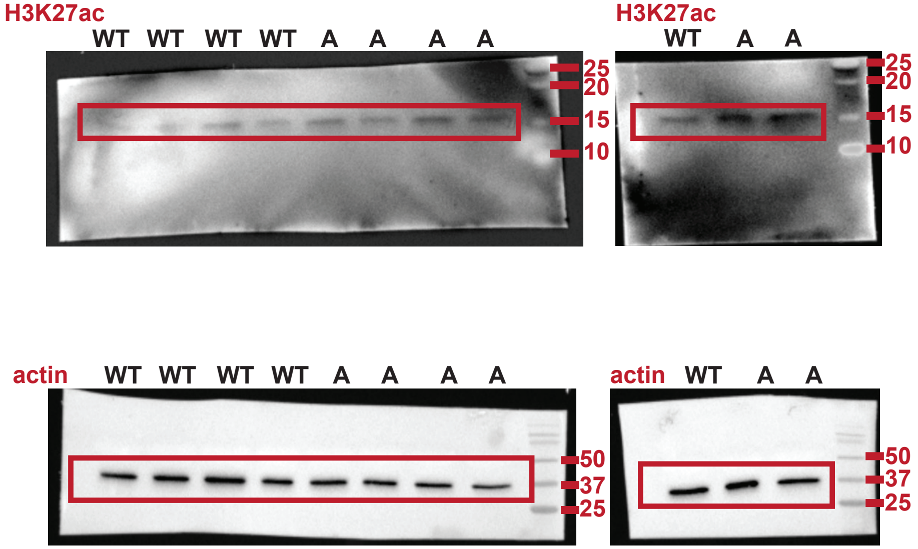

Supplementary information 1: Hyperglycaemia dysregulates H3K27Ac in lung DC.
Raw Western blot gels of H3K27ac in lung DC from WT and Akita mice. A-Akita.

Supplementary information 2

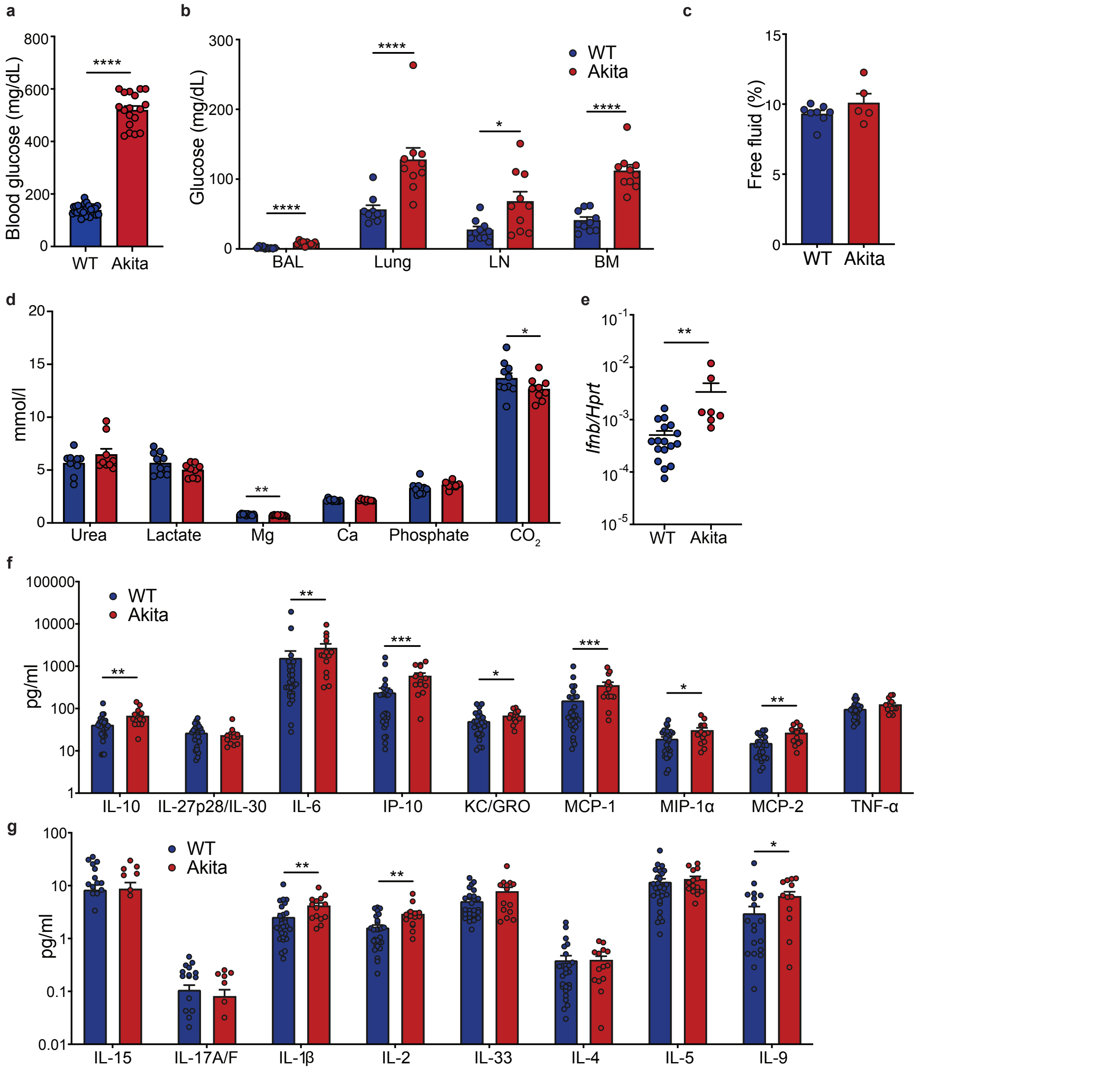

Supplementary information 2: Diabetes is associated with high glucose levels in multiple tissues.
a, Blood glucose in naïve WT (*n* = 27) and Akita (*n* = 18) mice, two-sided unpaired t-test.
b, Glucose in indicated organs in naïve WT (*n* = 10) and Akita (*n* = 10) mice, two-sided unpaired t-test for LN and BM, two-sided Mann Whitney U-test for BAL and lung.
c, Free fluid using NMR body composition WT (*n* = 8) and Akita (*n* = 5) mice, two-sided unpaired t-test.
d, Serum parameters, naïve WT (*n* = 10) and Akita (*n* = 9) mice, unpaired t-test for lactate, Mg, Phosphate and CO_2 and Mann Whitney for Urea and Ca.
e, Lung total Ifnβ1 expression, WT (*n* = 17) and Akita (*n* = 7) mice, two-sided unpaired t-test.
f, WT (*n* = 27) and Akita (*n* = 14) mice infected with 50 pfu PR8, BAL inflammatory cytokines analyzed at 10 d.p.i., pooled data from 2 experiments, two-sided Mann Whitney U-test or unpaired t-test, as indicated in the supplementary p-value table.
g, WT (*n* = 27) and Akita (*n* = 14) mice infected with 50 pfu PR8, BAL inflammatory cytokines analyzed at 10 d.p.i., pooled data from 2 experiments, two-sided Mann Whitney U-test or unpaired t-test, as indicated in the supplementary p-value table.
All data mean+s.e.m.
NMR-Nuclear magnetic resonance.

Supplementary information 3

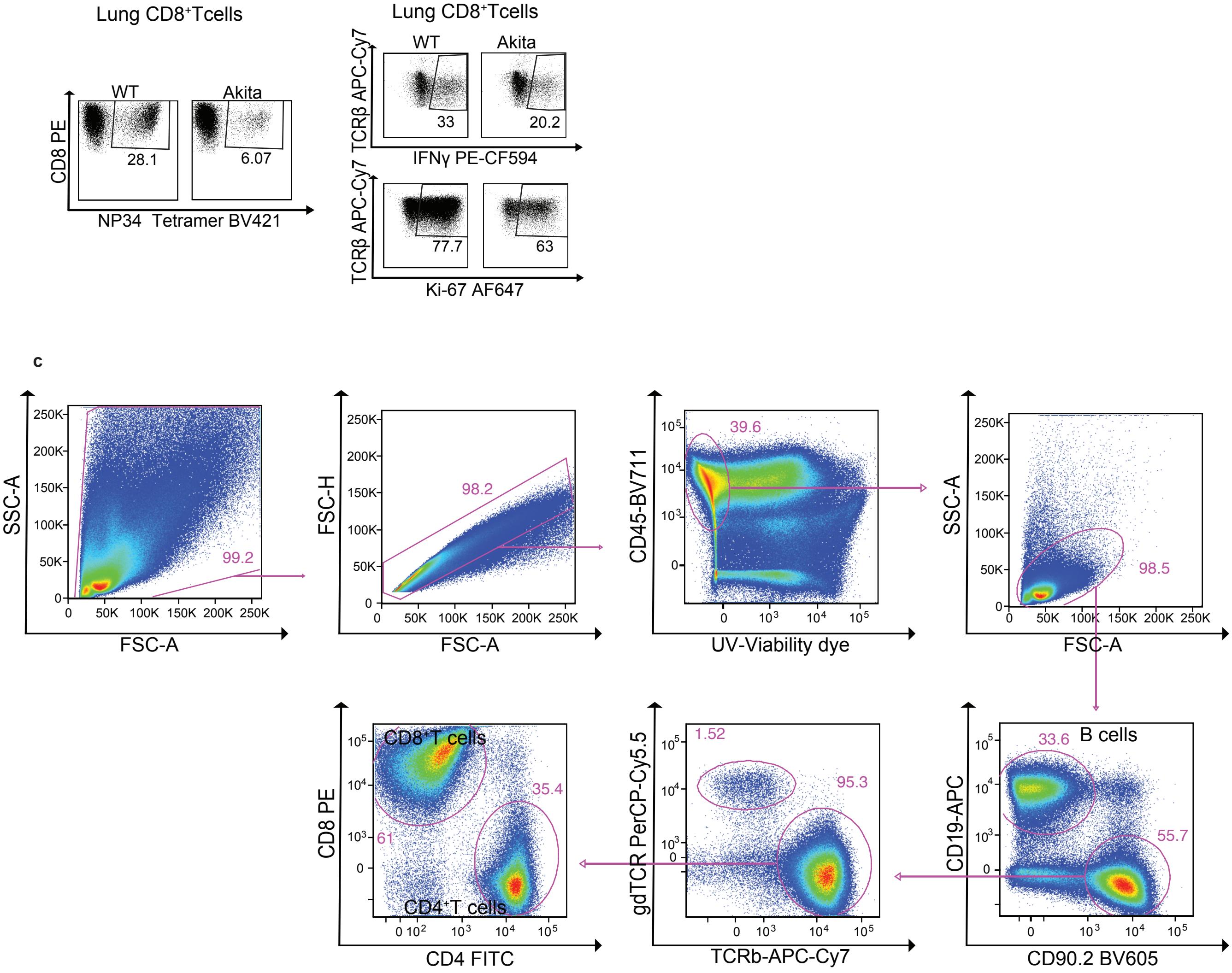

Supplementary information 3: Diabetes-associated susceptibility to respiratory viral infection is associated with broadly impaired adaptive immunity.

a-b, WT (*n* = 16) and Akita (*n* = 7) mice infected with 50 pfu PR8, lungs analyzed with flow cytometry at 10 d.p.i. (**a**) NP34 Tetramer+, (**b**) Ki-67+ and IFNγ+CD8+ T cells.

c, Gating strategy,T and B cells.

a

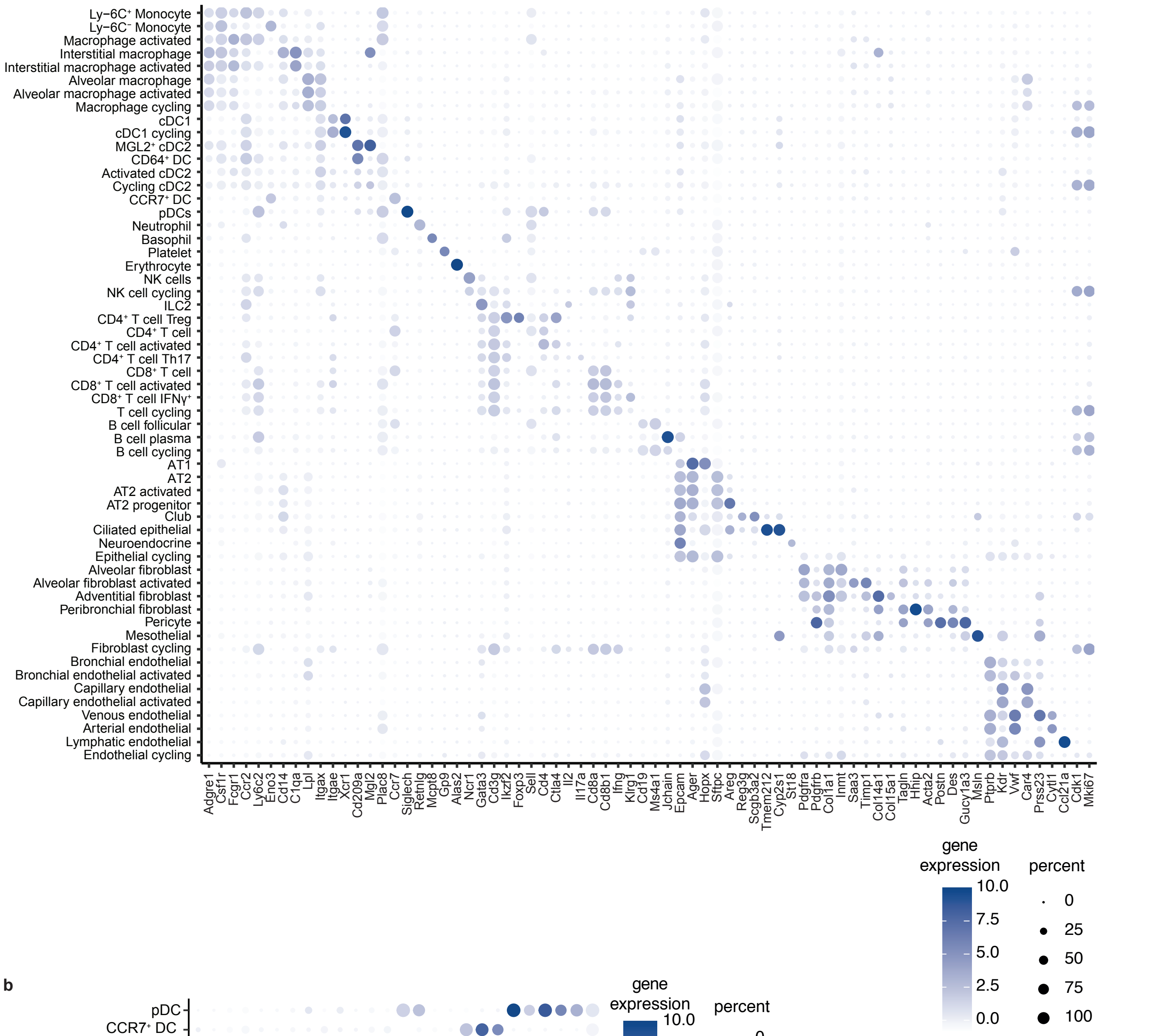

b

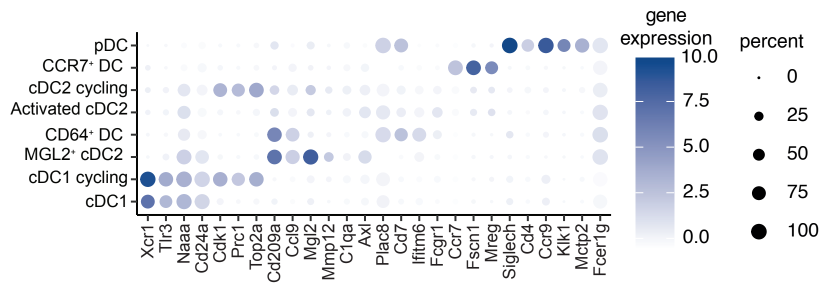

Supplementary information 4: Identification of lung cell types in steady-state and during PR8 infection.
a, Balloon plot of expression of key cell type markers of cell clusters in scRNA-seq (*n* = 4/group - steady-state and 10 d.p.i., *n* = 5/group - 1 d.p.i.). Dot size represents percentage of cells in which a gene was detected, color corresponds to the mean scaled gene expression of all cells in a cluster.
b, Balloon plot expression of scRNA-seq DC clusters. Dot size represents percentage of cells in which a gene was detected and color corresponds to the mean scaled gene expression of all cells in a cluster.
ILC2- type 2 innate lymphoid cell.

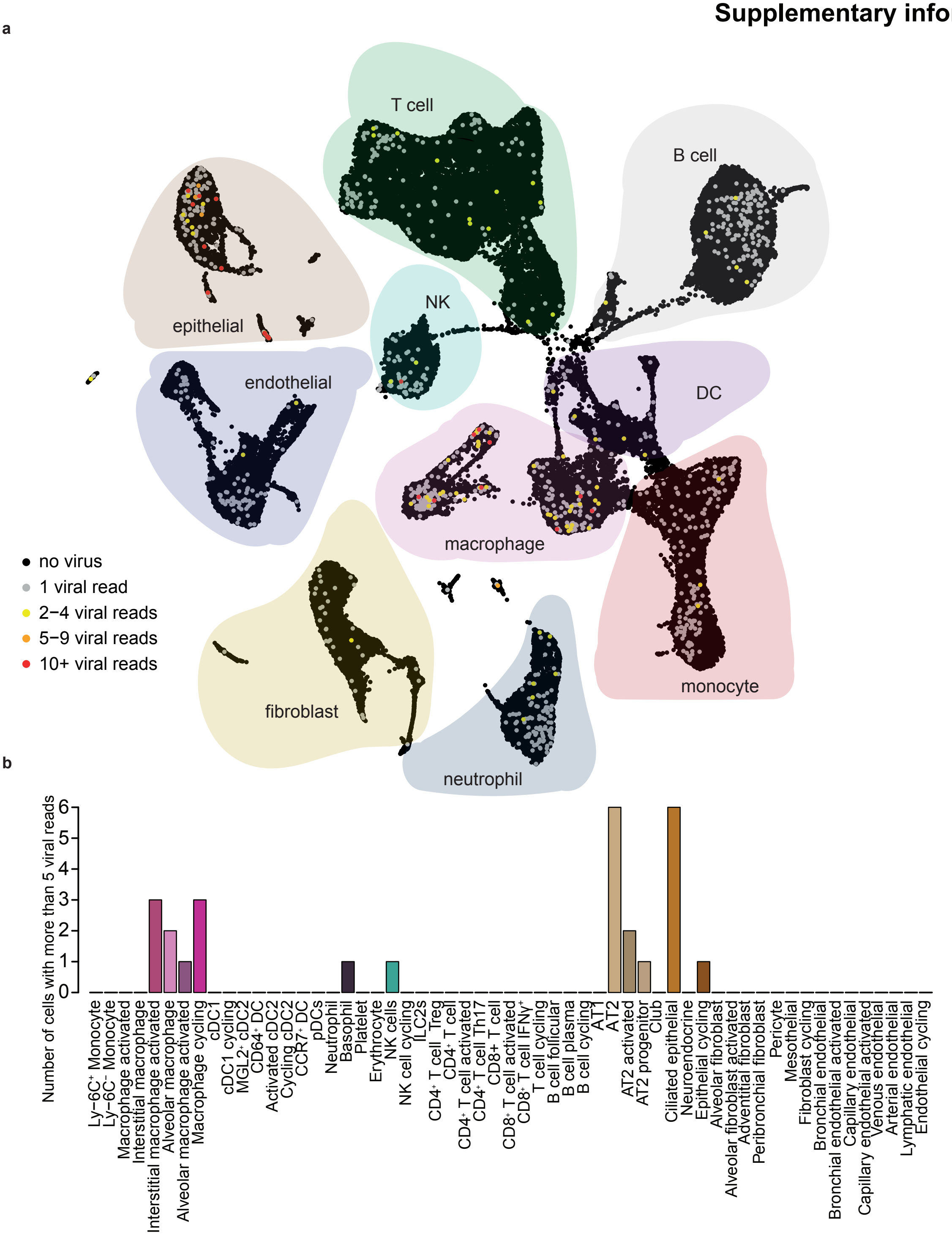

Supplementary information 5: Characterization of cell compartments in steady-state and during respiratory viral infection.
a-b, scRNA-seq of lungs of WT and Akita mice, collected during steady-state (*n* = 4 per group), 1 d.p.i. (*n* = 5 per group) and 10 d.p.i. (*n* = 4 per group) with PR8.
a, UMAP with cells in which reads map to viral genome, for cell types compared to Fig. 2a.
b, Bar plot with number of cells in each subset containing five reads or more that map to the viral genome.

Supplementary information 6

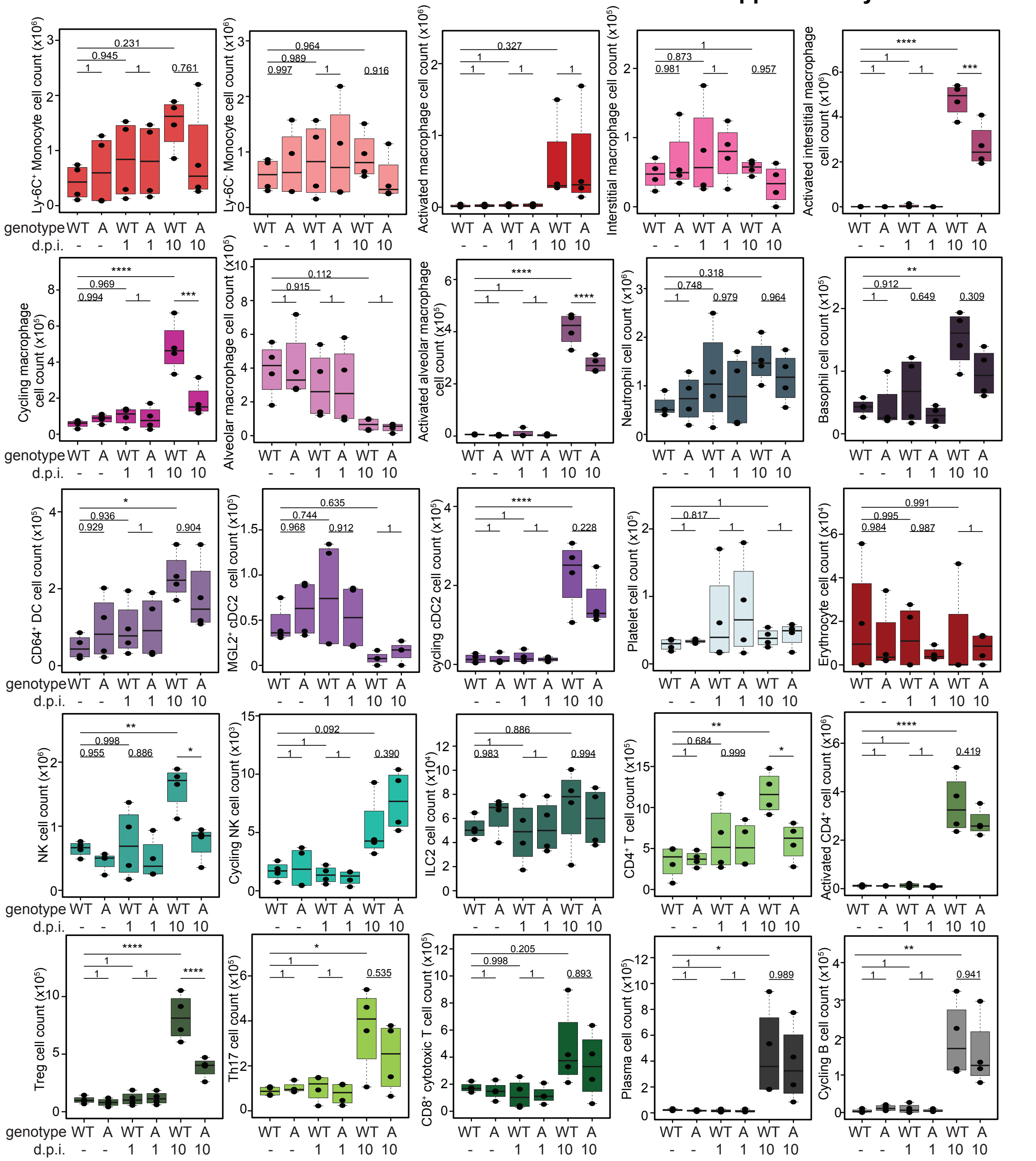

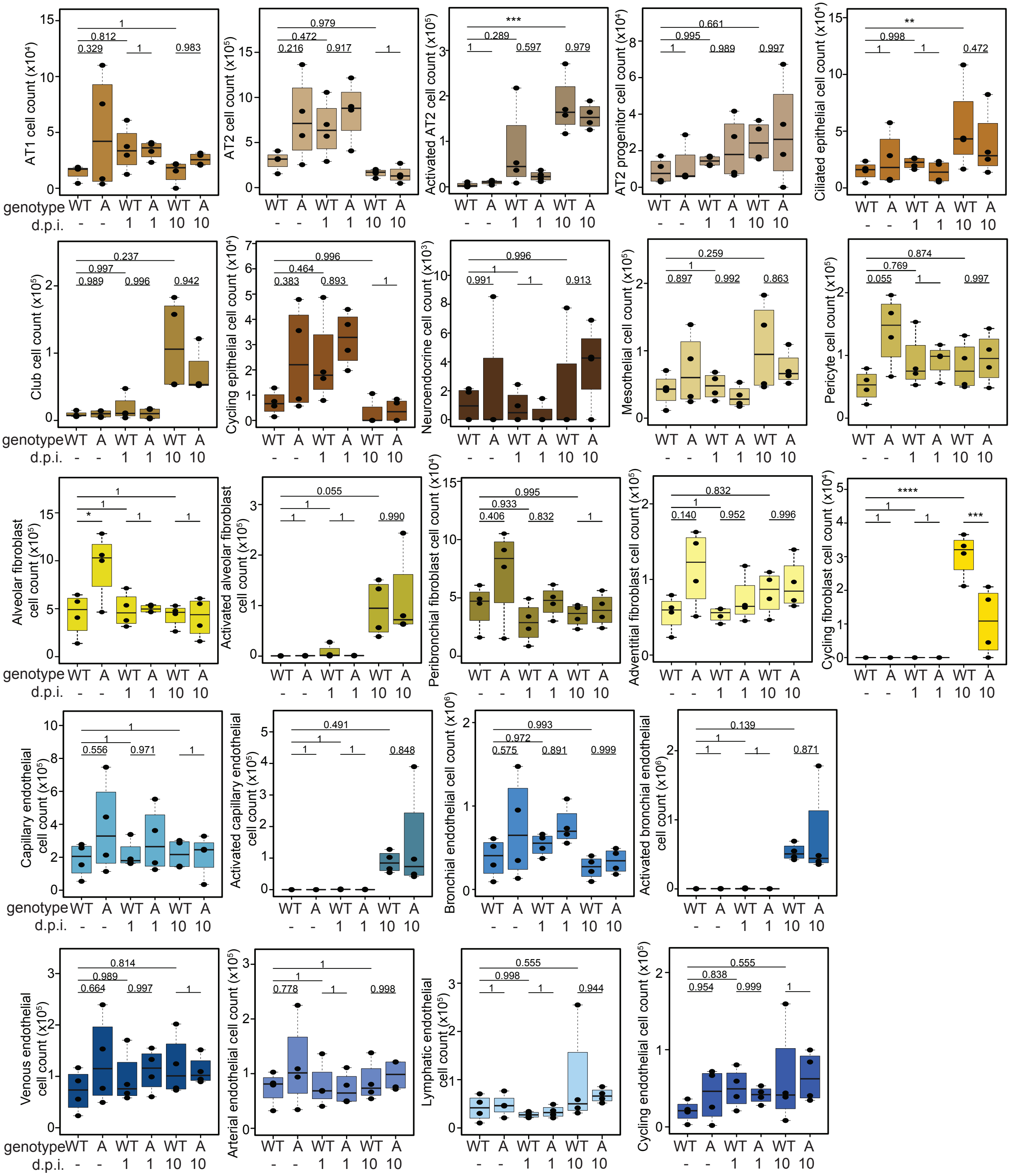

Supplementary information 6: Characterization of cell compartments in homeostasis and respiratory viral infection.
scRNA-seq of lungs of WT and Akita mice collected during steady-state (n = 4/group), 1 d.p.i. (n = 4/group), and 10 d.p.i. (n = 4/group) with PR8. Boxplots with populations not shown in Fig.2. Cell numbers obtained by multiplying cell type frequency from scRNA-seq data by total cell count, two-way ANOVA with Tukey test. Boxplots show 25th to 75th percentiles with the 50th percentile denoted by a thicker line; Whiskers show 1.5× interquartile range, or max. or min. if it is smaller than 1.5× interquartile range.

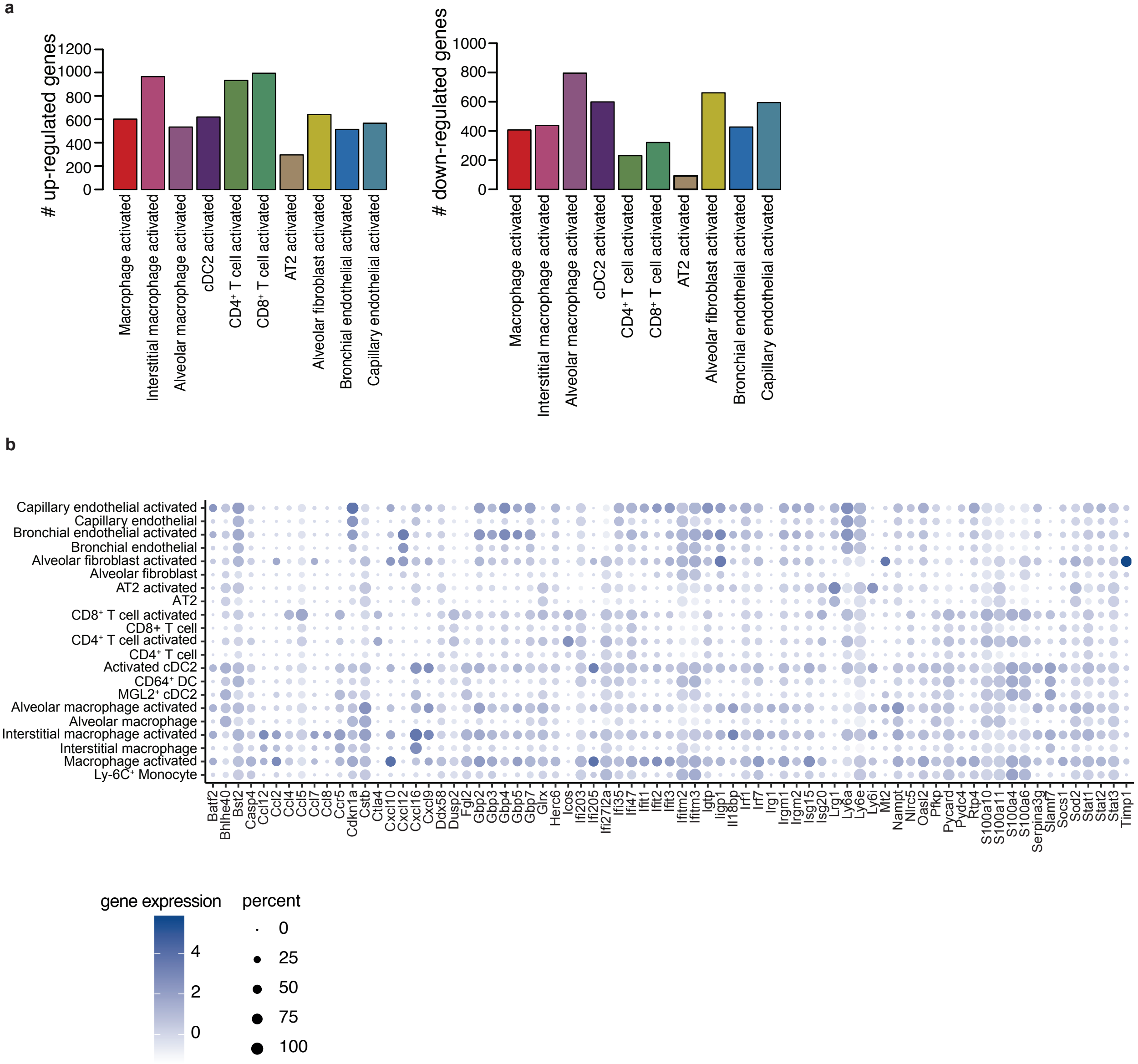

Supplementary information 7: Gene expression landscape changes in the lung during influenza infection.

a-b, scRNA-seq of lungs of WT and Akita mice collected during steady-state (*n* = 4/group), 1 d.p.i. (*n* = 5/group), and 10 d.p.i. (*n* = 4/group) with PR8.

a, Number of up- and down- regulated genes in activated cell populations compared to steady-state. Differential expression between cells in two clusters with Wilcoxon rank sum test on genes detected in at least 25% of cells in one of the populations, in which log-scale fold difference is at least 0.1. Differentially expressed genes: multiple hypothesis testing adjusted p-value using Bonferroni method (lower than 0.05).

b, Balloon plot of gene expression in activated cell populations with resting counterparts upregulated upon activation. The dot size represents percentage of cells in which gene was detected, and color corresponds to mean scaled gene expression of all cells in a cluster.

Supplementary information 8

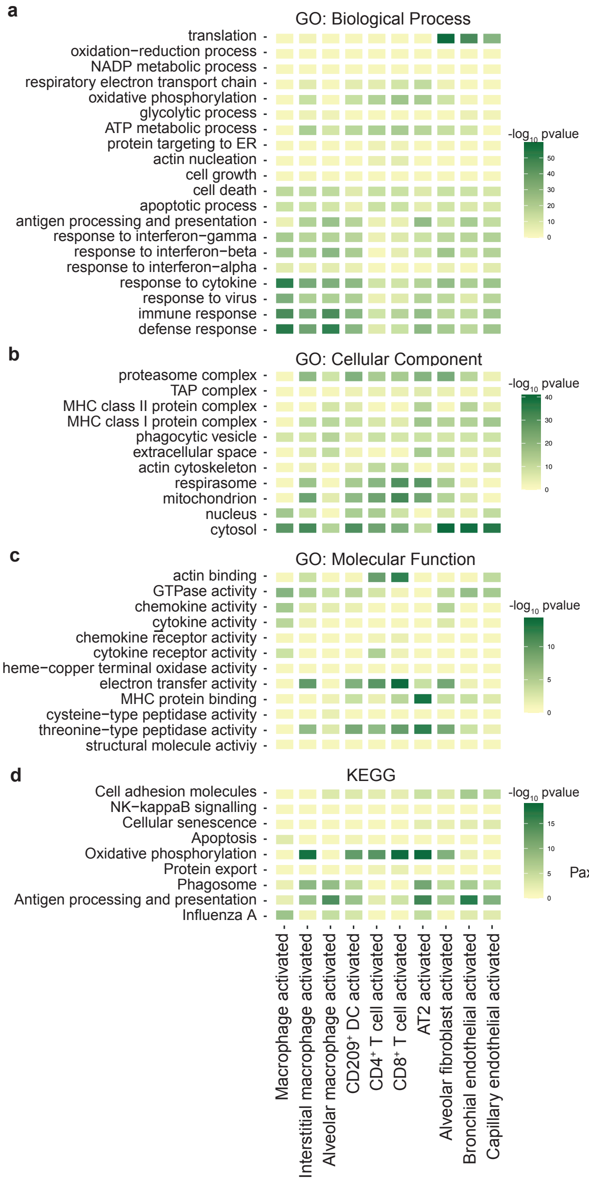

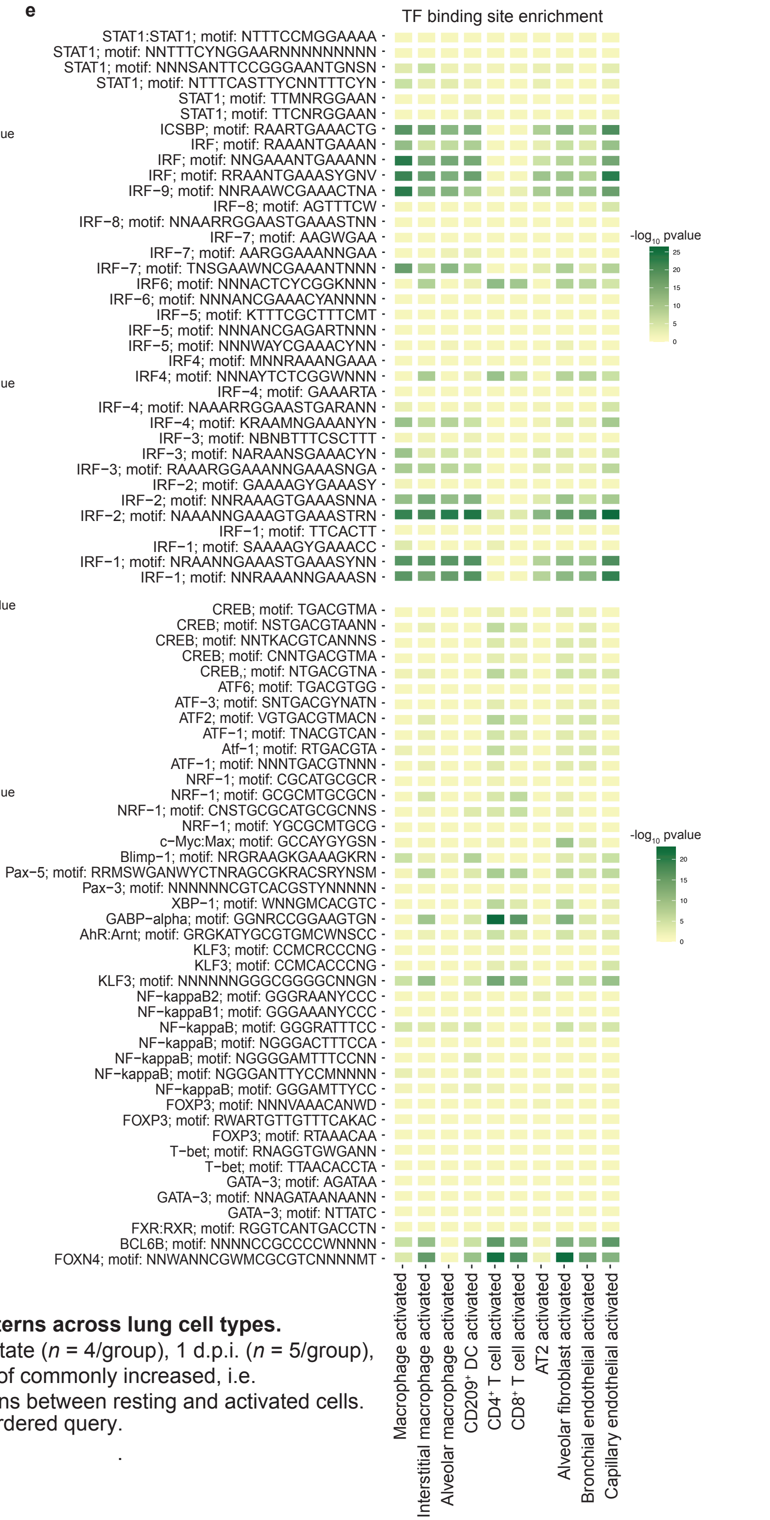

Supplementary information 8: Common activation patterns across lung cell types. scRNA-seq of lungs of WT and Akita mice during steady-state (n = 4/group), 1 d.p.i. (n = 5/group), and 10 d.p.i. (n = 4/group) with PR8. Enrichment analysis of commonly increased, i.e. at least differentially up-regulated in 5 out of 10 comparisons between resting and activated cells. GO analysis with gProfiler2 using standard settings, with ordered query.

a, top enriched terms in GO Biological Process.

b, top enriched terms in GO Cellular Component.

c, top enriched terms in GO Molecular Function.

d, top enriched terms in KEGG database.

e, transcription factor binding motifs enriched in promoters of up-regulated genes

p-values corrected for multiple hypothesis testing with g:SCS algorithm.

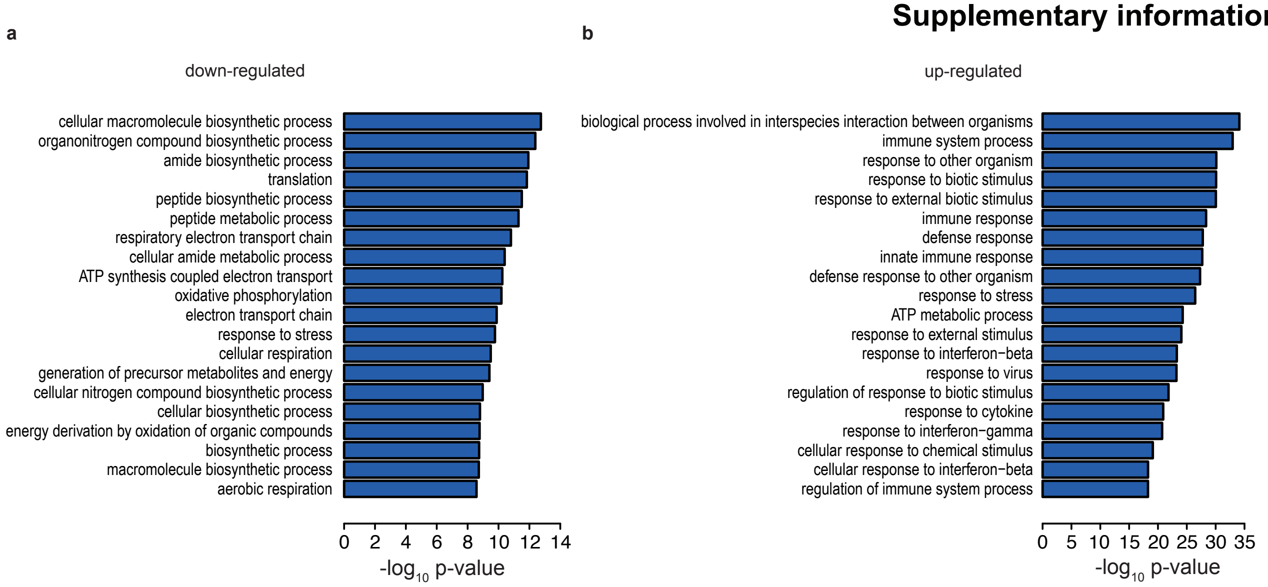

Supplementary information 9: Common activation patterns across lung cell types.
scRNA-seq of lungs of WT and Akita mice during steady-state (*n* = 4/group), 1 d.p.i. (*n* = 5/group), and 10 d.p.i. (*n* = 4/group) with PR8. **a-b**, GO Analysis of differentially **a**, downregulated and **b**, upregulated genes in resting compared to activated cells. GO analysis with gProfiler2 using standard settings, with ordered query; p-values corrected for multiple hypothesis testing using the g:SCS algorithm. Top 20 terms plotted.

Supplementary information 10

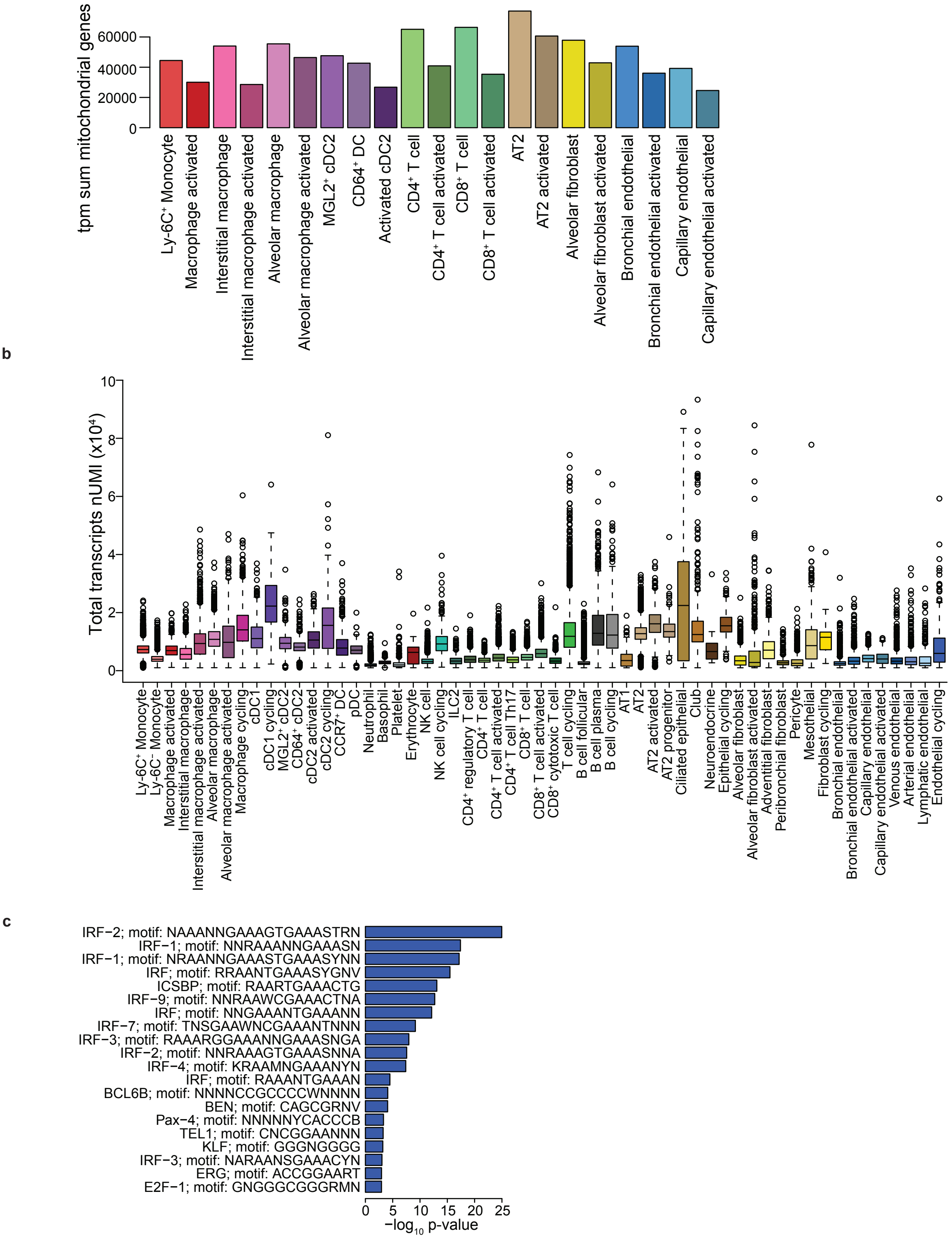

Supplementary information 10: Lung infection with PR8 leads to transcriptional modulation.
a-c, scRNA-seq of lungs of WT and Akita mice collected during steady-state (n = 4/group), 1 d.p.i. (n = 5/group), and 10 d.p.i. (n = 4/group) with PR8.
a, Bar plot with sum of transcripts per million (tpm) mapping to the mitochondrial genome in activated cells and resting counterparts.
b, Boxplots with number of transcripts (nUMI) per cell in all populations. Boxplots show 25th to 75th percentiles, with the 50th percentile denoted by a thicker line; Whiskers show 1.5× interquartile range, or max. or min. if smaller than 1.5× interquartile range.
c, TF binding sites enriched in the promoters of genes upregulated in at least 5 out of 10 populations upon activation.
TF binding site analysis with gProfiler2 using standard settings; p values corrected for multiple hypothesis testing with g:SCS algorithm.

Supplementary information 11

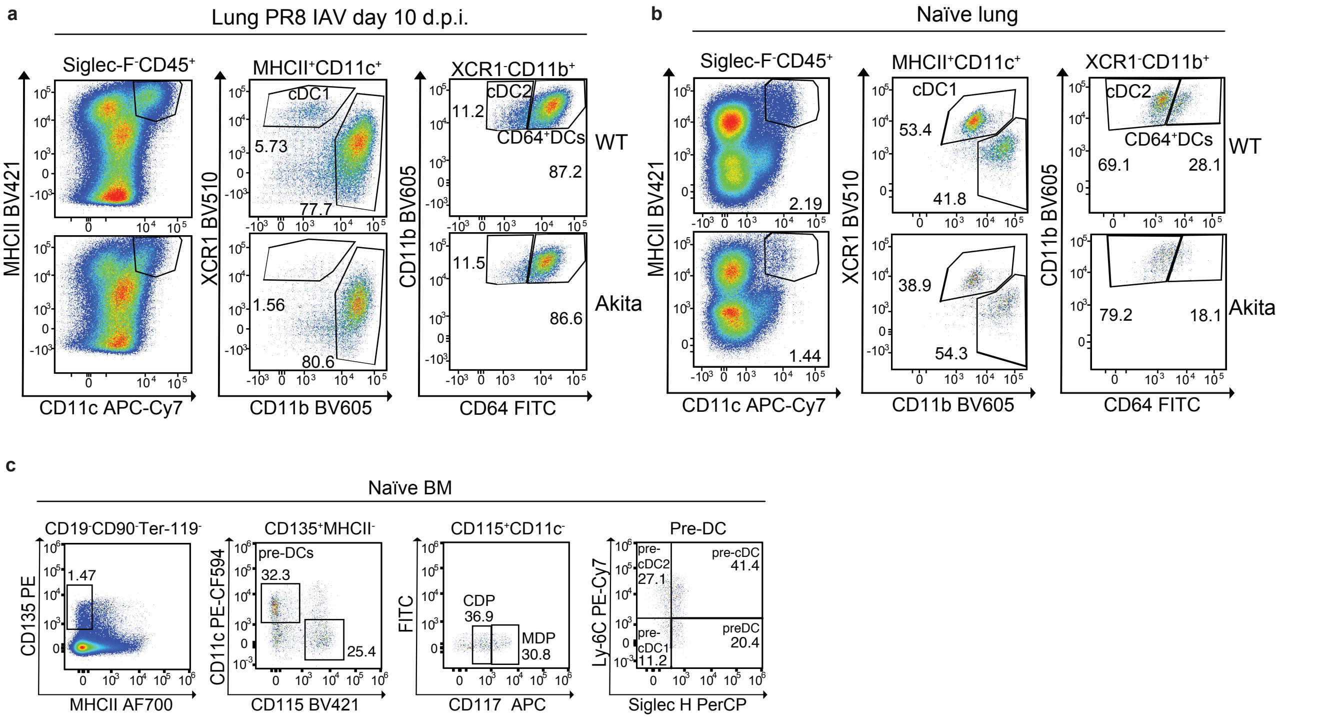

Supplementary information 11: Hyperglycaemia dysregulates the lung DC compartment in steady-state and during PR8 infection.
a-b, Gating strategy for lung DC in naïve and PR8-infected lungs.
c, Gating strategy for DC precursors in the BM.

a

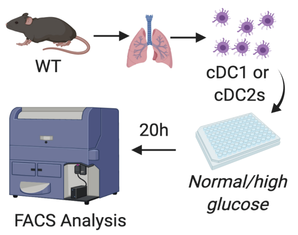

b

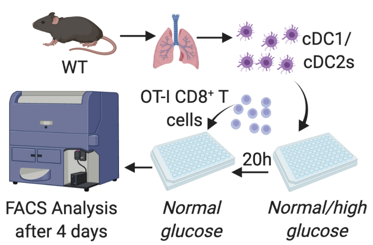

Supplementary information 12: Experimental schemes in studying the in vitro impact of high glucose on DC function.
a, Experimental scheme, WT Lung DC incubated with high (50mM) or normal (10mM) glucose.
b, Experimental scheme, WT Lung DC incubated with high (50mM) or normal (10mM) glucose for 20 h, followed by the addition of OT-I-$CD8^+$ T cells under normal (10mM) glucose conditions. Schemes created by Biorender.

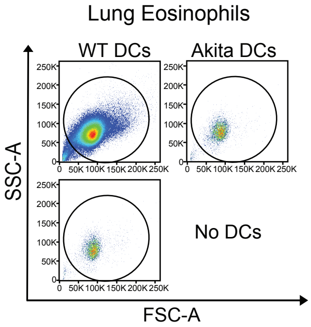

Supplementary information 13: Gating strategy, lung eosinophils.
Representative flow cytometry dot plots of lung eosinophils.

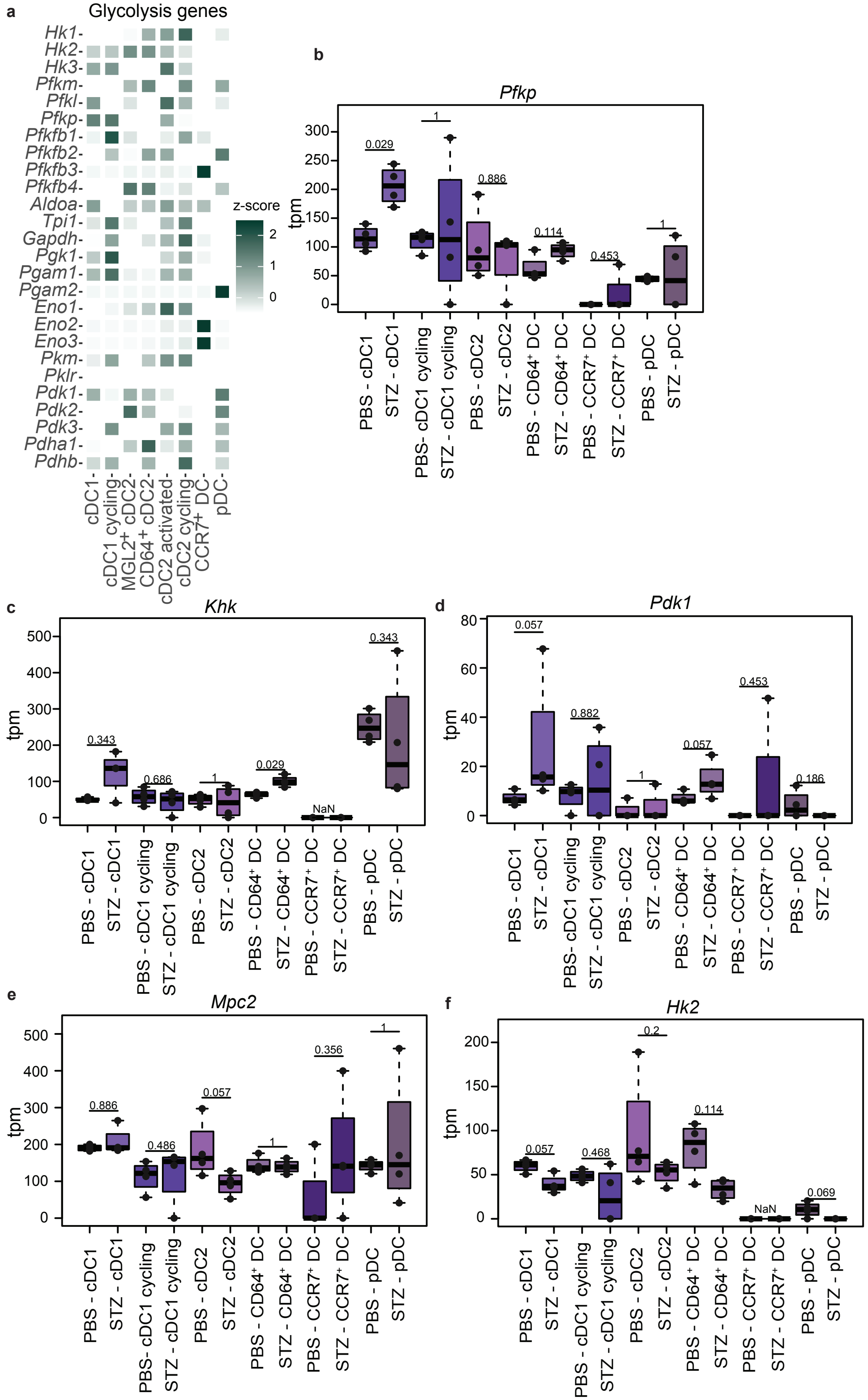

Supplementary information 14: Streptozotocin-induced hyperglycaemia dysregulates the lung DC compartment in steady-state.
a, scRNA-seq of lung DC, shown are DC subset expression patterns of genes encoding enzymes involved in glycolysis. Heatmaps represent z-scores of mean scaled and log normalized gene expression of cells in different DC populations. **b-f**, scRNA-seq of lung DC from PBS or STZ -administered mice (n = 4/group). Differentially expressed glycolytic metabolism genes are shown as Boxplots with tpm pseudobulk counts in $CD64^+$ DC, cDC1, cDC2, $CCR7^+$ DC and pDC, Wilcoxon rank sum test.

Supplementary Information 15

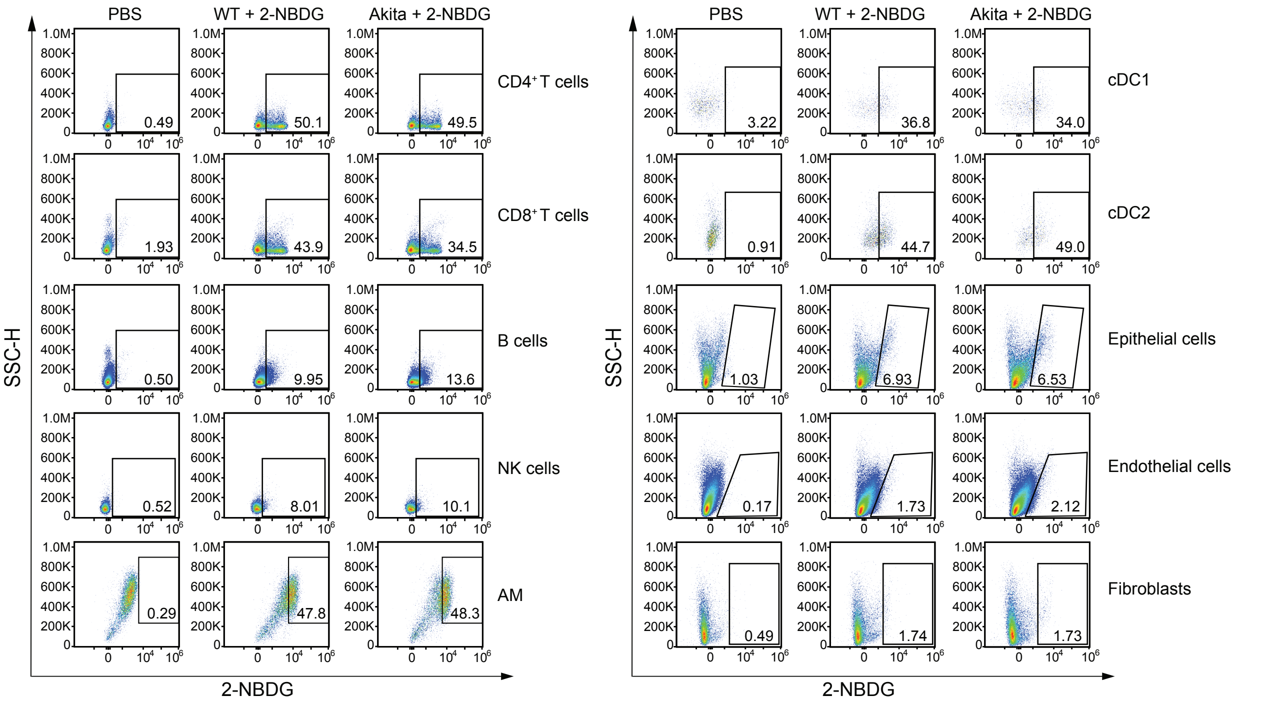

Supplementary information 15: Gating strategy for lung cell types in 2-NBDG experiments.
WT (*n* = 5) and Akita (*n* = 5) mice were intravenously injected with 5mM 2-NBDG. Exemplified is 2-NBDG uptake frequency and gating strategy in the indicated lung cell types.

Supplementary Information 16

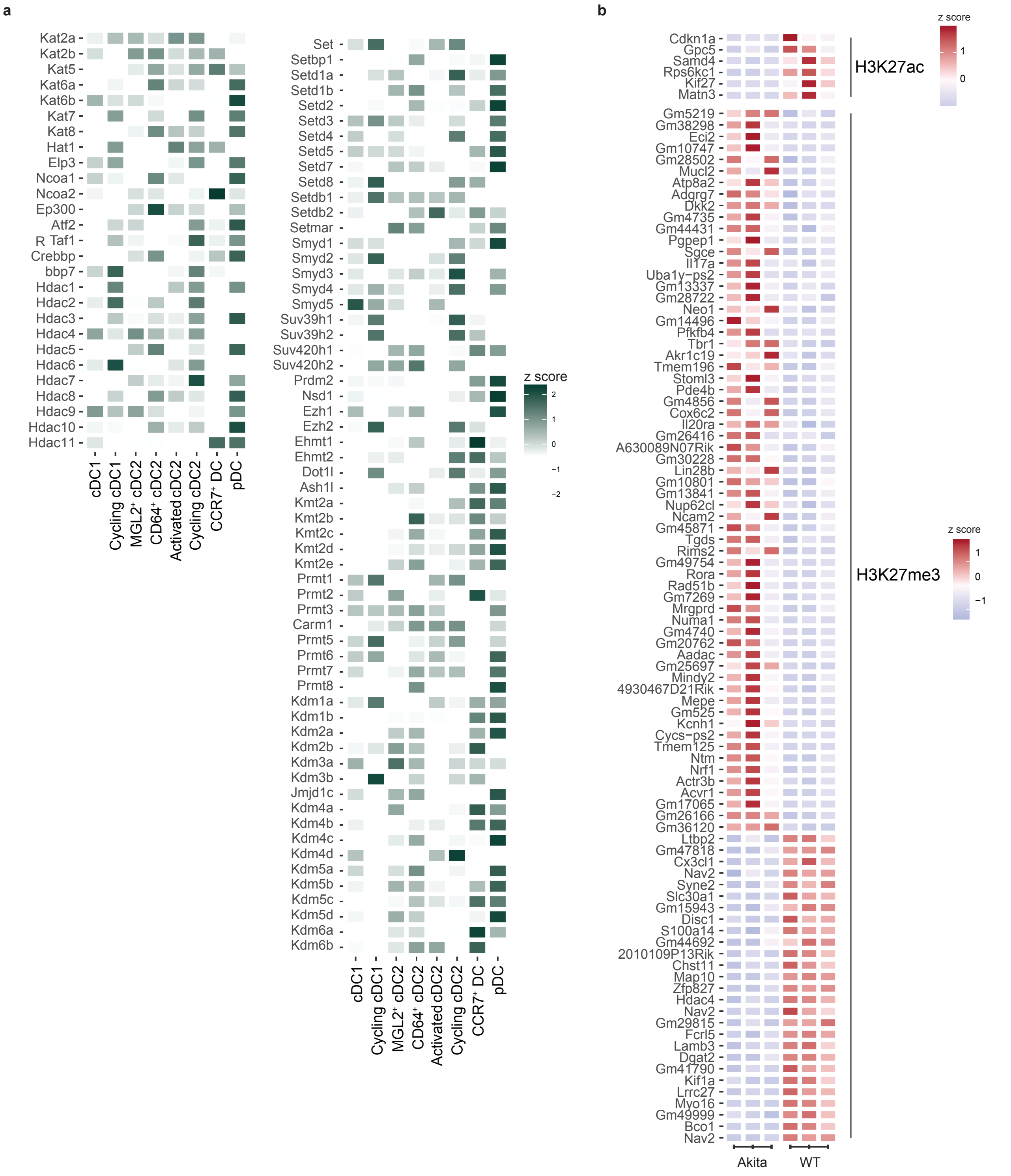

Supplementary information 16: Hyperglycaemia induces changes in acetylation and methylation in lung DC.
a, scRNA-seq of lungs of WT and Akita mice collected during steady-state (*n* = 4/group), 1 d.p.i. (*n* = 5/group), and 10 d.p.i. (*n* = 4/group) with PR8. Featured is a heatmap denoting gene expression of key acetylation and methylation enzymes of lung DCs.
b, H3K27me3 and H3K27ac profiling with CUT&Tag of lung DC from naïve WT and Akita mice. Heatmap of z-scores of number of normalized reads in peak in loci with differentially abundant H3K27me3 or H3K27ac (closest gene name indicated).
