# Supplementary material for: Lung dendritic-cell metabolism underlies susceptibility to viral infection in diabetes
Source: Nature. 2023 Dec 13;624(7992):645–52. doi: 10.1038/s41586-023-06803-0 (PMC10733144; doi:10.1038/s41586-023-06803-0)
Supplement: Supplementary file 2 — Reporting Summary [file 41586_2023_6803_MOESM2_ESM.pdf]

nature portfolio

Corresponding author(s): Eran Elinav

Last updated by author(s): Aug 7, 2023

Reporting Summary

Nature Portfolio wishes to improve the reproducibility of the work that we publish. This form provides structure for consistency and transparency in reporting. For further information on Nature Portfolio policies, see our Editorial Policies and the Editorial Policy Checklist.

Statistics

For all statistical analyses, confirm that the following items are present in the figure legend, table legend, main text, or Methods section.

n/a	Confirmed	
☐	☒	The exact sample size (*n*) for each experimental group/condition, given as a discrete number and unit of measurement
☐	☒	A statement on whether measurements were taken from distinct samples or whether the same sample was measured repeatedly
☐	☒	The statistical test(s) used AND whether they are one- or two-sided<br>*Only common tests should be described solely by name; describe more complex techniques in the Methods section.*
☐	☒	A description of all covariates tested
☐	☒	A description of any assumptions or corrections, such as tests of normality and adjustment for multiple comparisons
☐	☒	A full description of the statistical parameters including central tendency (e.g. means) or other basic estimates (e.g. regression coefficient) AND variation (e.g. standard deviation) or associated estimates of uncertainty (e.g. confidence intervals)
☐	☒	For null hypothesis testing, the test statistic (e.g. F, t, r) with confidence intervals, effect sizes, degrees of freedom and P value noted<br>*Give P values as exact values whenever suitable.*
☒	☐	For Bayesian analysis, information on the choice of priors and Markov chain Monte Carlo settings
☒	☐	For hierarchical and complex designs, identification of the appropriate level for tests and full reporting of outcomes
☒	☐	Estimates of effect sizes (e.g. Cohen's *d*, Pearson's *r*), indicating how they were calculated

Our web collection on statistics for biologists contains articles on many of the points above.

Software and code

Policy information about availability of computer code

Data collection	Diva
Data analysis	bcl2fastq 2.20.0.422, CellRanger 6.0.0, CellRanger-ATAC 2.0.0, R. 4.0.0, Seurat 4.0.6, DESeq2 1.24.0, gprofiler2 0.2.1, Signac 1.5.0, ggplot2 3.3.6, biomaRt 2.50.0, fastp 0.23.0, bowtie2/2.3.5.1, samtools 1.9, picard 2.22.8, bedtools 2.26.0, SEACR 1.3 GraphPad Prism 9, flowJo 8.8.6

For manuscripts utilizing custom algorithms or software that are central to the research but not yet described in published literature, software must be made available to editors and reviewers. We strongly encourage code deposition in a community repository (e.g. GitHub). See the Nature Portfolio guidelines for submitting code & software for further information.

Data

Policy information about availability of data

All manuscripts must include a data availability statement. This statement should provide the following information, where applicable:

- Accession codes, unique identifiers, or web links for publicly available datasets
- A description of any restrictions on data availability
- For clinical datasets or third party data, please ensure that the statement adheres to our policy

All raw sequencing data are deposited to Array Express with following accession numbers. scRNAseq raw data from WT and Akita mice over the course of infection - E-MTAB-11394; hashed scRNAseq data of dendritic cells from STZ model - E-MTAB-11393; CUT&RUN data - E-MTAB-11390

Field-specific reporting

Please select the one below that is the best fit for your research. If you are not sure, read the appropriate sections before making your selection.

☒ Life sciences ☐ Behavioural & social sciences ☐ Ecological, evolutionary & environmental sciences

For a reference copy of the document with all sections, see nature.com/documents/nr-reporting-summary-flat.pdf

Life sciences study design

All studies must disclose on these points even when the disclosure is negative.

Sample size	The minimal sample size for each experiment was set by the minimal number of mice allowed in each cage, taking into account for each group at least two cages were included to minimize the cage effect. We took into account the variability withing a cage and experimental group as well as between individual experimental repetitions to set appropriate sample numbers to allow for sound interpretation of experimental results
Data exclusions	No data was excluded.
Replication	We used 2 to 7 replications for single experiments and all replications were successful
Randomization	For each experiment, mice were randomly assigned to each group
Blinding	Experiments were blinded where observer bias affects results including histology analysis which was scored blindly by trained pathologist

Reporting for specific materials, systems and methods

We require information from authors about some types of materials, experimental systems and methods used in many studies. Here, indicate whether each material, system or method listed is relevant to your study. If you are not sure if a list item applies to your research, read the appropriate section before selecting a response.

Materials & experimental systems

n/a	Involved in the study	
☐	☒	Antibodies
☒	☐	Eukaryotic cell lines
☒	☐	Palaeontology and archaeology
☐	☒	Animals and other organisms
☒	☐	Human research participants
☒	☐	Clinical data
☒	☐	Dual use research of concern

Methods

n/a	Involved in the study	
☐	☒	ChIP-seq
☐	☒	Flow cytometry
☒	☐	MRI-based neuroimaging

Antibodies

Antibodies used

1. Anti-CD45-AF700 (Biolegend, 30-F11, Cat. 103128)
2. Anti-CD45.2- BV711 (Biolegend, 104, Cat. 109847)
3. Anti-CD4-PerCP Cy 5.5 (Biolegend, RM45, Cat. 100540)
4. Anti-CD8a-BV605 (Biolegend, 5367, Cat. 100743)
5. Anti-NK1.1-APC (Biolegend, PK136, Cat.108710)
6. CD86 PE-CF594 (Biolegend, GL-1, cat. 105042)
7. Anti-F4/80-PE-Cy7 (Biolegend, CIA31, cat. 123114)
8. Anti-CD11b-BV605 (Biolegend, M1/70, Cat-101257)
9. Anti-CD11c-APC-Cy7 (Biolegend, N418, cat. 117324)
10. Anti-I-A/I-E (MHCII)-BV421 (Biolegend, M5/114152, Cat. 107632)
11. Anti-Ly-6G-PerCP Cy5.5 (Biolegend , 1A8, Cat-127616)
12. Anti-Ly-6C-FITC (Biolegend, HK14, cat. 128005)
13. IFNg PE-CF594 (Biolegend, HK14, cat. 505846)
14. Siglec-F (E50-2440, BD Biosciences, cat. 552126)
15. IL-4 PE (Biolegend, 11B11, cat. 504104)
16. IL-5 BV421 (Biolegend, TRFK5 cat. 504311)
17. CD90.2 BV605 (Biolegend, 30-H12 cat. 105343)
18. FcεRIα PE (MAR-1, Thermo Fisher Scientific, cat. 12-5898-82)
19. XCR1 BV510 (Biolegend, ZET, cat. 148218)
20. GATA3 BV421 (Biolegend, 16E10A23 cat. 653814)
21. RORγt PE (AFKJS-9, Thermo Fisher Scientific, cat. 12-6988-82)
22. IL-17A PerCP-Cy5.5 (Biolegend, TC1118H10.1, cat. 506920)

23. IL-13 AF488 (eBioscience, eBio13A, cat. 53-7133-82)
24. T-bet PE-Cy7 (Biolegend, 4B10, cat. 644824)
25.FoxP3 PE (Biolegend, MF-14, cat. 126404)
26. CD40 PerCP(Biolegend, 3/23 cat. 124624)
27. CD80 PE (Biolegend, 16-10A1, cat. 104708)
28. Goat anti-rabbit AF647 (Thermofisher, cat. A-21245)
29. Rat anti-mouse CD16/32 (Biolegend cat. 101319)

Validation

The antibodies meant for FACS were all tested for use in mice and verified by manufacturers. They were used in many studies, as detailed in the manufacturers website:

https://www.biolegend.com: To confirm antibody specificity, Western blot data using BioLegend's in-house generated CRISPR/Cas9 and siRNA, as well as CRISPR/Cas9 KO cell lysates from a collaboration with EdiGene (a genome editing company) are used.

https://www.thermofisher.com:

Part 1—Target specificity verification

This helps ensure the antibody will bind to the correct target. Our antibodies are being tested using at least one of the following methods to ensure proper functionality in researcher's experiments. Click on each testing method below for detailed testing strategies, workflow examples, and data figure legends.

Knockout—expression testing using CRISPR-Cas9 cell models
Knockdown—expression testing using RNAi to knockdown gene of interest
Independent antibody verification (IAV)—measurement of target expression is performed using two differentially raised antibodies recognizing the same protein target
Cell treatment—detecting downstream events following cell treatment
Relative expression—using naturally occurring variable expression to confirm specificity
Neutralization—functional blocking of protein activity by antibody binding
Peptide array—using arrays to test reactivity against known protein modifications
SNAP-ChIP™—using SNAP-ChIP to test reactivity against known protein modifications
Immunoprecipitation-Mass Spectrometry (IP-MS)—testing using immunoprecipitation followed by mass spectrometry to identify antibody targets

Part 2—Functional application validation
These tests help ensure the antibody works in a particular application(s) of interest, which may include
(but are not limited to): Western blotting, Flow cytometry, ChIP, Immunofluorescence imaging, Immunohistochemistry
Most antibodies were developed with specific applications in mind. Testing that an antibody generates acceptable results in a specific application is the second part of confirming antibody performance.

https://www.bdbiosciences.com:
BD Biosciences identifies key targets of interest in scientific research and develops its own specific antibodies or collaborates with top research scientists around the world to license their antibodies. We then transform these antibodies into flow cytometry reagents by conjugating them to a broad portfolio of high-performing dyes, including our vastly popular portfolio of BD Horizon Brilliant™ Dyes.
A world-class team of research scientists helps ensure that these reagents work reliably and consistently for flow cytometry applications.
The specificity is confirmed using multiple methodologies that may include a combination of flow cytometry, immunofluorescence, immunohistochemistry or western blot to test staining on a combination of primary cells, cell lines or transfectant models.

Animals and other organisms

Policy information about studies involving animals; ARRIVE guidelines recommended for reporting animal research

Laboratory animals

All mice were used between 8 and 14 weeks of age.
C57BL6 SPF wild type mice, male
Akita mice, male (C57BL/6-Ins2Akita/J)
Pdk2/3/4 knockout mice male
OT-I mice male
OT-II mice male
Db/Db mice male

Wild animals

No wild animals were used in the study.

Field-collected samples

No field collected samples were used in the study.

Ethics oversight

All experiments were performed in accordance with institutional and European guidelines and were approved by the Weizmann Institute of Science IACUC committee, IACUC no 05400622-2, 02800321-1, 04000520-2, 14760619-3.

Note that full information on the approval of the study protocol must also be provided in the manuscript.

ChIP-seq

Data deposition

☒ Confirm that both raw and final processed data have been deposited in a public database such as GEO.

☐ Confirm that you have deposited or provided access to graph files (e.g. BED files) for the called peaks.

Data access links<br>*May remain private before publication.*	CUT&RUN data - E-MTAB-11390
Files in database submission	fastq files, BED files
Genome browser session<br>(e.g. UCSC)	NA

Methodology

Replicates	3 biological replicates per group
Sequencing depth	average sequencing depth for each antibody: H3K27me3 121697706 H3K27ac 14928723 Isotype control 3920200<br>average uniquely mapped for each antibody: H3K27me3 33538535 H3K27ac 2347728 Isotype control 515832<br>sequencing: PE 51bp each read
Antibodies	H3K27me3 antibody, C36B11, Cell Signalling<br>H3K27ac antibody, D5E4, Cell Signalling<br>Isotype control antibody, DA1E, Cell Signalling
Peak calling parameters	Peaks were called with SEACR v1.3 using isotype control data as background and stringent peak calling method and identified genes closest to the peaks.
Data quality	Data QC was performed with FastQC and then trimmed (fastp) and deduplicated (picard)
Software	For the analysis Bcl files were demultiplexed and converted to fastq files with bcl2fastq v.2.20.0.422. Subsequently, reads were trimmed to remove adaptors using fastp v0.23.0 with standard parameters. Mapping to the GRCm38 genome was done using bowtie2 v2.3.4.1 and following parameters --local --very-sensitive-local --no-unal --no-mixed --no-discordant --phred33 -I 10 -X 700 and deduplicated with picard v2.22.8.Files were converted with samtools v1.9 and bedtools 2.26.0 to generate bedgraph files. Peaks were called with SEACR v1.3 using isotype control data as background and identified genes closest to the peaks. Data was subsampled to have the same coverage across samples and reads in peaks were counted with bedtools multicov. To find differentially abundant peaks, we used DESeq2.

Flow Cytometry

Plots

Confirm that:

☒ The axis labels state the marker and fluorochrome used (e.g. CD4-FITC).

☒ The axis scales are clearly visible. Include numbers along axes only for bottom left plot of group (a 'group' is an analysis of identical markers).

☒ All plots are contour plots with outliers or pseudocolor plots.

☒ A numerical value for number of cells or percentage (with statistics) is provided.

Methodology

Sample preparation	Mice were sacrificed by i.p. injection of 200mg/ml sodium pentobarbital. Lungs were perfused with cold PBS and put on ice after removal. Lung draining lymph nodes were collected and then digested with 2 mg/ml of type IV collagenase (Worthington) and 1 mg/ml DNaseI (Sigma) at 37 °C for 20 min in IMDM and subsequently passed through a 70 µm cell strainer using 10 ml PBS. Lungs were minced and digested with 1 mg/ml Hyaluronidase (Sigma), 25 µg/ml Collagenase XI (Sigma), 50 µg/ml Liberase TM (Roche) and 1 mg/ml DNaseI (Sigma) in IMDM at 37 °C for 30 min and subsequently passed through a 70 µm cell strainer using 20 ml PBS. The cells were then centrifuged for 10 min at 500 g and resuspended with 10 ml PBS. The cells were centrifuged 7 min at 500 g before resuspension in 1 ml PBS
Instrument	BD LSRFortessa, BD LSR2, BD ARIA III, Attune Flow Cytometer
Software	FlowJo
Cell population abundance	The abundance of the cell population depended on the cell type, quantification of all analyzed cell types are given in the manuscript.

Gating strategy

Cells were identified the following way: CD4+T cells as CD45+TCRb+CD4+, CD8+ T cells as CD45+TCRb+CD8+, B cells as CD45+CD19+TCRb- cells, eosinophils as CD45+CD11c-CD11b+Siglec-F+SSC-Ahigh, neutrophils as CD45+CD11c-CD11b+Ly-6G+ cells, lung cDC1 as Siglec-F-MHCII+CD11c+XCR1+, lung cDC2 as Siglec-F-MHCII+CD11c+XCR1-CD11b+CD64- and lung CD64+DC as Siglec-F-MHCII+CD11c+XCR1-CD11b+CD64+ cells, dLN cDC2 as CD45+autofluorescent-CD11c+MHCIIhighXCR1+, lung dLN cDC1 as CD45+autofluorescent-CD11c+MHCIIhighXCR1-CD11b+CD64- and Ly-6Chigh monocytes as Siglec-F-Ly-6G-CD11b+Ly-6Chigh and Ly-6Clow monocytes as Siglec-F-Ly-6G-CD11b+Ly-6Clow.

☒ Tick this box to confirm that a figure exemplifying the gating strategy is provided in the Supplementary Information.
